# Engineering Cell Fate with Adaptive Feedback Control

**DOI:** 10.1021/acssynbio.5c00299

**Published:** 2025-07-23

**Authors:** Frank Britto Bisso, Giulia Giordano, Christian Cuba Samaniego

**Affiliations:** † Computational Biology Department, 6612 Carnegie Mellon University, Pittsburgh, Pennsylvania 15213, United States; ‡ Department of Industrial Engineering, 19034 University of Trento, Trento 38123, Italy

**Keywords:** genetic circuits, incoherent feedforward loop, adaptive control, feedback control, cell fate, multistability

## Abstract

Engineering cell fate is fundamental to optimizing therapies based on stem cells, which are aimed at replacing cells in patients suffering from trauma or disease. By timely administering molecular regulators (e.g., transcription factors, RNAs, or small molecules) in a process that mimics in vivo embryonic development, stem cell differentiation can be guided toward a specific cell fate. However, scaling up these therapies is extremely challenging because such differentiation strategies often result in mixed cellular populations. While synthetic biology approaches have been proposed to increase the yield of desired cell types, designing gene circuits that effectively redirect cell fate decisions requires mechanistic insight into the dynamics of the endogenous regulatory networks that govern this type of decision-making. In this work, we present a biomolecular adaptive controller designed to favor a specific cell fate. The controller, whose topology is akin to that of an Incoherent Feedforward Loop (IFFL), requires minimal knowledge of the endogenous network as it exhibits adaptive, non-reference-based behavior. The synthetic circuit operates through a sequestration mechanism and a delay introduced by an intermediate species, producing an output that asymptotically approximates a discrete temporal derivative of its input if the sequestration rate is sufficiently fast. Allowing the controller to actuate over a target species involved in the decision-making process creates a tunable synthetic bias that favors the production of the desired species with minimal alteration to the overall equilibrium landscape of the endogenous network. Through theoretical and computational analysis, we provide design guidelines for the controller’s optimal operation, evaluate its performance under parametric perturbations, and extend its applicability to various examples of common multistable systems in biology.

## Graphical abstract

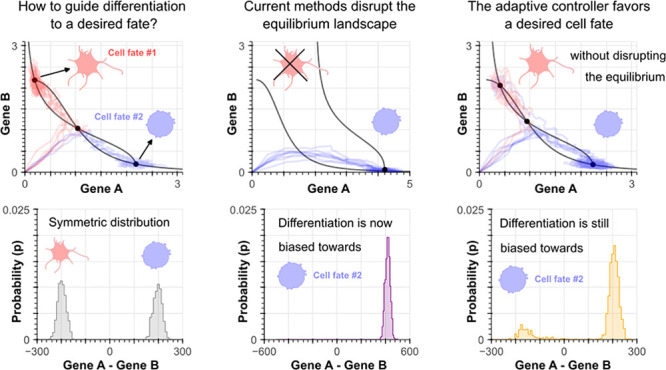

## 1 Introduction

In multicellular organisms, phenotypical diversity is a direct consequence of cell decision-making, where cells in a transient (e.g., undifferentiated) state integrate and process information from their environment, and respond by committing to a specific fate.^1,2^ For example, during embryonic development, pluripotent stem cells are primarily responsible for generating cellular diversity. They give rise to the primordial germ layers—mesoderm, endoderm, and ectoderm—which then further specialize into all the tissues within our body.^3^ Historically, the theoretical framework used to understand cell decision-making is Waddington’s epigenetic landscape (Figure 1A). In this model, each decision is depicted as a bifurcation point, with branching pathways cascading until an asymptotically stable equilibrium is reached, representing the cell’s ultimate fate (see Supplementary Text). This visual representation provides insights for controlling cell fate: the use of ectopic molecular regulators (e.g., transcription factors, RNAs, and small molecules) can induce a cell to follow a path toward a specific equilibrium.

**Figure 1:**
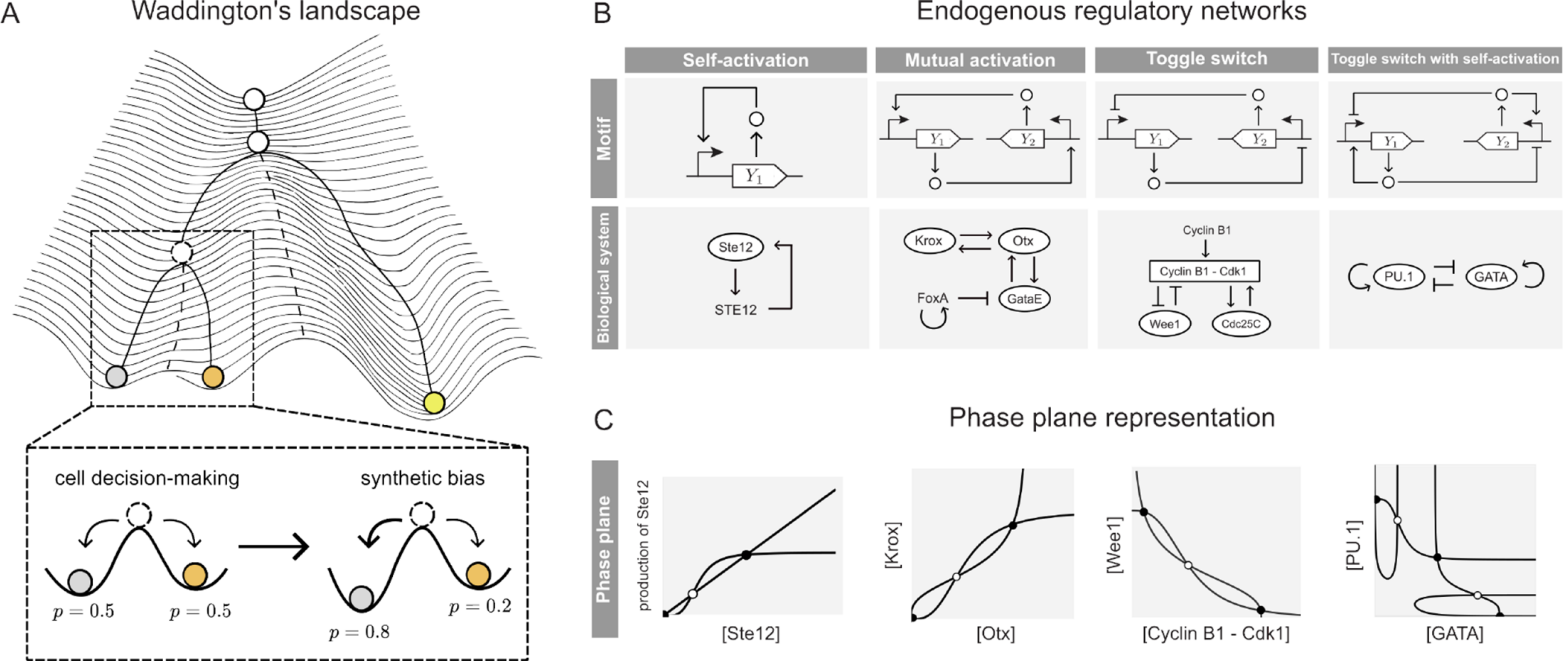
Multistability in endogenous biological systems. (A) The schematic of the Waddington’s epigenetic landscape, inspired by the original drawing,^50^ illustrates how a pluripotent stem cell undergoes a cascade of decisions (bifurcations) that ultimately determine its fate (asymptotically stable equilibrium). We consider the case of an unstable equilibrium from which a perturbation can lead to either of two asymptotically stable equilibria with the same probability, and design an adaptive feedback controller that introduces a synthetic bias to favor one equilibrium (cell fate) over the other. (B) Well-documented networks that exhibit multistability: (I) self-induced upregulation of the Ste12 gene upon exposure to mating pheromones,^51^ representative of the “self-activation” motif; (II) mutual activation between Kros, Otx and GataE genes during endomesoderm specification,^52^ representative of the “mutual activation” motif; (III) mutual inhibition in the “mitotic trigger”,^53^ representative of the “toggle switch” motif; and (IV) mutual inhibition with positive self-activation in a gene regulatory network of hematopoietic differentiation,^54^ representative of the “toggle switch with self-activation” motif. (C) Phase plane representation of the endogenous regulatory networks detailed in Panel B, where the curves represent the nullclines of the associated dynamical system and their intersections correspond to the system equilibria, denoted as black dots when asymptotically stable and as white dots when unstable.

In the context of cell-based therapeutics for tissue replacement, developmental pathways are considered a discrete sequence of decision-making processes.^4^ Stem cell differentiation can then be guided toward a desired fate by appropriately timing the activation or inhibition of key signaling pathways to mimic in vivo development. This approach led to the development of pancreatic islet replacement therapies for restoring normal glycemic control in diabetic patients, with currently an FDA-approved treatment for type 1 diabetes,^5^ and a promising case report for diabetes type 2;^6^ as well as stem-cell-derived dopaminergic neuron replacement as an alternative treatment to Parkinson’s disease, with ongoing clinical trials.^7,8^ A current challenge in scaling up the manufacturing of these therapies is that guided differentiation strategies often lead to mixed populations, producing both desired cell types and off-target cells due to diverging cell fates. This issue can be traced back to specific (undifferentiated) progenitor cells that can have multiple fates (i.e., multiple asymptotically stable equilibria) with an inherent bias toward certain fates, resulting in the heterogeneity reported in the literature. For instance, in the later stages of the differentiation protocol for pancreatic islet production, endocrine progenitors can differentiate into β cells (the desired cell fate) but also into α and δ cells.^9^ Similarly, during an early stage of the differentiation protocol for dopaminergic neuron production, stem cells can differentiate into ventral midbrain progenitors (which can subsequently be induced into dopaminergic neurons) as well as into ventral hindbrain progenitors and midbrain-hindbrain boundary cells.^10^ Additionally, in later stages of differentiation for both examples, single-cell analyses have identified more off-target cell types, which are often the result of a previous nonoptimal differentiation step, leading to proliferative cells and various undesired types, such as nonendocrine cells (e.g., duct-like and liver-like cells^11^) and nonmidbrain neurons (e.g., serotonin and GABAergic neurons^10,12^ ).

The yield of the desired cell fate (e.g., β cells or midbrain dopaminergic neurons) can be increased by optimizing these protocols through biomolecular feedback controllers. Rather than titrating the concentrations of molecular regulators within a certain range, suitably designed genetic circuits can bias cell decision-making by dynamically adjusting the concentrations of these regulators within the appropriate range and at the right time, given the correct reference.^13,14^ These circuits can be designed within the same mathematical framework used to study cell decision-making (Supplementary Text). Thus, the challenge is reduced to an engineering problem: how can we design a controller to drive the system from a given initial condition to a desired asymptotically stable equilibrium? Although this problem has been extensively explored in control theory,^15^ the main limitation when translating it to a biological context is the need for mechanistic insight into the regulatory network dynamics that govern cell fate. The stoichiometry of cell fate drivers alone is insufficient for efficient, guided differentiation: both the relative expression levels and the duration of expression must also be considered.^16^ Furthermore, quantifying the impact of introduced exogenous perturbations on the equilibrium landscape remains a challenge. Minimal perturbations may not induce a sufficient bias toward the desired cell fate, whereas larger perturbations may alter the number and the stability properties of the equilibria, potentially making some cell fates inaccessible.^17,18^

In this work, given a gene regulatory network that exhibits multistability, where the asymptotically stable equilibria are associated with different possible cell fates, we propose an adaptive biomolecular feedback controller that introduces a synthetic bias into the decision-making process and thus favors a specific cell fate over the others while minimally altering the corresponding equilibrium value (see the schematic in Figure 2A). Through theoretical analysis, we delineate the requirements needed for generating a biased outcome that favors the desired cell fate and provide design guidelines for the correct operation of the controller. Additionally, we provide computational simulations to address the performance of the controller under parameter variations within the endogenous regulatory network, and extend the application of the controller to the motifs shown in Figure 1B.

**Figure 2:**
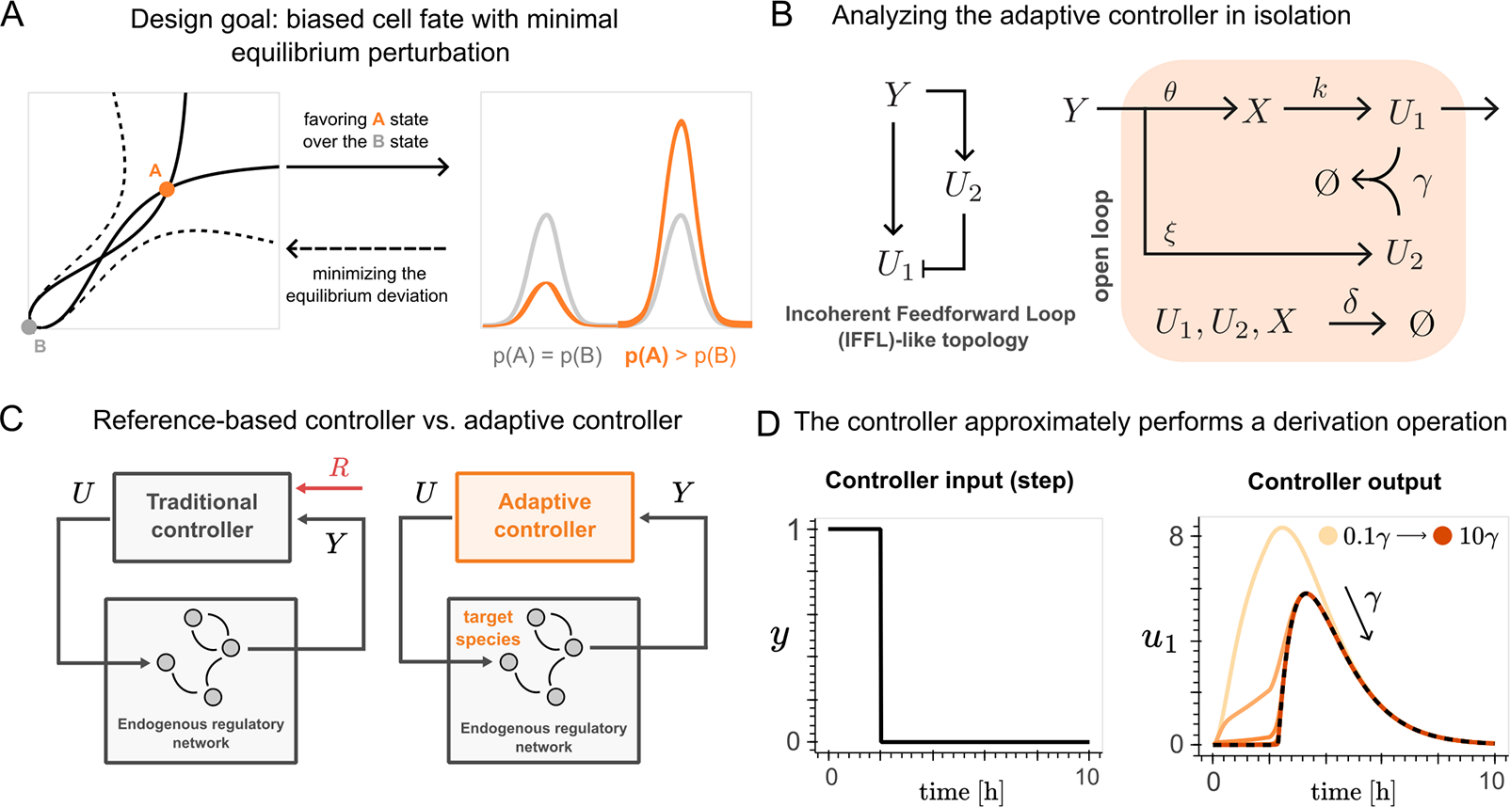
Building an adaptive controller. (A) Our objective is to design a control strategy that favors one asymptotically stable equilibrium (orange) with respect to another (gray), thus leading to a skewed (asymmetric) probability distribution, while minimizing the perturbation to the equilibrium value (i.e., the intersection of the nullclines, depicted as black lines) and avoiding the loss of the multistable behavior (which would occur, for example, if the nullclines became those depicted as dashed lines). (B) The chemical reaction network that implements the adaptive controller has a topology akin to that of an Incoherent Feedforward Loop (IFFL). (C) Feedback controllers generate a control signal *U* based on an error metric estimated by comparing the current output of the process being controlled, *Y*, and a desired set-point value. The feedback controller aims to minimize this error. Typically, controllers require a reference species (*R*) as the set-point, which, however, is often unknown for endogenous networks. In contrast, our proposed adaptive controller can directly estimate the reference from the dynamics of the endogenous regulatory network. (D) If we analyze the controller in isolation, following eq 4, by tuning the adaptive metric $r=\frac{\xi \delta }{k\theta }$ so that *r* = 1, the adaptive controller behaves as a low-pass filter that introduces a delay, followed by a derivative operation. For increasing values of the sequestration rate γ, the time evolution of *u*_1_ obtained by numerically solving eqs 1–3 quickly converges to the approximation in eq 4, represented as black dashed lines in the “Controller output” plot.

## 2 Results

Throughout the manuscript, we denote species with uppercase letters (e.g., *Y*) and their concentrations with the corresponding lowercase letters (e.g., *y*). The time derivative of *y* is denoted by *ẏ* = *dy*/*dt*, while the steady-state concentration of a chemical species *Y* is denoted by $\overset{®}{y}$ = lim_*t*→*∞*_ *y*(*t*).

### 2.1 Adaptive Controller Enables a Biased Cell Fate

#### 2.1.1 Controller and Its Adaptive Nature

We propose a controller to generate a biased cell fate, defined as a probability distribution that is skewed toward the production of the target species, with minimal perturbation to the equilibrium landscape. The controller’s topology, shown in Figure 2B, which builds upon previous work,^19–21^ is similar to an Incoherent Feedforward Loop (IFFL). The controller input *Y* produces at rate constant θ the species *X*, which in turn produces the controller output *U*_1_ at a rate constant *k*. At the same time, *Y* produces at a rate constant ξ the species *U*_2_, which downregulates the expression of *U*_1_ through sequestration at rate constant γ. Species *X*, *U*_1_, and *U*_2_ decay at rate δ. This leads to the following chemical reactions:

$$\begin{array}{llll} Y\overset{\theta }{⇀}Y+X, & Y\overset{\xi }{⇀}Y+U_{2}, & X\overset{k}{⇀}X+U_{1}, & \mathrm{Production},\\ U_{1}+U_{2}\overset{\gamma }{⇀}Ø, & & & \mathrm{Sequestration},\\ X\overset{\delta }{⇀}Ø, & U_{1}\overset{\delta }{⇀}Ø, & U_{2}\overset{\delta }{⇀}Ø, & \mathrm{Decay} \end{array}$$

We use the law of mass action to derive a set of ordinary differential equations (ODEs) that describe the dynamics of the species concentrations in the controller:

$${\dot{u}}_{1}=kx-\delta u_{1}-\gamma u_{1}u_{2}$$ 1

$${\dot{u}}_{2}=\xi y-\delta u_{2}-\gamma u_{1}u_{2}$$ 2

$$\dot{x}=\theta y-\delta x$$ 3

Unlike reference-based feedback controllers, the proposed network does not require a reference species, since it can adaptively estimate the signal to be tracked directly from the dynamics of the process, as illustrated in Figure 2C. To understand its steady-state behavior, we can analyze the controller in isolation (Figure 2B) and derive an approximation of its dynamics in the fast sequestration regime (namely, when γ → *∞*), as detailed in Section 2.1 of the Supporting Information (cf. also Zhang et al.^19^). Then, through a suitable coordinate transformation, we can approximate the dynamics of *U*_1_ as

$$u_{1}(t)\approx \max\left\{0,L^{-1}\left(\frac{k\theta }{(s+\delta {)}^{2}}\left[1-\frac{\xi \delta }{k\theta }-\frac{\xi }{k\theta }s\right]Y(s)\right)\right\}$$ 4

where *Y*(*s*) is the Laplace transform of the input to the controller and $L^{-1}$ denotes the inverse Laplace operator. Let $r=\frac{\xi \delta }{k\theta }$ denote the *adaptive metric*, proposed as a design parameter. Substituting *r* = 1 into eq 4, we observe that the controller asymptotically includes a term resembling a low-pass filter, $\frac{k\theta }{(s+\delta {)}^{2}}$ , and a term resembling a temporal derivative, $\frac{\xi }{k\theta }s$ . The overall result is illustrated in Figure 2D. This behavior was previously demonstrated to function effectively as an adaptive controller.^21^ Applied to a multistable endogenous regulatory network, the controller steers the system trajectories toward a desired fate (i.e., a specific equilibrium) at the price of small perturbations in the expression of the target species. The equilibrium value is minimally perturbed due to the derivative-like behavior of the controller in the fast sequestration regime: the effect of the controller converges to zero asymptotically, as the system reaches the desired equilibrium (proof available in Section 2.2 of the Supporting Information).

#### 2.1.2 Toggle Switch: Model Description

As a first example of a biological multistable system, we consider the canonical *toggle switch*,^22^ a motif consisting of the mutual inhibition of two genes that respectively produce transcriptional repressors *Y*_1_ and *Y*_2_, as shown in Figure 3A1. The chemical reactions are the following,

$$\begin{array}{lll} Ø\overset{\alpha p_{1}}{⇀}Y_{1}, & Ø\overset{\alpha p_{2}}{⇀}Y_{2}, & \mathrm{Production},\\ Y_{1}\overset{\delta }{⇀}Ø, & Y_{2}\overset{\delta }{⇀}Ø, & \mathrm{Decay} \end{array}$$

with $p_{1}=\frac{K^{m}}{K^{m}+y_{2}^{m}}$ and $p_{2}=\frac{K^{m}}{K^{m}+y_{1}^{m}}$ . The corresponding ODE system

$$\begin{array}{cc} {\dot{y}}_{1} & =\alpha \frac{K^{m}}{K^{m}+y_{2}^{m}}-\delta y_{1} \end{array}$$

$$\begin{array}{cc} {\dot{y}}_{2} & =\alpha \frac{K^{m}}{K^{m}+y_{1}^{m}}-\delta y_{2} \end{array}$$

admits two asymptotically stable equilibria to which the system trajectories can converge (Figure 3A2): either *Y*_1_ is maximally expressed and *Y*_2_ is minimally expressed, or vice versa. For zero initial conditions, the trajectories converge to each of the two equilibria with comparable probability, resulting in a bimodal probability distribution where the two peaks have comparable sizes (Figure 3A3).

**Figure 3:**
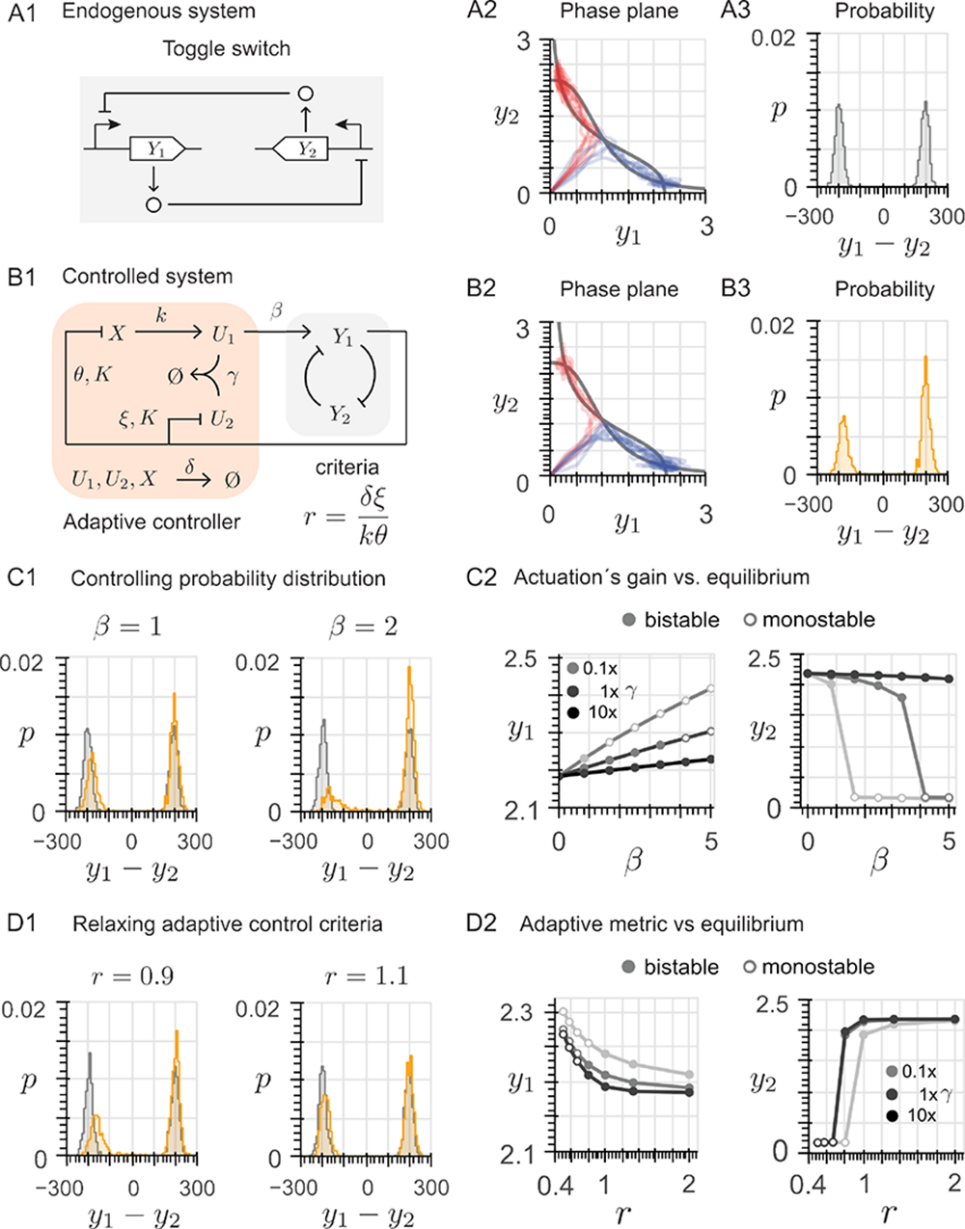
Adaptive controller enables a biased cell fate. (A1) Architecture of the toggle switch, formed by two mutually inhibiting transcription factors *Y*_1_ and *Y*_2_. (A2) For the toggle switch, in the phase plane, the nullclines (gray lines) are shown along with 1000 stochastically perturbed trajectories starting from the origin; each trajectory converges either to the asymptotically stable equilibrium with high expression of *Y*_1_ and low expression of *Y*_2_ (blue trajectories) or to the asymptotically stable equilibrium with high expression of *Y*_2_ and low expression of *Y*_1_ (red trajectories). (A3) The trajectories in A2 converge with comparable probability to either of the two asymptotically stable equilibria, resulting in an unbiased bimodal probability distribution. (B1) Architecture of the controlled system: a feedback loop of the toggle switch and the proposed adaptive controller. (B2) For the controlled system, in the phase plane, the nullclines (gray lines) are shown along with 1000 stochastically perturbed trajectories starting from the origin; more trajectories converge to the asymptotically stable equilibrium with high expression of *Y*_1_ (blue) and less to the asymptotically stable equilibrium with high expression of *Y*_2_ (red). (B3) The controller yields a biased cell fate: the trajectories in B2 converge with higher probability to the equilibrium where the production of *Y*_1_ is favored over that of *Y*_2_. (C1) Increasing the control gain β increases the bias in the cell fate, leading to a larger imbalance in the probability distribution. (C2) Equilibrium values (of *Y*_1_ for the equilibrium associated with high expression of *Y*_1_, and of *Y*_2_ for the equilibrium associated with high expression of *Y*_2_) for increasing control gain β, for different values of the sequestration rate γ (0.1, 1, and 10 times its nominal value). (D1) Effect of a 10% deviation of the adaptive metric $r=\frac{\xi \delta }{k\theta }$ from its nominal value 1: a biased cell fate is still generated at the price of a small alteration of the equilibrium values. (D2) Equilibrium values for varying adaptive metric $r=\frac{\xi \delta }{k\theta }$ , for different values of the sequestration rate γ (0.1, 1, and 10 times its nominal value).

The controlled system, formed by the feedback loop of the endogenous system and our proposed adaptive controller (see schematics in Figure 3B1), is described overall by the chemical reactions:

$$\begin{array}{llll} Ø\overset{\alpha p_{1}}{⇀}Y_{1}, & Ø\overset{\alpha p_{2}}{⇀}Y_{2}, & & \mathrm{Production},\\ Ø\overset{\theta p_{2}}{⇀}X, & Ø\overset{\xi p_{2}}{⇀}U_{2}, & X\overset{k}{⇀}X+U_{1}, & \mathrm{Production},\\ U_{1}\overset{\beta }{⇀}U_{1}+Y_{1}, & & & \mathrm{Adaptive}\;\mathrm{control},\\ Y_{1},Y_{2}\overset{\delta }{⇀}Ø, & X,U_{1},U_{2}\overset{\delta }{⇀}Ø, & & \mathrm{Decay},\\ U_{1}+U_{2}\overset{\gamma }{⇀}Ø, & & & \mathrm{Sequestration} \end{array}$$

We use the law of mass action to derive the ODEs of the controlled system:

$${\dot{y}}_{1}=\alpha \frac{K^{m}}{K^{m}+y_{2}^{m}}-\delta y_{1}+\beta u_{1}$$ 5

$${\dot{y}}_{2}=\alpha \frac{K^{m}}{K^{m}+y_{1}^{m}}-\delta y_{2}$$ 6

$${\dot{u}}_{1}=kx-\delta u_{1}-\gamma u_{1}u_{2}$$ 7

$${\dot{u}}_{2}=\xi \frac{K^{m}}{K^{m}+y_{1}^{m}}-\delta u_{2}-\gamma u_{1}u_{2}$$ 8

$$\dot{x}=\theta \frac{K^{m}}{K^{m}+y_{1}^{m}}-\delta x$$ 9

Equations 5 and 6 model the controlled toggle switch, with the control enforced by the additive term β*u*_1_ in eq 5, while eqs 7–9 describe the controller dynamics.

#### 2.1.3 Dynamic Properties: Biased Cell Fate and Minimum Equilibrium Alteration

To validate the statements derived from the theoretical analysis of eq 4, we solved eqs 5–9 numerically for zero initial conditions. Enforcing the controller preserves the bistability of the toggle switch system, as the nullclines associated with *ẏ*_1_ = 0 and *ẏ*_2_ = 0 still have three intersections, corresponding to two asymptotically stable equilibria (one with low *y*_1_ and high *y*_2_, and the other with high *y*_1_ and low *y*_2_) and one intermediate unstable equilibrium. Thus, the controlled system can also converge to either of the two asymptotically stable equilibria, as shown by the blue and red trajectories in Figure 3B2. However, the symmetry of the probability distribution of the toggle switch in isolation is disrupted: in the controlled system, a higher production of *Y*_1_ is favored with respect to a higher production of *Y*_2_, as shown in Figure 3B3. This property of adjusting the probabilities also holds for nonzero initial conditions. If the system is designed to favor *Y*_1_, it tolerates an initial concentration of *Y*_2_ that is up to a gain-dependent margin higher than that of *Y*_1_. For example, at β = 1, the controlled system maintains the bias only if the initial concentration of *Y*_2_ is less than 0.05% higher than *Y*_1_, while for β = 3, the tolerated difference increases to 15% (see Supplementary Figure 3).

Moreover, a higher bias in the outcome can be generated by increasing the value of the control gain β, as shown in Figure 3C1, at the expense of a larger alteration of the equilibrium value. Above a certain threshold (in this case, β ≥ 4 for the nominal parameters detailed in the Section 4), the system transitions to monostability, as illustrated by the white circles in Figure 3C2. Conversely, increasing the sequestration rate γ reduces deviations from the equilibrium value and prevents the transition to monostability, as indicated by the black circles in Figure 3C2 for a 10-fold increase in γ. However, this adjustment also results in a decreased probability of converging to the equilibrium with higher expression of species *Y*_1_. This highlights the trade-off between maintaining system stability and achieving desired output levels (see Supplementary Figure 4; further details are available in Section 2.1 of the Supporting Information).

As mentioned before, the adaptive behavior of the controller depends on the relationship between its rate constants, which must be such that $r=\frac{\delta \xi }{k\theta }=1$ . As shown in Supplementary Figure 5, if *r* > 1 (which can be obtained, for example, by increasing the degradation rate δ and/or the maximum production rate ξ of *U*_2_), the biasing capacity is lost, while the equilibria remain almost unperturbed. Conversely, if *r* < 1 (which can be obtained, for example, by increasing the maximum production rate θ of *X* and/or the production rate *k* of *U*_1_), the equilibrium landscape is significantly altered, and bistability is eventually lost, but the probability of the biased output increases. Motivated by experimental feasibility, since fine-tuning the kinetic parameters δ, ξ, *k*, and θ so that *r* is exactly 1 is difficult in practice, we provide numerical simulations to assess the effect of an error margin of ±10% (Figure 3D1). Within this range, for a fixed value of the control gain (β = 1), bistability is preserved and the maximum alteration in the equilibrium value is about 8% (cf. Figure 3D2). Further details are available in Section 3 of the Supporting Information. An overview schematic outlining the design principles of the adaptive controller in terms of its tunable parameters (i.e., γ, β, and *r*) is also provided in Supplementary Figure 6.

#### 2.1.4 Adaptive Controller Is Effective in Both Negative and Positive Feedback Architectures

Enforcing the adaptive controller in the toggle switch, as described in Figure 3B1, results in an overall increase in the concentration of *Y*_1_. Therefore, species *X* is subject to an increased inhibition, since *Y*_1_ acts as a repressor; hence, this is a negative feedback control architecture. However, as we now show, the effectiveness of the proposed adaptive controller is independent of the type of feedback provided by the endogenous gene regulatory network. Let us extend our analysis to a positive feedback control architecture. For the toggle switch, this implies that the feedback to the controller originates from the nontarget species *Y*_2_ (see Supplementary Figure 7B1), and the chemical reactions that describe the feedback interaction between the endogenous regulatory network and the input species *X* and *U*_2_ of the adaptive controller become

$$\begin{array}{ccc} Ø\overset{\xi p_{1}}{⇀}U_{2}, & Ø\overset{\theta p_{1}}{⇀}X, & \mathrm{Production} \end{array}$$

with $p_{1}=\frac{K^{m}}{K^{m}+y_{2}^{m}}$ . Consequently, in the ODE system that models the kinetics of the controlled toggle switch, eqs 8 and 9 become

$${\dot{u}}_{2}=\xi \frac{K^{m}}{K^{m}+y_{2}^{m}}-\delta u_{2}-\gamma u_{1}u_{2}$$ 10

$$\dot{x}=\theta \frac{K^{m}}{K^{m}+y_{2}^{m}}-\delta x$$ 11

This alternative control strategy also yields a biased cell fate, favoring the production of species *Y*_1_ (Supplementary Figure 7B2,B3). Analogous to the negative feedback architecture, larger values of the control gain β increase the bias in cell fate, further favoring higher production of the target species *Y*_1_ (Supplementary Figure 7C1), while a larger sequestration rate reduces the alteration in the equilibrium values (Supplementary Figure 7C2). However, the positive feedback architecture requires larger values of the control gain to achieve a probability distribution similar to that obtained with negative feedback. On the other hand, the positive feedback architecture reduces the alteration in the equilibrium values, and exhibits a wider range of values of the adaptive metric *r*, from −20 to +10% of the nominal value 1, for which the performance remains satisfactory: a higher production of *Y*_1_ is still favored with respect to the production of *Y*_2_ (Supplementary Figure 7D1), and the bistability of the toggle switch is preserved (Supplementary Figure 7D2).

In general, once we identify a target species for our control action, as long as the endogenous system exhibits multistability, in the fast sequestration regime (i.e., large γ) and with adaptive metric *r* ≈ 1 (within the aforementioned error margin), the proposed controller can yield a biased cell fate, regardless of the feedback architecture (Supplementary Figure 8A). However, by comparing both architectures, we can observe some specific features of each controller (Supplementary Figure 8B). The negative feedback architecture allows the equilibrium that favors the target species to be reached with higher probability, even with relatively small values of the control gain, but causes a larger alteration of the equilibrium values, even leading to loss of the system’s bistability. Conversely, the positive feedback architecture preserves the system’s bistability for a wider range of values of the control gain and leads to a smaller alteration of the equilibrium values, but the ability to induce a biased cell fate is significantly reduced, for the same control gain value, compared to negative feedback. As a rule of thumb, the negative feedback architecture typically yields 80%–20% distributions, whereas the positive feedback architecture yields 60%–40% distributions.

#### 2.1.5 Changing the Input Species

To assess how the choice of the input species (so far, *U*_1_) affects the controller’s ability to generate a biased cell fate, we changed the input species to *U*_2_. The negative (respectively, positive) feedback control architecture with input *U*_2_ has a steady-state behavior similar to that of the positive (respectively, negative) feedback control architecture with input *U*_1_ (see Section 3 of the Supporting Information). Numerical simulations for both architectures further validate this statement. See Supplementary Figure 9 for the negative feedback architecture and Supplementary Figure 10 for the positive feedback architecture.

### 2.2 Adaptive Controller Exhibits Robustness under Kinetic Mutations

To study the robustness of the controller, we simulated genetic mutations that impact the kinetics of the endogenous regulatory network by affecting the binding between the repressors, *Y*_1_ and *Y*_2_, and their corresponding DNA binding sequences.^23^ For the toggle switch, this amounts to modifying the maximum production rate (α) for each species in the chemical reactions associated with the production of the repressors, which yields to replacing eqs 5 and 6 with

$${\dot{y}}_{1}=\alpha_{1}\frac{K^{m}}{y_{2}^{m}+K^{m}}-\delta y_{1}+\beta u_{1}$$ 12

$${\dot{y}}_{2}=\alpha_{2}\frac{K^{m}}{y_{1}^{m}+K^{m}}-\delta y_{2}$$ 13

while the ODEs that describe the adaptive controller remain similiar to eqs 7–9. We refer to this model as an *unbalanced toggle switch*. For a more systematic analysis, we consider the *ratio* α_2_/α_1_ and study four different cases: a *ratio* larger than 1, due to either increasing α_2_ or decreasing α_1_, and a *ratio* smaller than 1, due to either decreasing α_2_ or increasing α_1_. We consider *robust* a network motif that maintains the same number of equilibria as the original multistable system for a given range of α_2_/α_1_ values and analyze which is the maximum range of parameter variations for which the bistability of the toggle switch is preserved. Since the most straightforward approach to compensate for an imbalance in the maximum expression rate between the two species is the introduction of a positive feedback, the robustness of the adaptive controller will be compared to that of a self-activation motif with chemical reactions

$$\begin{array}{lll} Ø\overset{\alpha_{1}p_{1}}{⇀}Y_{1}, & Ø\overset{\alpha_{2}p_{2}}{⇀}Y_{2}, & \mathrm{Production},\\ Y_{1}\overset{\delta }{⇀}Ø, & Y_{2}\overset{\delta }{⇀}Ø, & \mathrm{Decay},\\ Ø\overset{\alpha_{3}p_{3}}{⇀}Y_{1}, & & \mathrm{Positive}\;\mathrm{feedback} \end{array}$$

with $p_{1}=\frac{K^{m}}{K^{m}+y_{2}^{m}}$ , $p_{2}=\frac{K^{m}}{K^{m}+y_{1}^{m}}$ and $p_{3}=\frac{y_{1}^{m}}{K^{m}+y_{1}^{m}}$ . Under the law of mass action, these reactions correspond to the ODEs

$${\dot{y}}_{1}=\alpha_{1}\frac{K^{m}}{y_{2}^{m}+K^{m}}-\delta y_{1}+\alpha_{3}\frac{y_{1}^{m}}{y_{1}^{m}+K^{m}}$$ 14

$${\dot{y}}_{2}=\alpha_{2}\frac{K^{m}}{y_{1}^{m}+K^{m}}-\delta y_{2}$$ 15

#### 2.2.1 Positive Feedback Architecture Is More Robust to Parameter Variations

Simulations of the behavior of the toggle switch under parameter variations are shown in Figure 4B1–B4. Inherently, this motif is robust for α_2_/α_1_ ∈ [0.8, 1.8] when altering α_2_ (see Figure 4B1,B2), and for α_2_/α_1_ ∈ [0.6, 1.2] when altering α_1_ (see Figure 4B3,B4). For the toggle switch with the self-activation motif, since the positive feedback self-loop is added to *Y*_1_, the effect on the equilibrium is more apparent when α_2_/α_1_ > 1, both due to increasing values of α_2_ (Figure 4C1,C2, red) and due to decreasing values of α_1_ (Figure 4C3,C4, red). Increasing the production of *Y*_1_ compensates for the higher abundance of *Y*_2_, thus preserving bistability for much larger values of the ratio with respect to the toggle switch in isolation (which, in contrast, loses bistability either due to increasing values of α_2_, as shown in Figure 4C1,C2, gray, or to decreasing values of α_1_, as shown in Figure 4C3,C4, gray). Conversely, when α_2_/α_1_ < 1, the higher abundance of *Y*_1_ is amplified by the positive feedback, resulting in a smaller robustness margin with respect to the toggle switch in isolation, eventually leading to loss of bistability when α_2_/α_1_ < 1, both for decreasing values of α_2_ (Figure 4C1,C2, red) and for increasing values of α_1_ (Figure 4C3,C4, red).

**Figure 4:**
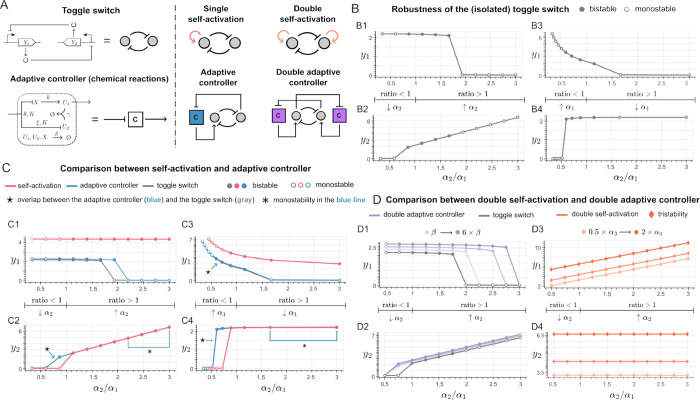
Adaptive controller with the positive feedback architecture is more robust to parameter variations. (A) Schematics of the circuits considered for robustness analysis: the toggle switch with an adaptive controller with the positive feedback architecture (eqs 12 and 13) is compared to the toggle switch with a single self-activation motif (acting on the species that is the control target, eqs 14 and 15, the toggle switch with a double self-activation motif (one on each species, eqs 18 and 19), and the toggle switch with a double adaptive controller with the positive feedback architecture (one acting on each species, eqs 16 and 17). All simulations were performed by evaluating the equations of the nullclines for the considered systems; the same nominal parameters as in Figure 3 were used, except for the maximum production rates α_1_ and α_2_, which were varied accordingly. (B1) and (B2): effect of a varying *ratio* α_2_/α_1_ due to changes in α_2_ on the equilibrium value of species *Y*_1_ in the isolated toggle switch (B1, for the equilibrium associated with high expression of *Y*_1_) and of the equilibrium value of species *Y*_2_ (B2, for the equilibrium associated with high expression of *Y*_2_). (B3) and (B4): same analysis as in B1 and B2, due to changes in α_1_. (C1) to (C4): same analysis as in B1–B4, comparing the toggle switch in isolation (gray), the adaptive controller with positive feedback architecture (blue), and the toggle switch with a single self-activation motif (red). In panels (C2) and (C4), the points highlighted with an * correspond to monostability of the blue line, which is overlapped by the red line. Likewise, in panels (C2), (C3) and (C4), the points highlighted with symbol ★ in the blue lines overlap the gray lines shown in B1 to B4, and exhibit similar stability properties. (D1) and (D2): increasing the gain of the adaptive controller (β) enlarges the range of values of the ratio for which the equilibrium value is preserved and bistability is obtained. For comparison, parts D3 and D4 show the alteration of the equilibrium values for a toggle switch with a double self-activation motif (on each species) when the feedback strength α_3_ is varied.

For the present analysis, we considered the positive feedback architecture of the adaptive controller, since it proved to be more robust than the negative feedback architecture (Supplementary Figure 11). The results are reported in Figure 4C1–C4, in blue. Similarly to the self-activation motif, the adaptive controller also increases the abundance of species *Y*_1_, due to the additive terms in eq 12. However, when considering parameter variations, the adaptive controller can preserve the system’s bistable behavior within a smaller parameter range with respect to the self-activation motif for α_2_/α_1_ > 1, while it allows preservation of the system’s bistable behavior for larger parameter variations when α_2_/α_1_ < 1, as it can be noticed by comparing the blue and red curves in Figure 4C1–C4. Additionally, when comparing the effects of the self-activation motif and the adaptive controller with a fixed control gain β = 1 on the equilibrium values as the ratio of α_2_/α_1_ varies, in all cases we observe negligible alterations (within a 5*%* deviation with respect to the isolated toggle switch); the corresponding effect on the nullclines is shown in Supplementary Figure 12. Conversely, the self-activation motif preserves with mostly negligible alterations the equilibrium value of the species not involved in the positive feedback (*Y*_2_), while the autoinduced species (*Y*_1_) has a significantly different new equilibrium value; this is shown in Figure 4C1,C3 (red), and confirmed by the nullcline computations in Supplementary Figure 13.

When α_2_/α_1_ > 1 due to changes in α_2_ and when α_2_/α_1_ < 1 due to changes in α_1_, the range of values of the ratio for which bistability is preserved can be enlarged by increasing the control gain of the adaptive controller (β), at the expense of a larger alteration of the equilibrium values, which increases with the magnitude of the control gain (see Supplementary Figures 12 and 14A). Similarly, increased robustness when α_2_/α_1_ > 1 due to changes in α_1_ and α_2_/α_1_ < 1 due to changes in α_2_ can be achieved by drastically increasing the sequestration rate γ (10-fold for a noticeable change, as shown in Supplementary Figures 15A and 16). However, as an intrinsic limitation, high values of the sequestration rate, γ, reduce the magnitude of the control action. Overall, the adaptive controller exhibits a comparable robustness margin with respect to the isolated toggle switch (in Figure 4C1–C4, the gray and blue curves are almost always overlapping).

The same conclusions hold for the adaptive controller with a negative feedback configuration under parameter variations, although a narrower range of viable ratios α_2_/α_1_ is observed in this case (see Supplementary Figures 14B and 17; as well as Supplementary Figures 15B and 18).

#### 2.2.2 Additional Adaptive Control Loop Improves Overall Robustness

Either the control gain β or the sequestration rate γ of the adaptive controller can be fine-tuned to increase robustness; however, the required fine-tuning depends on the type of mutation, namely, on which of the system parameters is varied (α_1_ or α_2_). A single controller with a positive feedback architecture can only minimize the alteration of the equilibrium values corresponding to the species not affected by the mutation (e.g., *Y*_1_ for changes in α_2_ and *Y*_2_ for changes in α_1_). Simulating how the nullclines are altered by increasing the positive feedback strength β in the adaptive controller and α_3_ in the self-activation motif (as shown in Supplementary Figures 13 and 17) suggested that the limitations previously discussed can be mitigated by adding to each of the two motifs a second, symmetric feedback loop that targets the other species (see the schematics in Figure 4A). Therefore, we simulated an additional adaptive controller with a double positive feedback architecture, which applies another control input to *Y*_2_, driven by species *X′*, *U*_1_ *′*, and *U*_2_ *′* that share the same production rate constants as *X*, *U*_1_, and *U*_2_. The corresponding chemical reactions are

$$\begin{array}{lll} Ø\overset{\theta p_{2}}{⇀}X^{\prime }, & Ø\overset{\xi p_{2}}{⇀}U_{2}^{\prime }, & \mathrm{Production},\\ X^{\prime }\overset{\delta }{⇀}Ø, & U_{1}^{\prime },U_{2}^{\prime }\overset{\delta }{⇀}Ø, & \mathrm{Decay},\\ U_{1}^{\prime }\overset{\beta^{\prime }}{⇀}U_{1}^{\prime }+Y_{2}, & & \mathrm{Additional}\;\mathrm{adaptive}\;\mathrm{control},\\ U_{1}^{\prime }+U_{2}^{\prime }\overset{\gamma }{⇀}Ø, & & \mathrm{Sequestration} \end{array}$$

with $p_{2}=\frac{K^{m}}{K^{m}+y_{1}^{m}}$ , and the system can be modeled as

$${\dot{y}}_{1}=\alpha_{1}\frac{K^{m}}{y_{2}^{m}+K^{m}}-\delta y_{1}+\beta u_{1}$$ 16

$${\dot{y}}_{2}=\alpha_{2}\frac{K^{m}}{y_{1}^{m}+K^{m}}-\delta y_{2}+\beta^{\prime }u_{1}^{\prime }$$ 17

along with ODEs that describe each controller, which are similar to eqs 7–9, with *u̇*_1_(*t*), *u̇*_2_(*t*), and *ẋ*(*t*) depending on *y*_2_, and *u̇*_1_ *′*(*t*), *u̇*_2_ *′*(*t*), and *ẋ* *′*(*t*) depending on *y*_1_. See Section 6 of the Supporting Information for further details.

Analogously, we can model the addition of another self-activation motif in the toggle switch, acting on the nontarget species, with the same maximum expression rate α_3_, described by the chemical reactions

$$\begin{array}{lll} Ø\overset{\alpha_{1}p_{1}}{⇀}Y_{1}, & Ø\overset{\alpha_{2}p_{2}}{⇀}Y_{2}, & \mathrm{Production},\\ Y_{1}\overset{\delta }{⇀}Ø, & Y_{2}\overset{\delta }{⇀}Ø, & \mathrm{Decay},\\ Ø\overset{\alpha_{3}p_{3}}{⇀}Y_{1}, & Ø\overset{\alpha_{3}p_{4}}{⇀}Y_{2}, & \mathrm{Positive}\;\mathrm{feedback} \end{array}$$

with $p_{1}=\frac{K^{m}}{K^{m}+y_{2}^{m}}$ , $p_{2}=\frac{K^{m}}{K^{m}+y_{1}^{m}}$ , $p_{3}=\frac{y_{1}^{m}}{K^{m}+y_{1}^{m}}$ , $p_{4}=\frac{y_{2}^{m}}{y_{2}^{m}+K^{m}}$ . Then, the dynamics of the toggle switch with a self-activation motif on each species is described by the ODEs

$${\dot{y}}_{1}=\alpha_{1}\frac{K^{m}}{y_{2}^{m}+K^{m}}-\delta y_{1}+\alpha_{3}\frac{y_{1}^{m}}{y_{1}^{m}+K^{m}}$$ 18

$${\dot{y}}_{2}=\alpha_{2}\frac{K^{m}}{y_{1}^{m}+K^{m}}-\delta y_{2}+\alpha_{3}\frac{y_{2}^{m}}{y_{2}^{m}+K^{m}}$$ 19

Comparative simulations reveal that the toggle switch with self-activation motifs on both species is so robust that the behavior is multistable, independent of the ratio α_2_/α_1_ (see Figure 4D3,D4). However, this strategy is only effective to preserve bistability when α_3_ ≪ α_1,2_; with higher values of α_3_, new intermediate equilibria appear, leading to a loss of bistability in favor of tristability (see the curves with diamonds in Figure 4D3,D4, as well as Supplementary Figure 19). On the other hand, the addition of another adaptive controller with a positive feedback architecture enables robust performance for an extended α_2_/α_1_ range, regardless of the parameter that is being varied. As observed before, increasing the gain of both controllers (assuming β = β′ for simplicity) can maintain a bistable behavior for an increased range of values of α_2_/α_1_: the configuration with two controllers in a fast sequestration (large γ) and high gain (large β) regime exhibits bistability for values of α_2_/α_1_ from 0.7 to 2.7, with an alteration of the equilibrium value of at most 25*%*, and without the generation of new, intermediate equilibria (see Figure 4D1,D2, as well as the systematic characterization in Supplementary Figure 20, and the nullcline analysis in Supplementary Figure 21).

### 2.3 Adaptive Controller: General Applicability

To outline general design principles for the application of the adaptive controller to any type of regulation and to multistable networks with more than two asymptotically stable equilibria, we consider as case studies the mutual activation motif and the toggle switch with self-activation.

#### 2.3.1 Applying the Adaptive Control Principle to a Different System

The mutual activation (double activation) motif in Figure 5A1 has a similar architecture to the toggle switch (double repression): in both cases, we have an overall positive loop, which is known to be associated with multistability.^24–26^ In view of the bistable behavior arising from the mutual activation between species *Y*_1_ and *Y*_2_, two asymptotically stable equilibria emerge: either both species are highly expressed, or both are not expressed (Figure 5A2). Favoring high expression for both species is trivial since any increase in the abundance of one species is amplified by the mutual activation loop. We modified the controller to favor a lack of expression for both species by enforcing the chemical reactions

$$\begin{array}{lll} Ø\overset{\alpha p_{4}}{⇀}Y_{1}, & Ø\overset{\alpha p_{3}}{⇀}Y_{2}, & \mathrm{Production},\\ Y_{1}\overset{\delta }{⇀}Ø, & Y_{2}\overset{\delta }{⇀}Ø, & \mathrm{Decay},\\ Ø\overset{\theta p_{3}}{⇀}X, & Ø\overset{\xi p_{3}}{⇀}U_{2}, & \mathrm{Production},\\ X\overset{\delta }{⇀}Ø, & U_{1},U_{2}\overset{\delta }{⇀}Ø, & \mathrm{Decay},\\ Y_{1}\overset{\beta p_{5}}{⇀}Ø & U_{1}+U_{2}\overset{\gamma }{⇀}Ø, & \mathrm{Inhibition}/\mathrm{Sequestration} \end{array}$$

with $p_{3}=\frac{y_{1}^{m}}{y_{1}^{m}+K^{m}}$ , $p_{4}=\frac{y_{2}^{m}}{y_{2}^{m}+K^{m}}$ and $p_{5}=\frac{u_{1}}{y_{1}+K}$ (which amounts to adopting a Michaelis–Menten approximation to model *U*_1_-mediated degradation of species *Y*_1_).^27^ The first two reactions are associated with the mutual activation motif; all the others are associated with the adaptive controller in a negative feedback architecture. With mass action kinetics, the chemical reactions lead to the ODEs

$${\dot{y}}_{1}=\alpha \frac{y_{2}^{m}}{y_{2}^{m}+K^{m}}-\delta y_{1}-\beta u_{1}\frac{y_{1}}{y_{1}+K}$$ 20

$${\dot{y}}_{2}=\alpha \frac{y_{1}^{m}}{y_{1}^{m}+K^{m}}-\delta y_{2}$$ 21

$${\dot{u}}_{1}=kx-\delta u_{1}-\gamma u_{1}u_{2}$$ 22

$${\dot{u}}_{2}=\xi \frac{y_{1}^{m}}{K^{m}+y_{1}^{m}}-\delta u_{2}-\gamma u_{1}u_{2}$$ 23

$$\dot{x}=\theta \frac{y_{1}^{m}}{K^{m}+y_{1}^{m}}-\delta x$$ 24

**Figure 5:**
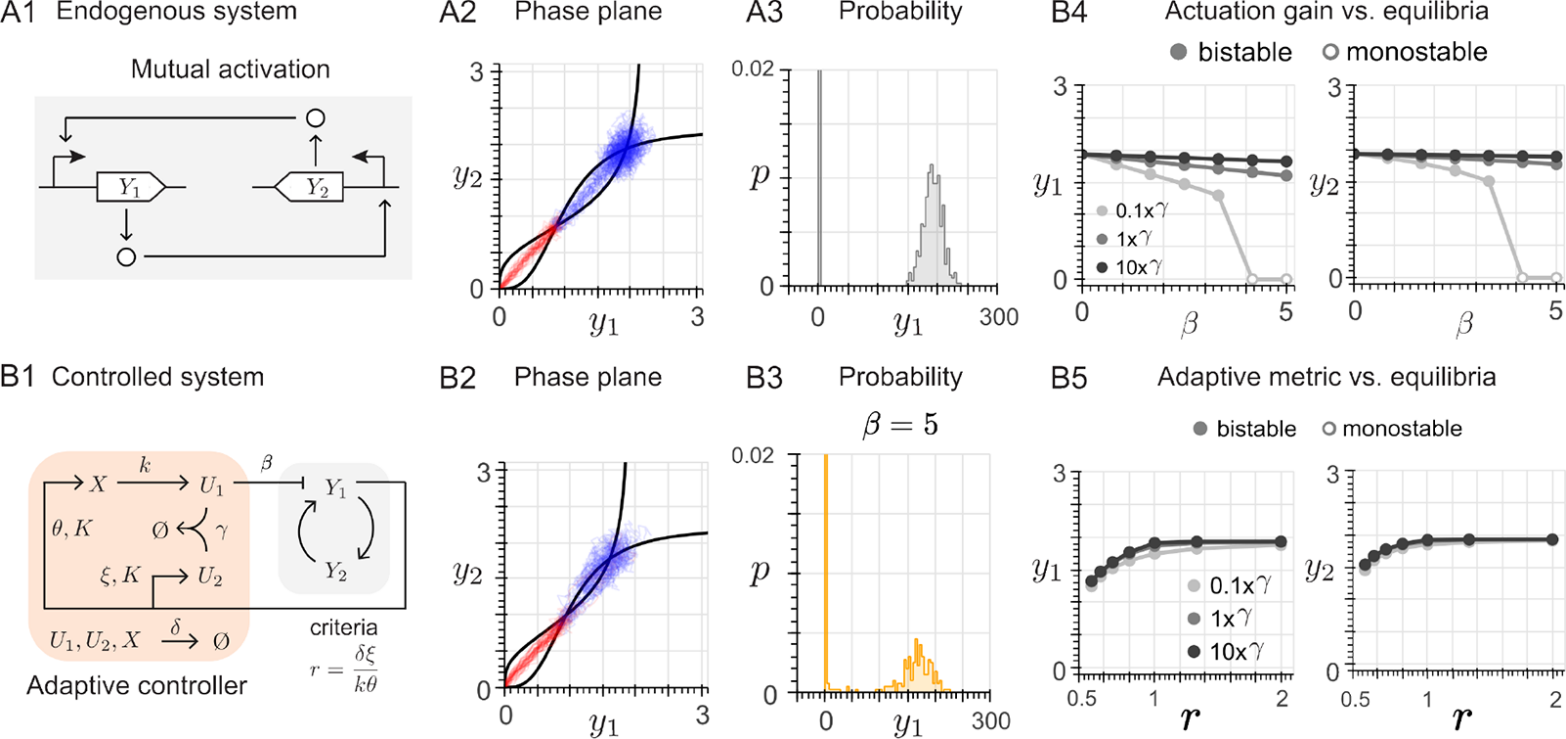
Applying the adaptive controller principle to a different system. (A1) Mutual activation motif, composed of two mutually activating transcription factors *Y*_1_ and *Y*_2_. (A2) For the mutual activation motif, nullclines (black lines) and trajectories in the phase plane, converging either to the asymptotically stable equilibrium with high expression of both *Y*_1_ and *Y*_2_ (blue) or to the equilibrium with zero expression (red). (A3) The trajectories in A2 yield an asymmetric probability distribution for each species. The probability distribution for *Y*_2_ is the same as that for *Y*_1_. (B1) Architecture of the proposed adaptive controller with an inhibiting effect through the species *U*_1_. (B2) For the controlled system, nullclines (black lines) and trajectories in the phase plane. (B3) Generation of a biased cell fate, which favors the equilibrium with no expression: the probability of converging to the equilibrium with high expression of *Y*_1_ significantly decreases. The same result holds for *Y*_2_ (not shown here). (B4) Characterization of the effect of increasing inhibition strength β on the high equilibrium values of *Y*_1_ and *Y*_2_, for different values of the sequestration rate γ. (B5) Characterization of the effect of varying adaptive metric $r=\frac{\xi \delta }{k\theta }$ on the high equilibrium values of *Y*_1_ and *Y*_2_, for different values of the sequestration rate γ.

The resulting architecture of the adaptive controller is depicted in Figure 5B1. As in the toggle switch case, the bistability of the mutual activation motif is preserved when enforcing the controller (compare with Figure 5A2,B2). Hence, the system trajectories can still converge to either of the two asymptotically stable equilibria (high production of both species vs no production of both species). However, the resulting bimodal distribution is not symmetric already in the original mutual activation system (Figure 5A3). Enforcing the controller favors convergence to the zero equilibrium, at which both species are not produced (Figure 5B3). The same properties hold for the positive feedback architecture, as well as when *U*_2_ is selected as the input species (see Supplementary Figure 22A1–A4,B1–B4). In general, high control gains (β > 3) were needed to achieve a noticeable level of inhibition, which resulted in a moderate alteration of the equilibrium value associated with the high expression of both species (Figure 5B4). By adding another adaptive controller that targets *Y*_2_, a similar level of inhibition can be achieved with lower gain values, resulting in a smaller alteration of the equilibrium value (Supplementary Figure 22C1–C4).

#### 2.3.2 Applying the Adaptive Control Principle to Multistable Systems

To gain insight into the application of the adaptive controller to control the convergence to an intermediate equilibrium, we evaluated the steady-state behavior of the toggle switch with a self-activation motif in both species, the architecture of which is depicted in Figure 6A1. In this case, in light of the high density of trajectories converging to the intermediate equilibrium (at which both species are equally abundant) and the corresponding peak in the probability distribution (Figure 6A2,A3, respectively), we sought to design a strategy to reduce the probability of the system to converge to the intermediate equilibrium. To this aim, we implement an inhibition strategy using an adaptive controller for each species (Figure 6B1), based on the results obtained from the analysis of the mutual activation motif. Considering the same chemical reactions used to describe the kinetics of both the toggle switch with a double self-activation motif and the inhibitory strategy, the same equations as eqs 18 and 19 can be considered, with α_1_ = α_2_ = α_3_ = α for simplicity, and a control input acting on each species, resulting in the ODEs

$${\dot{y}}_{1}=\alpha \frac{K^{m}}{y_{2}^{m}+K^{m}}+\alpha \frac{y_{1}^{m}}{y_{1}^{m}+K^{m}}-\delta y_{1}-\beta u_{1}\frac{y_{1}}{y_{1}+K}$$ 25

$${\dot{y}}_{2}=\alpha \frac{K^{m}}{y_{1}^{m}+K^{m}}+\alpha \frac{y_{2}^{m}}{y_{2}^{m}+K^{m}}-\delta y_{2}-\beta^{\prime }u_{1}^{\prime }\frac{y_{2}}{y_{2}+K}$$ 26

where the controller equations have a similar form as eqs 7–9. With a small alteration of the equilibrium value (Figure 6B2), the proposed control strategy reduces the probability of converging to the intermediate equilibrium and induces a higher concentration of either *Y*_1_ or *Y*_2_ (Figure 6B3).

**Figure 6:**
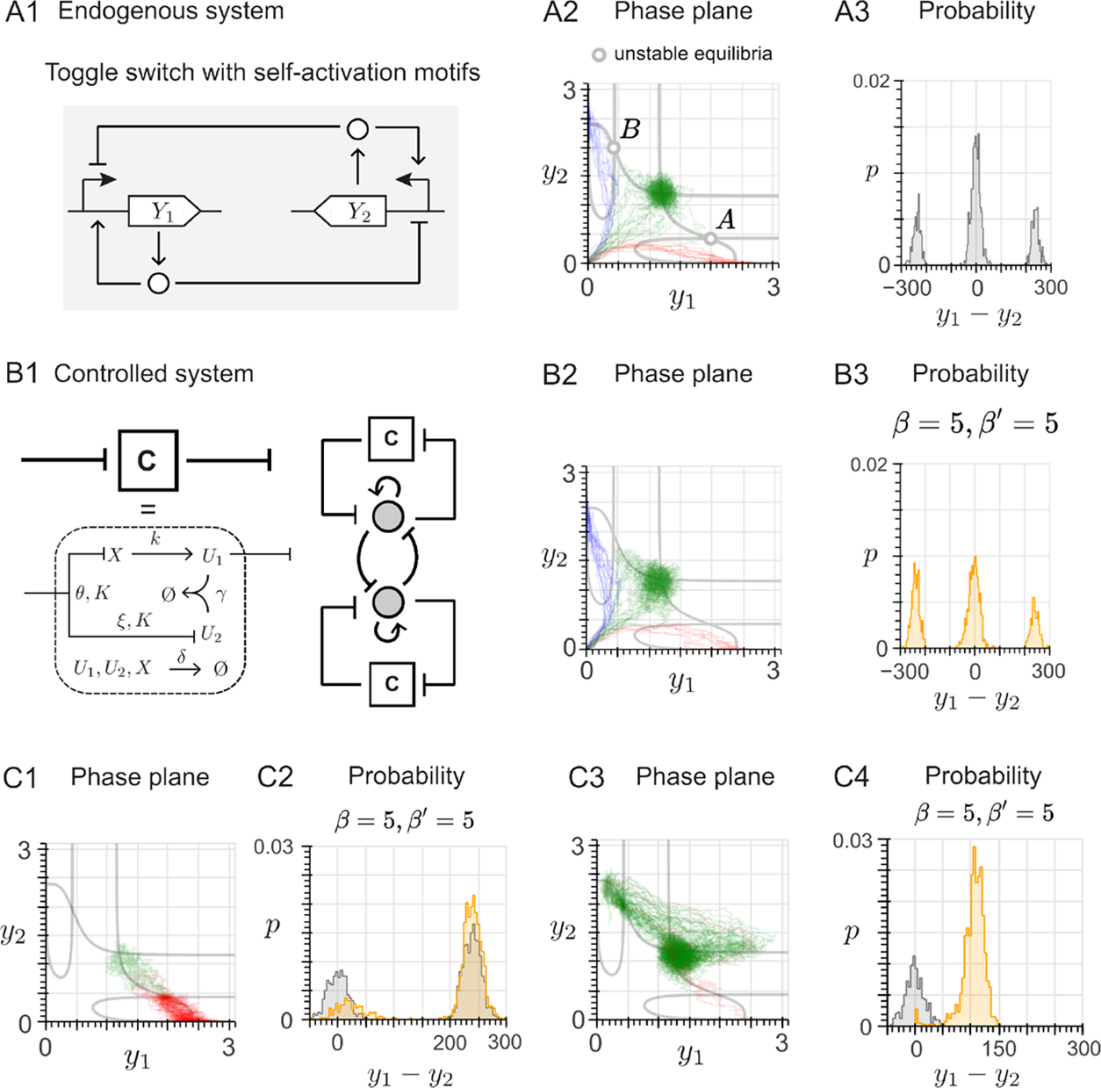
Controlling an intermediate equilibrium. (A1) Architecture of the toggle switch with a self-activation loop acting on each species. (A2) For the toggle switch with a double self-activation, nullclines (gray lines) and trajectories in the phase plane. The system admits three asymptotically stable equilibria: high expression of *Y*_2_ and low expression of *Y*_1_ (attracting the trajectories in blue), low expression of *Y*_2_ and high expression of *Y*_1_ (attracting the trajectories in red), and an intermediate state with the same level of expression for both *Y*_1_ and *Y*_2_ (attracting the trajectories in green). It also admits two unstable equilibria, corresponding to those denoted as “A” and “B”. (A3) The trajectories in A2 yield a multimodal probability distribution, where convergence to the intermediate equilibrium is favored. (B1) Architecture of the proposed control strategy to reduce the probability of convergence to the intermediate equilibrium of (A1): two adaptive controllers with the negative feedback architecture are implemented. (B2) For the system with both controllers, nullclines and trajectories in the phase plane. (B3) Generation of the desired biased cell fate: the probability of converging to the intermediate equilibrium is decreased. (C1) For the system with both controllers, nullclines and trajectories in the phase plane obtained by considering eqs 27 and 28 and setting the initial conditions close to the unstable equilibrium “A” in panel A2. (C2) The trajectories in panel C1 generate the desired cell fate, favoring convergence to the equilibrium with high expression of *Y*_1_ and low expression of *Y*_2_ (red trajectories in C1, generating the orange distribution) over convergence to the intermediate equilibrium with comparable expression of *Y*_1_ and *Y*_2_ (green trajectories in C1, generating the gray distribution). (C3) Same as panel C1, but setting the initial conditions close to the unstable equilibrium “B” in panel A2. (C4) The adaptive control strategy fails to generate the desired cell fate for the conditions of panel C2: most trajectories still converge to the intermediate equilibrium (green trajectories in C3, generating the orange distribution) and not to the equilibrium with high expression of *Y*_1_ and low expression of *Y*_2_ (red trajectories in C3, generating the gray distribution).

So far, we have considered zero initial conditions for the species *Y*_1_ and *Y*_2_, as this enables the trajectories to converge to the equilibria with equal probability. Extending the analysis to other initial conditions (mainly in the neighborhood of unstable equilibria) allows us to define the working limits of the adaptive controller. Particularly, to illustrate the conditions in which the controller fails to generate a biased cell fate, we set as our goal to design a strategy to favor the high production of the *Y*_1_ species, associated with the equilibrium corresponding to high expression of *Y*_1_ and low expression of *Y*_2_ (to which the red trajectories converge in Figure 6A2,B2). As for the control strategy, we consider a network similar to that shown in Figure 6B1, but with a positive (activating) effect over the *Y*_1_ species rather than a negative (inhibitory) one. Hence, the controller simultaneously increases the production of the target species *Y*_1_ and decreases the production of the nontarget species *Y*_2_. Considering the same chemical reactions used to describe the previous example and including a positive action as the one applied for the toggle switch with a single controller with the negative feedback architecture, we can modify eqs 25 and 26, resulting in the following ODEs:

$${\dot{y}}_{1}=\alpha \frac{K^{m}}{y_{2}^{m}+K^{m}}+\alpha \frac{y_{1}^{m}}{y_{1}^{m}+K^{m}}-\delta y_{1}+\beta u_{1}$$ 27

$${\dot{y}}_{2}=\alpha \frac{K^{m}}{y_{1}^{m}+K^{m}}+\alpha \frac{y_{2}^{m}}{y_{2}^{m}+K^{m}}-\delta y_{2}-\beta^{\prime }u_{1}^{\prime }\frac{y_{2}}{y_{2}+K}$$ 28

where the controller equations have a similar form as eqs 7–9. As shown in Figure 6C1, if we set the initial conditions close to the unstable equilibrium named as “A” in the phase plane (see Figure 6A2), and apply the controller, we notice that a biased cell fate is achievable, favoring the convergence to the equilibrium associated with the high expression of *Y*_1_ and the low expression of *Y*_2_, rather than to the intermediate equilibrium (Figure 6C2). However, following the same control goal, if we set the initial conditions close to the unstable equilibrium named “B” in the phase plane (see Figure 6A2), the control strategy fails to achieve the desired cell fate (as apparent from Figure 6C3,C4), even for increasing values of the control gains either activating the expression of the target species *Y*_1_ (increasing β) or inhibiting the expression of the nontarget species *Y*_2_ (increasing β′), resulting only in a moderate deviation to the intermediate equilibria, as observed in the shift of the probability distribution shown in Figure 6C4. See Supplementary Figure 23 for more details.

## 3 Discussion

Synthetic biology offers promising tools to engineer cell fate and improve the production of cell types that are phenotypically and functionally equivalent to their in vivo counterparts, thus enabling the development of novel cell-based therapeutics and disease models.^4^ In this work, we propose a synthetic gene circuit based on an Incoherent Feedforward Loop motif and molecular sequestration to generate an output proportional to an approximated discrete temporal derivative of its input. By enabling the controller to act on a target species involved in a decision-making process within a multistable gene regulatory network, we achieve a tunable synthetic bias in cell fate with minimal alteration of the equilibrium value.

Throughout this work, we outlined the parametric conditions that enable a biased cell fate through theoretical analysis, resulting in two principal design requirements: the circuit needs to operate in a fast sequestration regime (large γ, ideally γ → *∞*) and satisfy the ideal adaptive metric *r* = ξδ/*k*θ = 1. When both conditions are met, our computational simulations demonstrate the generation of a synthetic bias, which can be further tuned through the control gain parameter β. A limitation of the proposed design is the need for fine-tuning of the kinetic parameters to achieve the ideal adaptive metric during experimental implementation. Although biological differentiators have not yet been implemented experimentally, previous theoretical work regarding approximated differentiators and derivative motifs for biomolecular PID controllers already suggests synthetic implementations.^28–33^ Further advances in RNA technology and protein engineering offer even more choices to achieve the desired tunability.^34,35^ Overall, to assess the robustness with respect to variations in key parameters and metrics, we provide complementary simulations, which show that a relatively wide variation of the adaptive metric (±10% with respect to the equilibrium without the controller) does not significantly alter the control performance.

Furthermore, we consider a wide portfolio of design architectures built by varying both the feedback species, resulting in negative and positive feedback architectures, and the input species to the endogenous network, either *U*_1_ or *U*_2_. Our simulations demonstrate that all the considered architectures can generate a synthetic bias with minimum alteration of the equilibrium value, and validate that the effectiveness of the adaptive controller is independent of the (positive or negative) feedback architecture and of the input to the controlled species. For control gains below a threshold value (β = 1 for the toggle switch case study), the performance is essentially the same, regardless of the architecture. On the other hand, for control gains above that threshold, the behavior of the controller is architecture-specific. Negative feedback more strongly biases cell fate, but alters the equilibrium value more significantly. Hence, this architecture is well suited for increasing the yield of differentiation protocols and maximizing a desired population, such as β-like cells during stem-cell-derived pancreatic islet manufacturing,^5^ or midbrain dopaminergic neurons,^7,8^ while minimizing the number of cells of undesired, off-target types.^9,10^ On the other hand, positive feedback alters the equilibrium values less, but also induces less bias in the cell fate, making it better suited for subpopulation tuning; for example, balancing the cell ratio between β and α within an islet-like cluster,^9^ or adjusting the proportion between parenchymal and supporting cells, often associated with increased functionality and maturation.^36,37^

As endogenous regulatory networks grow in complexity (for instance, the number of genes involved in the decision-making process increases), quantifying kinetic parameters for further mathematical modeling is harder. Hence, it is fundamental to address parameter uncertainty, due to parameter variations or even fluctuations over time.^38^ When the system is subject to parameter variations (in particular, in the case of an unbalanced toggle switch introduced in Section 2.2), we show that increasing the value of the control gain in the positive feedback architecture confers robustness to changes in the kinetic parameters. However, only the equilibrium values corresponding to the controlled species can be maintained. An additional controller can be implemented to extend the range of parameter variations in which the multistable behavior (bistable in our case study) is maintained, but even for higher gain values, we can only ensure minimal alteration of the equilibrium value for the species unaffected by the kinetic mutation.

Finally, we outline general principles for applying the controller to different endogenous networks, demonstrating that both positive (activating) and negative (inhibiting) effects can be enforced over the endogenous network. Overall, by considering the mutual inhibition (Section 2.1.3) and mutual activation (Section 2.3.1) examples, we show that a synthetic bias can be generated by either favoring the production of the desired species (positive, activation-based control) or decreasing the production of the other species (negative, repression-based control). Moreover, we illustrate a preliminary strategy for translating the adaptive controller to higher-order multistable systems, considering the toggle switch with a self-activating motif on each species as an example. Given the tristable behavior of this network, control over the probability of convergence to the intermediate equilibrium, associated with the equal expression of both species, was achieved by considering two adaptive controllers, each inhibiting one species. Based on these results, we conjecture that a multistable system where intermediate equilibrium values are present may need one adaptive controller per species. We also identified conditions under which the adaptive controller failed to generate a biased cell fate. Within this context, considering that the transfer function shown in eq 4 resembles a proportional-derivative (PD) controller,^19^ future work will aim at implementing a strategy to generate an adaptive mechanism for the metric *r* = ξδ/*k*θ itself, yielding a two-step control strategy where the proportional component bypasses the basin of attraction of the intermediate equilibrium (e.g., based on the high-gain feedback controller proposed in ref 39), and eventually vanishes (*r* = 1), so that only the derivative component (described in this work) is left active.

A few studies have previously addressed cell fate control through the experimental implementation of synthetic gene circuits. Haellman et al.^40^ optimized a vanillic-acid responsive induction system to enhance control over gene expression in human stem cells: the dose–response curve was fine-tuned into a switch, and applied to successfully commit progenitor cells to a pancreatic fate by regulating the expression of the TGIF2 and HHEX genes, but with a low yield (<20% of the total population). In a more recent, proof-of-concept study, Prochazka et al.^41^ designed a Boolean classifier able to discriminate between stem cells and differentiated cells, and actuate accordingly. The circuit was used to adjust the proportion between the three primordial germlines by controlling the expression of the endogenous morphogen BMP4; and through the action of the circuit in human pluripotent stem cells, the relative cell composition can switch from a predominant ectoderm-like fate to relatively balanced subpopulations, including endoderm-like and mesoderm-like fates, although each represent <30 and ∼10% of the total population, respectively. Both strategies implement an open-loop control, and hence require a predefined input (the optimal concentration of TGIF2 for commitment into a pancreatic fate^40^ and the microRNA sequence that encodes the desired BMP4 secretion level^41^), whose knowledge is hard to achieve, also because concentrations vary in time. Galloway et al.^14^ addressed this drawback by adding a feedback loop through a promoter responsive to the signaling pathway (MAPK) that controls the decision between mating and nonmating fate in yeast by combining positive and negative actions. Upon optimization, the controller was able to increase or decrease mating efficiency up to 70%. This last strategy resembles traditional controller design (see Figure 2A), but is still constrained by the knowledge of the dynamics of the reference species, which can be empirically determined, but could be difficult to extrapolate to higher organisms.^14^ In this context, a key technical advantage of the proposed adaptive controller lies in the fact that it requires only partial knowledge of the species involved in the decision-making event, and it can thus exploit current computational algorithms that are not yet able to map the complete signaling pathways regulating cell fate but provide enough information for design.^42–44^

An interesting avenue for future work on the control of multistable systems involves exploring switching between cell states (e.g., in Figure 2A2, switching from the equilibrium attracting red trajectories to the equilibrium attracting blue trajectories) with minimum alteration of the equilibrium value, often referred to as “reprogramming”, with potential applications to stem-cell-based therapeutics^45^ and regenerative medicine.^46^ Similarly, novel engineered circuits that exhibit multistability, such as the MultiFate,^47^ can be implemented for in vitro characterization, and further improvements to the present work can address the limitations when targeting intermediate equilibria in high-dimensional networks. Ultimately, our understanding of the nonlocal behavior and transient response of the adaptive controller can be enhanced by adopting more advanced mathematical methods to address the nonlinearities of the proposed chemical networks.

## 4 Methods

For deterministic simulations, the ODE models described in this work were integrated using Python’s *scipy* package with the parameters listed in Table 1. A comprehensive list of the models that were simulated, along with their corresponding nullcline equations, is available in Section 6 of the Supporting Information. These were used to generate phase plane plots and systematically characterize deviations from the equilibrium, as detailed in each figure’s captions. When the nullclines did not have closed-form algebraic expressions, numerical methods were used to approximate them by using the *scipy* and *numpy* packages. For stochastic simulations, the Gillespie algorithm was implemented through the *biocircuits* package. Probability distributions were computed as histograms generated from over 1000 trajectories for *t* = 200 h, with a bin number of 8.

**Table 1:** Nominal Parameters for the Endogenous Regulatory Networks and Adaptive Controller^a^

parameter	units	description	value
Toggle Switch and Mutual Activation Motif			
α	μM/h	production	2.2
*K*	μM	Hill activation constant	1
*m*	dimensionless	cooperativity	3
δ	/h	decay	1
Toggle Switch with Self-Activation			
α	μM/h	production	1.2
*K*	μM	Hill activation constant	0.5
*m*	dimensionless	cooperativity	4
δ	/h	decay	1
Adaptive Controller			
ξ	μM/h	production	1
θ	μM/h	production	1
*k*	/h	production	1
β	/h	control gain	1
γ	/μM/h	sequestration rate	100

a Kinetic rates were obtained from previous work.^19,48,49^

## Supplementary Material



## Data Availability

All data presented in this article were generated exclusively using computational models, which can be found in our Github repository (https://github.com/frank-britto/engineering_cell_fate), along with the original code for generating both the figures in the main text and the Supplementary Figures.

## Notes

Section 1: interpretation of the epigenetic Waddington landscape from a dynamical systems perspective; Sections 2 and 3: theoretical analysis complemented by numerical simulations to further demonstrate the dynamics properties of the controller (i.e., minimal perturbation to the equilibrium at the high sequestration regime, and guarantee of the dynamics properties for an error margin of the adaptive metric *r*); Section 4: numerical simulations of the dynamics properties of the adaptive controller in the positive feedback configuration, and when switching the actuation species from *U*_1_ to *U*_2_; Section 5: numerical simulations of robustness for the negative feedback configuration, and complementary nullclines analysis; and Section 6: comprehensive list of chemical reactions, ordinary differential equations, and nullclines equations for each closed-loop system analyzed throughout the manuscript (PDF)
