# Supplementary material for: Engineering Cell Fate with Adaptive Feedback Control
Source: ACS Synth Biol. 2025 Jul 23;14(8):3163–76. doi: 10.1021/acssynbio.5c00299 (PMC12362612; doi:10.1021/acssynbio.5c00299)
Supplement: Supplementary file 1 [file sb5c00299_si_001.pdf]

Engineering cell fate with adaptive feedback control (Supplementary Material)

Frank Britto Bisso[1], Giulia Giordano[2], and Christian Cuba Samaniego[1,*]

[1]Computational Biology Department, Carnegie Mellon University, Pittsburgh, PA 15213, USA
[2]Department of Industrial Engineering, University of Trento, Trento, 38123, Italy
[*]Corresponding author, ccubasam@andrew.cmu.edu

Contents

1 Supplementary Text **S3**

2 Analysis of the adaptive controller **S4**
2.1 Approximated dynamics in the fast sequestration regime S4
2.2 The adaptive controller barely perturbs the equilibrium in the fast sequestration regime . S5
2.2.1 Non-dimensionalization of the ODEs S5
2.2.2 Boundedness analysis S6
2.2.3 Controller-induced perturbation of the equilibrium in the fast sequestration regime S6

3 Design guidelines for the adaptive controller **S7**
3.1 The adaptive controller barely perturbs the equilibrium S7
3.2 Analyzing deviations from the adaptive metric S8

4 Robustness analysis under kinetic mutations **S18**

5 Extending the applications of the adaptive controller **S30**

6 Models **S33**
6.1 Toggle switch and adaptive controller with negative feedback and U_1 actuation S33
6.2 Toggle switch and adaptive controller with positive feedback and U_1 actuation S34
6.3 Toggle switch and adaptive controller with positive feedback and U_2 actuation S35
6.4 Toggle switch and adaptive controller with negative feedback and U_2 actuation S36
6.5 Mutual activation system and adaptive controller S37
6.5.1 Single inhibiting controller in a negative feedback with actuation through U_1 species S38
6.5.2 Single inhibiting controller in a positive feedback with actuation through U_1 species S39
6.5.3 Single inhibiting controller in a negative feedback with actuation through U_2 species S40
6.5.4 Double inhibiting controller with actuation through U_1 and U_1' species S41
6.6 Toggle switch with self-activation and adaptive controller with negative feedback S42
6.6.1 Double inhibiting controller with actuation through U_1 and U_1' species S42
6.6.2 Inhibition-activation pair S43

1 Supplementary Text

Decision-making and cell fate: insights from dynamical systems theory

Dynamical systems theory provides a powerful mathematical framework to formalize Waddington's idea of the epigenetic landscape. Within this framework, the state of a cell—characterized by its unique gene expression profile—can be visualized in a coordinate system known as the *phase plane*, where each axis represents the expression level of a specific gene that defines the cell's state (Fig. 1–C in the Main Text). Changes in gene expression are represented as the movement of a point along a trajectory in this plane. However, these trajectories are not arbitrary: they are constrained by the underlying gene regulatory networks [1, 2, 3]. Some of these networks exhibit a property called *multistability*, which allows for the emergence of multiple asymptotically stable equilibria (also known as attractors), towards which the trajectories eventually converge. In the context of Waddington's landscape, each "valley" corresponds to one of these equilibria, representing a distinct cell fate. Bistable and tristable systems are among the most studied multistable networks in synthetic biology (Fig. 1–B in the Main Text). In this context, cell decision-making can be conceptualized as the process by which a cell reaches one of these equilibria.

Recent advancements in algorithms for estimating dynamic trajectories from single-cell RNA sequencing snapshots have significantly improved our ability to identify key genes responsible for specific cell fates within high-dimensional gene regulatory networks [4, 5]. However, the intricate nature of these networks often makes it difficult to understand the mechanisms by which gene interactions lead to particular cell fates [6]. As an alternative, a bottom-up approach examines simpler gene regulatory motifs, such as self-activation, mutual activation, and mutual inhibition (Fig. 1–B in the Main Text), and builds up more elaborate dynamics from these fundamental building blocks. These motifs have been extensively studied both theoretically and experimentally, primarily in the context of differentiation [7, 8, 9]; and although initially proposed for studying developmental processes, this framework can be applied to virtually any biological process involving multistability, including cell cycle entry [10], quiescence [11], macrophage activation [12], and yeast mating [13].

Cell decision-making is inherently a single-cell event and thus exhibits stochasticity [14]. Consequently, for a given initial cellular state (i.e., a point in the *phase plane*), there is a probability distribution corresponding to each potential cell fate. During development, these distributions are often skewed, favoring one cell fate over others. While multistability can explain the emergence of diverse cell types from a single progenitor, the varying proportions of cell types within a tissue reflect this inherent bias [15, 16, 17]. In our work, we designed a synthetic gene circuit to replicate this effect by introducing a "synthetic bias" in cell decision-making, as illustrated in Fig. 1–A in the Main Text.

2 Analysis of the adaptive controller

The results illustrated in the main text to demonstrate the dynamic properties of the proposed adaptive controller in a closed-loop system can be further complemented with theoretical analysis and numerical simulations. In this section, we will address the following properties:

1. In the high sequestration regime ($\gamma \to \infty$), the controller introduces minimum perturbation to the equilibrium values, evaluated through the analysis of the nullclines corresponding to the toggle switch (mutual inhibition).
2. The dynamic properties guaranteed when the adaptive metric is $r = 1$ are maintained for bounded variations in the value of r.

2.1 Approximated dynamics in the fast sequestration regime

In previous work, we analyzed the combination of an Incoherent FeedForward Loop (IFFL) and a negative feedback network (NF) to generate a band-pass filter with tunable cut-off frequencies [18]. We can follow the same analytical approach to evaluate the dynamics of the proposed controller. Starting with the chemical reactions that describe the open-loop system, for an arbitrary input species Y,

$Y \xrightarrow{\theta} Y + X$	$X \xrightarrow{\delta} \emptyset$	Production/Decay
$Y \xrightarrow{\xi} Y + U_2$	$U_2 \xrightarrow{\delta} \emptyset$	Production/Decay
$X \xrightarrow{k} X + U_1$	$U_1 \xrightarrow{\delta} \emptyset$	Production/Decay
$U_1 + U_2 \xrightarrow{\gamma} C$	$C \xrightarrow{\delta} \emptyset$	Sequestration/Decay

Here, we also consider species C, the complex formed by species U_1 and U_2 as a result of the sequestration reaction, which also decays at rate δ. Under the law of mass action, we can model these chemical reactions using Ordinary Differential Equations (ODEs). We adopt the same coordinate transformation described in [18], so that $u_1^*(t) = u_1(t) + c(t)$ and $u_2^*(t) = u_2(t) + c(t)$. In this way, we can avoid the nonlinearities and describe the controller's dynamics as follows:

$$\begin{aligned}
\dot{u_1^*} &= kx(t) - \delta u_1^*(t) \\
\dot{u_2^*} &= \xi y(t) - \delta u_2^*(t) \\
\dot{x} &= \theta y(t) - \delta x(t) \\
\frac{1}{\gamma}\dot{c} &= [u_1^*(t) - c(t)][u_2^*(t) - c(t)] - \frac{\delta}{\gamma} c(t)
\end{aligned}$$

Applying the timescale separation argument in [19], it can be shown that, in the fast sequestration regime ($\gamma \to \infty$), the asymptotic value for $u_1(t)$ can be computed as

$$\lim_{\gamma \to \infty} \bar{u}_1 = \max\{0, \bar{u}_1^* - \bar{u}_2^*\}. \tag{1}$$

where $\bar{u}_1^*$ and $\bar{u}_2^*$ are the steady-state values of $u_1^*(t)$ and $u_2^*(t)$, respectively. Furthermore, since the dynamics of $u_1^*(t)$, $u_2^*(t)$ and $x(t)$ are described by linear ODEs, we can take the Laplace transform of the corresponding differential equations and write

$$X(s) = \frac{\theta}{s+\delta} Y(s) \tag{2}$$

$$U_1^*(s) - U_2^*(s) = \frac{k}{s+\delta} X(s) - \frac{\xi}{s+\delta} Y(s) \tag{3}$$

Replacing Eq. (2) in Eq. (3) yields the following expression for the difference between $U_1^*(s)$ and $U_2^*(s)$ in the Laplace domain:

$$U_1^*(s) - U_2^*(s) = \frac{k\theta - \xi\delta - \xi s}{s^2 + 2\delta s + \delta^2} = \frac{k\theta}{(s+\delta)^2}\left[1 - \frac{\xi\delta}{k\theta} - \frac{\xi}{k\theta}s\right]Y(s). \tag{4}$$

Finally, by taking the inverse Laplace transform of Eq. (4) and considering Eq. (1), we obtain the approximated expression shown in the main text, valid in the fast sequestration regime ($\gamma \to \infty$):

$$u_1(t) \approx \max\left\{0, \mathcal{L}^{-1}\left(\frac{k\theta}{(s+\delta)^2}\left[1 - \frac{\xi\delta}{k\theta} - \frac{\xi}{k\theta}s\right]Y(s)\right)\right\}. \tag{5}$$

By defining the adaptive metric as in the main text, $r = \xi\delta/k\theta$, and setting $r = 1$, we obtain:

$$u_1(t) \approx \max\left\{0, \mathcal{L}^{-1}\left(\underbrace{\frac{k\theta}{(s+\delta)^2}}_{\text{Low-pass filter}} \quad \underbrace{\left[-\frac{\xi}{k\theta}s\right]}_{\text{Derivative}} Y(s)\right)\right\} \tag{6}$$

In the same way, applying the timescale separation argument in [19], it can be shown that, in the fast sequestration regime ($\gamma \to \infty$), the asymptotic value for $u_2(t)$ can be computed as

$$\lim_{\gamma\to\infty} \bar{u}_2 = \max\{0, \bar{u}_2^* - \bar{u}_1^*\} \tag{7}$$

We can follow similar steps to analyze the linear ODEs that describe $u_1^*(t)$, $u_2^*(t)$ and $x(t)$ to obtain the following approximation,

$$u_2(t) \approx \max\left\{0, \mathcal{L}^{-1}\left(\frac{k\theta}{(s+\delta)^2}\left[\frac{\xi\delta}{k\theta} - 1 + \frac{\xi}{k\theta}s\right]Y(s)\right)\right\} \tag{8}$$

2.2 The adaptive controller barely perturbs the equilibrium in the fast sequestration regime

2.2.1 Non-dimensionalization of the ODEs

We define new variables to obtain a non-dimensionalized representation of the closed-loop system, considering the toggle switch case study. Defining $\tau = \delta t, \hat{y}_1 = \frac{y_1}{K}, \hat{y}_2 = \frac{y_2}{K}, \hat{u}_1 = \frac{u_1}{K}, \hat{u}_2 = \frac{u_2}{K}, \hat{x} = \frac{x}{K}$ along with parameters $\hat{\alpha} = \frac{\alpha}{\delta K}, \hat{\beta} = \frac{\beta}{\delta}, \hat{k} = \frac{k}{\delta}, \hat{\gamma} = \frac{\gamma K}{\delta}, \hat{\xi} = \frac{\xi}{\delta K}, \hat{\theta} = \frac{\theta}{\delta K}$ yields the ODEs:

$$\frac{d}{d\tau}\hat{y}_1 = \hat{\alpha}\frac{1}{1+\hat{y}_2^m} - \hat{y}_1 + \hat{\beta}\hat{u}_1 \tag{9}$$

$$\frac{d}{d\tau}\hat{y}_2 = \hat{\alpha}\frac{1}{1+\hat{y}_1^m} - \hat{y}_2 \tag{10}$$

$$\frac{d}{d\tau}\hat{u}_1 = \hat{k}\hat{x} - \hat{u}_1 - \hat{\gamma}\hat{u}_1\hat{u}_2 \tag{11}$$

$$\frac{d}{d\tau}\hat{u}_2 = \hat{\xi}\frac{1}{1+\hat{y}_1^m} - \hat{u}_2 - \hat{\gamma}\hat{u}_1\hat{u}_2 \tag{12}$$

$$\frac{d}{d\tau}\hat{x} = \hat{\theta}\frac{1}{1+\hat{y}_1^m} - \hat{x} \tag{13}$$

The approximated dynamics of U_1, described in Eq. (5), can be also retrieved considering the non-dimensionalized ODEs, by redefining the adaptive metric as $r = \frac{\hat{\xi}}{\hat{k}\hat{\theta}}$.

2.2.2 Boundedness analysis

Following a similar approach as in [18], we can guarantee boundedness of all the state variables, since

$$\frac{d}{d\tau}\hat{x} < \hat{\theta} - \hat{x} \tag{14}$$
$$\frac{d}{d\tau}\hat{u}_2 < \hat{\xi} - \hat{u}_2 \tag{15}$$
$$\frac{d}{d\tau}\hat{u}_1 < \hat{k}\hat{\theta} - \hat{u}_1 \tag{16}$$
$$\frac{d}{d\tau}\hat{y}_2 < \hat{\alpha} - \hat{y}_2 \tag{17}$$
$$\frac{d}{d\tau}\hat{y}_1 < \hat{\alpha} - \hat{y}_1 + \hat{\beta}\hat{k}\hat{\theta} \tag{18}$$

2.2.3 Controller-induced perturbation of the equilibrium in the fast sequestration regime

The nullclines for species Y_1 and Y_2 are obtained by setting $d\hat{y}_1/d\tau = 0$ and $d\hat{y}_2/d\tau = 0$ in Eqs. (9) and (10), which yields

$$\begin{aligned}\hat{y}_1 &= \hat{\alpha}\frac{1}{1+\hat{y}_2^m} + \hat{\beta}\bar{u}_1 \\ \hat{y}_2 &= \hat{\alpha}\frac{1}{1+\hat{y}_1^m}\end{aligned}$$

When $\hat{\beta} = 0$, the system is known to admit either one or three equilibria [20]. Considering $m = 2$, we can compute the equilibria by finding the real roots of the following 5-th order polynomial, obtained by substituting the expression of $\hat{y}_2$ in the expression of $\hat{y}_1$:

$$P_p(\hat{y}_1) = a_5\hat{y}_1^5 + a_4\hat{y}_1^4 + a_3\hat{y}_1^3 + a_2\hat{y}_1^2 + a_1\hat{y}_1 + a_0 = 0$$

where $a_5 = 1, a_4 = -\hat{\alpha}$, $a_3 = 2$, $a_2 = -2\hat{\alpha}$, $a_1 = 1 + \hat{\alpha}^2$ and $a_0 = -\hat{\alpha}$. Then, to analyze the effect of the adaptive controller's actuation (through $\hat{u}_1$) in the equilibrium of the toggle switch, we can study how the coefficients of the aforementioned polynomial change when $\hat{\beta} \neq 0$. Hence,

$$P(\hat{y}_1) = P_p(\hat{y}_1) \underbrace{-\hat{\beta}\hat{u}_1(\hat{y}_1^4 + 2\hat{y}_1^2 + 1 + \hat{\alpha}^2)}_{\text{actuation}} = 0 \tag{19}$$

We can further find $\hat{u}_1$ as a function of $\hat{y}_1$ by setting $d\hat{u}_1/d\tau = 0$, $d\hat{u}_2/d\tau = 0$ and $d\hat{x}/d\tau = 0$:

$$\begin{aligned}\hat{u}_2 &= \frac{k\hat{x} - \hat{u}_1}{\hat{\gamma}\hat{u}_1} = \frac{\hat{\xi}/(1+\hat{y}_1^m)}{\hat{\gamma}\hat{u}_1 + 1} \\ \hat{x} &= \hat{\theta}\frac{1}{1+\hat{y}_1^m}\end{aligned}$$

Let $f_1 = \hat{k}\hat{x}$ and $f_2 = \hat{\xi}\frac{1}{1+\hat{y}_1^m}$. Then, the nullcline of $\hat{u}_1$ is the second order polynomial

$$\hat{u}_1^2 + \hat{u}_1\Big(f_2 - f_1 + \frac{1}{\hat{\gamma}}\Big) - \frac{f_1}{\hat{\gamma}} = 0 \tag{20}$$

Since concentrations cannot be negative, the only feasible solution is

$$\hat{u}_1 = \frac{1}{2}\left(f_1 - f_2 - \frac{1}{\hat{\gamma}} + \sqrt{\left(f_2 - f_1 + \frac{1}{\hat{\gamma}}\right)^2 + \frac{4f_1}{\hat{\gamma}}}\right) \tag{21}$$

Now, recalling the adaptive metric $r = \hat{\xi}/\hat{k}\hat{\theta}$, by setting $f_1 - f_2 = \hat{k}\theta(1-r)\hat{y}^*$, where $y^* = \frac{1}{1+\hat{y}_1^m}$, we can rewrite Eq. (21) as follows:

$$\hat{u}_1(r) = \frac{1}{2}\left(y^*(1-r)\hat{k}\hat{\theta} - \frac{1}{\hat{\gamma}} + \sqrt{\left(-y^*(1-r)\hat{k}\hat{\theta} + \frac{1}{\hat{\gamma}}\right)^2 + \frac{4f_1}{\hat{\gamma}}}\right) \tag{22}$$

Then, the asymptotic value of $\hat{u}_1$ when the adaptive metric is $r = 1$ is

$$\hat{u}_1(1) = \frac{1}{2}\left(-\frac{1}{\hat{\gamma}} + \sqrt{\frac{1}{\hat{\gamma}^2} + \frac{4f_1}{\hat{\gamma}}}\right) \leq \sqrt{\frac{f_1}{\hat{\gamma}}} \tag{23}$$

From Eq. (14), we notice that

$$\hat{u}_1(1) \leq \sqrt{\frac{f_1}{\hat{\gamma}}} \leq \sqrt{\frac{\hat{k}\hat{\theta}}{\hat{\gamma}}} \tag{24}$$

We can substitute this last upper bound for $\hat{u}_1$ in the polynomial describing the nullcline of $\hat{y}_1$ in Eq. (19), and obtain

$$P(\hat{y}_1) = P_p(\hat{y}_1) - \hat{\beta}\sqrt{\frac{\hat{k}\hat{\theta}}{\hat{\gamma}}}(\hat{y}_1^4 + 2\hat{y}_1^2 + 1 + \hat{\alpha}^2)$$

In the high sequestration regime ($\hat{\gamma} \to \infty$), the asymptotic expression of $P(\hat{y}_1)$ approaches that of $P_p(\hat{y}_1)$. In fact, the effect of the controller actuation on the coefficients of the polynomial vanishes: $\hat{\beta}\sqrt{\frac{\hat{k}\hat{\theta}}{\hat{\gamma}}} \to 0$ as $\hat{\gamma} \to \infty$. Hence, the roots of $P(\hat{y}_1)$ will be close to those of $P_p(\hat{y}_1)$, since the perturbation due to the controller is negligible and the roots of a polynomial are continuous functions of its coefficients.

3 Design guidelines for the adaptive controller

3.1 The adaptive controller barely perturbs the equilibrium

We can verify numerically the statement that the adaptive controller induces a negligible perturbation of the equilibrium values by comparing the equilibria of the closed-loop system with the adaptive controller applied to the toggle switch and the equilibria of the open-loop toggle switch system. First, we compute the dynamics of $\hat{u}_1(\tau)$ by solving Eqs. (9) to (13). For increasing values of the sequestration rate $\hat{\gamma}$, Fig. S1-A shows how the steady-state value of the input $\hat{u}_1$ generated by the adaptive controller approaches zero. Nevertheless, since setting the sequestration rate to a very high value is difficult experimentally, then $\hat{u}_1(\tau)$ will converge to a small, non-zero value that slightly perturbs the equilibrium. Fig. S1-B characterizes the normalized error between the equilibrium of the toggle switch in isolation

(open-loop) and in the presence of the adaptive controller (closed loop); we show the normalized error Δy_1 as a function of the sequestration rate without loss of generality in the analysis. The same results can be obtained for species Y_2. For each value, we notice how the actuation gain ($\hat{\beta}$) determines the maximum possible perturbation to the equilibrium value. We can also determine empirically the stability properties of the closed-loop system as a function of both $\hat{\gamma}$ and $\hat{\beta}$, as shown in Fig. S1-C. From a designer perspective, once the high sequestration regime is achieved by choosing $\hat{\gamma}$ large enough, the actuation gain $\hat{\beta}$ needs to be kept below a ($\hat{\gamma}$-dependent) threshold value so as to preserve bistability. Fig. S4 further illustrates the effect of varying the sequestration rate and the control gain on both the equilibrium landscape in the phase plane and the probability distribution of the asymptotic values of the state.

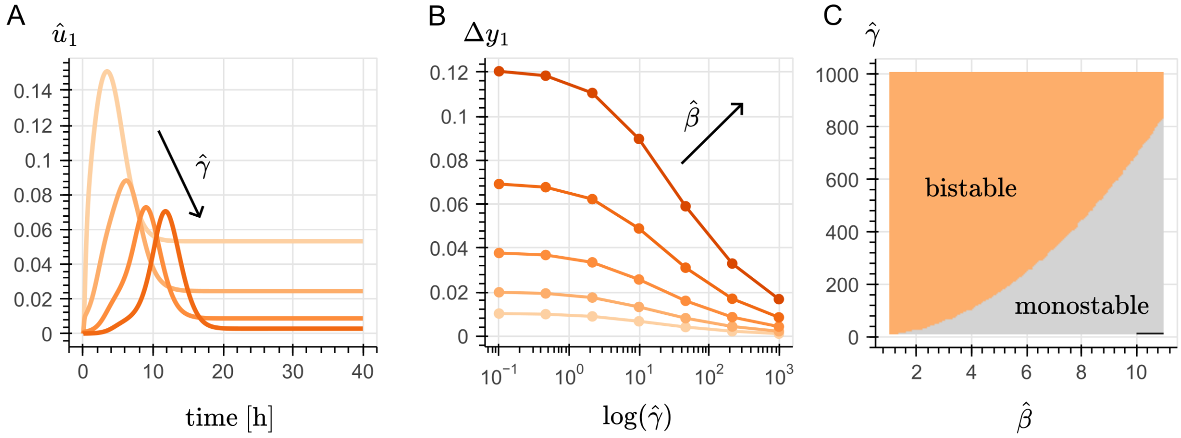

Figure S1: The adaptive controller barely perturbs the equilibrium. *(A) In the closed-loop system described by Eqs.* (9)-(13)*, by increasing the value of the sequestration rate $\hat{\gamma}$ (whose magnitude is denoted by progressively darker orange colors), the steady-state value of $\hat{u}_1(\tau)$ approaches zero. (B) The normalized error $\Delta y_1 = \frac{\bar{y}_1^{OL} - \bar{y}_1^{CL}}{\bar{y}_1^{OL}}$ as a function of $\hat{\gamma}$ for different values of $\hat{\beta}$, where $\bar{y}_1^{OL}$ is the equilibrium value of y_1, at the equilibrium associated with the high expression of Y_1 and low expression of Y_2, for the toggle switch in isolation (open loop, Eqs.* (9) *and* (10) *with $\hat{\beta} = 0$), while $\bar{y}_1^{CL}$ is equilibrium value of y_1 at the same equilibrium, for the closed-loop toggle switch with the adaptive controller (Eqs.* (9)-(13) *with $\hat{\beta} \neq 0$). (C) The relationship between $\hat{\beta}$ and $\hat{\gamma}$ suggested by panel B is explored in terms of stability properties, by numerically determining the number of intersections between the nullclines, derived by equating Eqs.* (9)-(13) *to zero. The system is denoted as* bistable *when the nullclines have three intersections, corresponding to one unstable equilibrium and two asymptotically stable equilibria; while the system is denoted as* monostable *if the nullclines have a single intersection, corresponding to one asymptotically stable equilibrium.*

3.2 Analyzing deviations from the adaptive metric

With an approach similar to that of the analysis presented in Section 1.1, we can determine an expression for the approximated dynamics of the adaptive controller in isolation, as the one provided in Eq. (6), using the non-dimensionalized model described in Eqs. (9)-(13). Considering the adaptive metric $r = \hat{\xi}/\hat{k}\hat{\theta}$, we can rewrite the expression as follows:

$$\hat{u}_1(\tau) \approx \max\left\{0, \mathcal{L}^{-1}\left(\frac{\hat{k}\hat{\theta}}{(s+1)^2}\left[1 - r - \frac{\hat{\xi}}{\hat{k}\hat{\theta}}s\right]Y(s)\right)\right\} \tag{25}$$

For a fixed gain value $\hat{\beta}$, the capacity of the adaptive controller to generate a biased output depends

on the value of actuation species $\hat{U}_1$. According to Eq. (25), for $r = 1$, that is true when the gradient is negative, since there is a negative sign before to the s operator. A deviation from the metric introduces an offset, as depicted in Fig. S2-A. When $r > 1$, a larger gradient is needed for $u_1(t) > 0$. Thus, for the same range of values for the input $Y(s)$, $u_1(t)$ takes smaller values with respect to when $r = 1$, and hence the probability of a biased output decreases. Inversely, when $r < 1$, a smaller gradient is needed for $u_1(t) > 0$. Similarly, for the same input, $u_1(t)$ takes larger values with respect to when $r = 1$, and hence the probability of a biased output increases.

As a reference, Fig. S2-B shows the input/output map of the adaptive controller in isolation, satisfying the ideal adaptive metric ($r = 1$). For comparison, Fig. S2-C replicates the same input/output map in the fast sequestration regime, varying the r metric within a $\pm 20\%$ margin of its ideal value. As mentioned before, taking the magnitude of $\hat{u}_1(\tau)$ as a baseline, $r < 1$ yields an increased magnitude, while $r > 1$ yields a reduced magnitude; see Fig. S5 for a thorough analysis of the effects of deviations from the ideal value of the adaptive metric r.

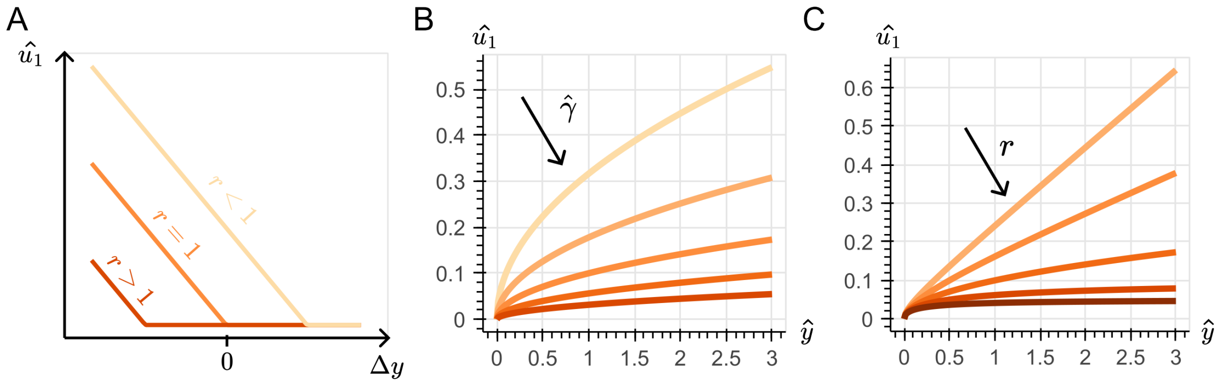

Figure S2: Deviations from the ideal adaptive metric. *(A) With the ideal adaptive metric ($r = 1$), the adaptive controller responds to negative gradients ($-\frac{\hat{\xi}}{\hat{k}\hat{\theta}}s$). Any deviations from $r = 1$ result in an offset. (B) Input/output map of the controller in isolation, highlighting the magnitude of $\hat{u}_1$ as a function of $\hat{y}$ for different values of the sequestration rate $\hat{\gamma}$. (C) Input/output map of the controller for a fixed, high sequestration rate ($\hat{\gamma} = 1000$), and varying $\hat{\xi}$ within a 20% of its nominal value, resulting in r varying between 0.8 and 1.2 times its nominal value 1.*

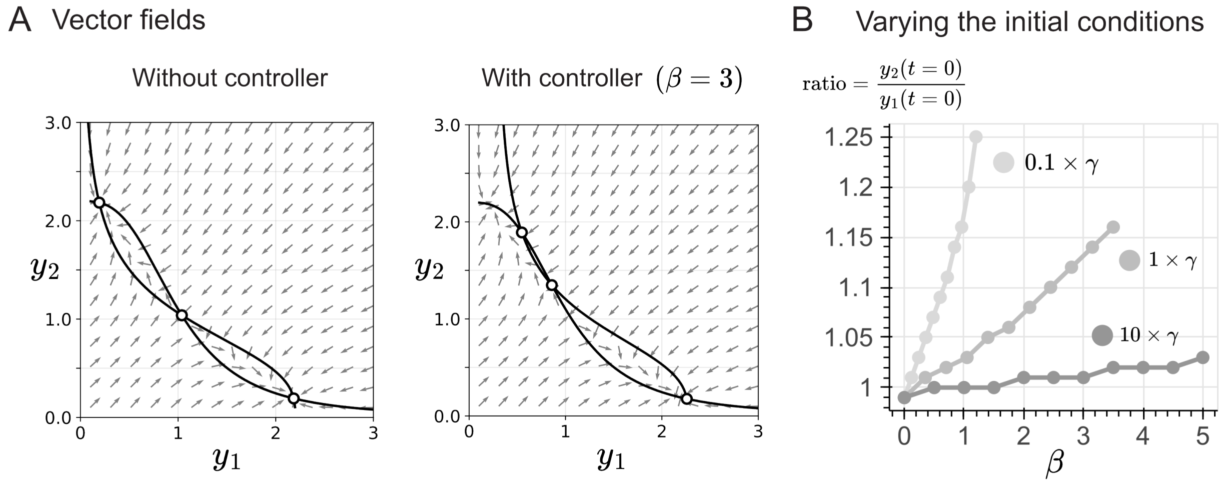

Figure S3: The biased cell fate depends on the initial conditions. *(A) The vector fields for the isolated toggle switch (left) and the controlled system in a high-gain regime (right) reveal two basins of attraction, corresponding to the asymptotically stable equilibria: one with high expression of Y_1 and low expression of Y_2 (bottom-right, white circle), and another with low expression of Y_1 and high expression of Y_2 (upper-left, white circle). To determine whether the controller alters the phase portrait, we computed the divergence of the vector field, defined as the sum of the partial derivatives of each ODE with respect to its corresponding variable. We found no difference between the isolated toggle switch and controlled system. (B) Starting from an initial concentration of Y_1 fixed at 1 μM, we computed the maximum initial concentration of Y_2, denoted $y_2(t=0)$, that maintains both a biased cell fate and the bistability of the system for increasing values of the controller gain (β). This threshold is expressed as a ratio between $y_2(t=0)$ and $y_1(t=0)$, shown on the vertical axis. We repeated this computation for three values of the sequestration rate: 0.1, 1, and 10 times the nominal value of $\gamma = 100$.*

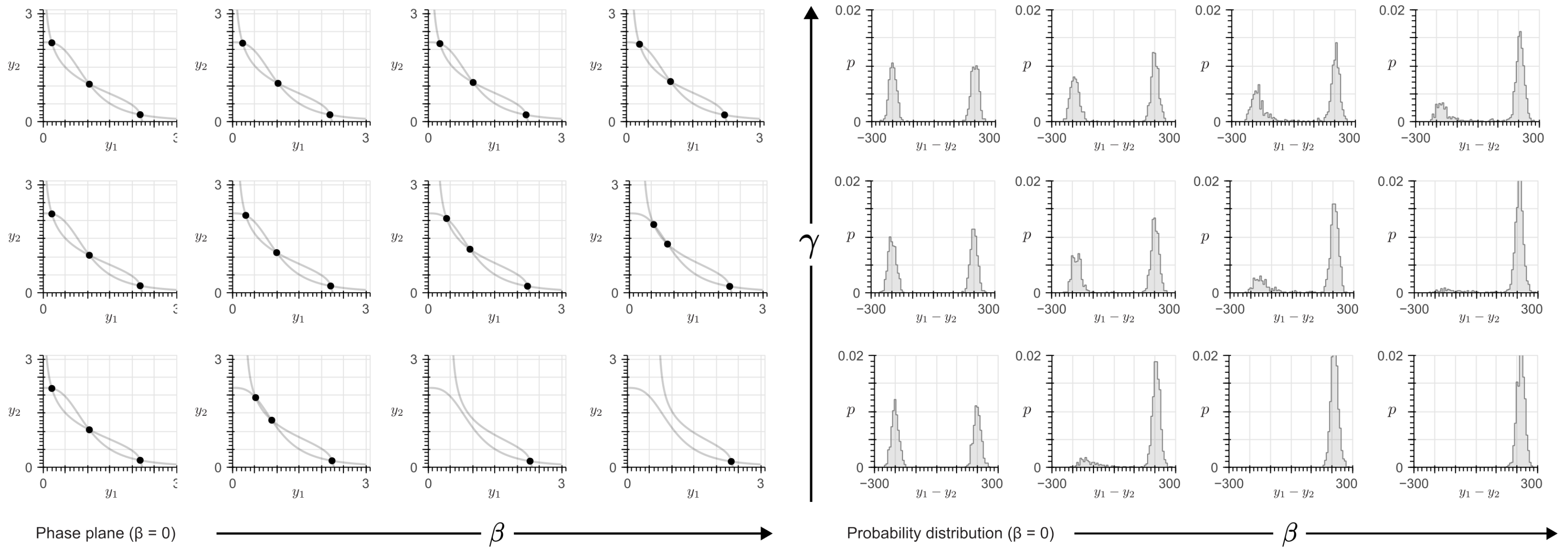

Figure S4: Trade-off between the sequestration rate (γ) and the control gain (β)*.* *From left to right, increasing the value of the control gain β corresponds to a larger deviation to the location of the equilibria, as well as an increase in the probability distribution favoring the production of species Y_1. A sufficiently high gain value ($\beta \geq 4$ for this specific example) results in a* monostable *system (i.e, a single asymptotically stable equilibrium, and a corresponding single "peak" in the probability distribution). From bottom to top, increasing the value of the sequestration rate γ decreases the perturbation to the equilibrium, but yields a more symmetric, bimodal probability distribution, akin to the one corresponding to the isolated toggle switch. Simulations were performed by numerically solving Eqs.* (26)-(30) *for the nominal values indicated in Table 1. From left to right, β corresponds to 1, 2 and 3 times the nominal value $\beta = 1$. From bottom to top, γ corresponds to 0.1, $1X$ and 10 times the nominal value $\gamma = 100$.*

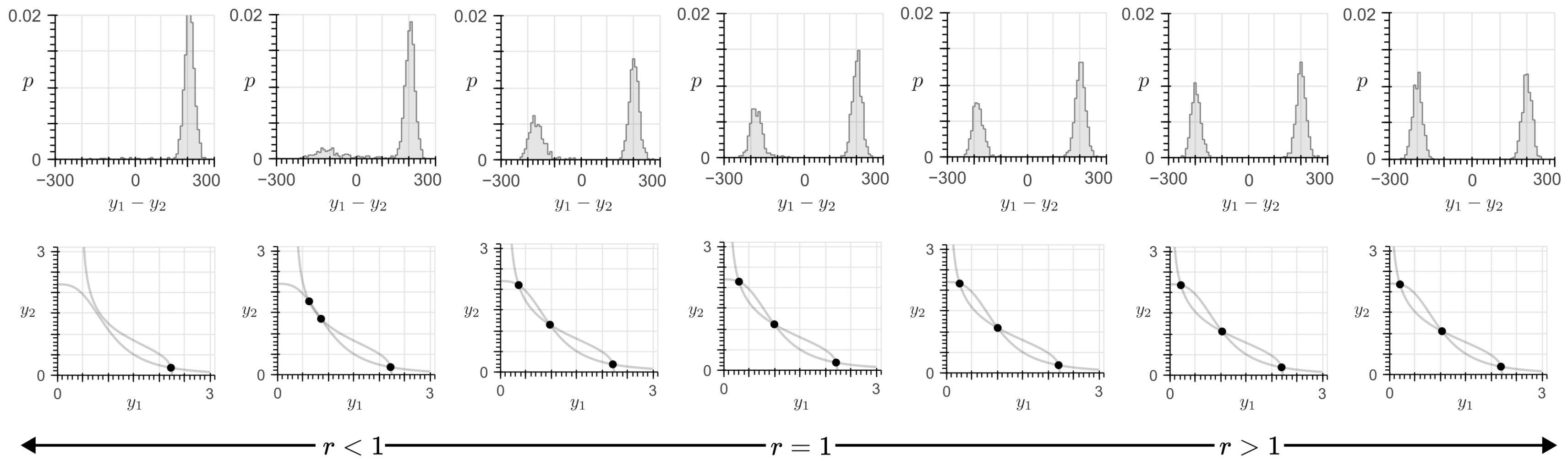

Figure S5: The controller must satisfy the adaptive metric requirement (**$r = \xi\delta/k\theta = 1$**). *A positive deviation (*$r > 1$*) decreases the bias in cell fate, but also reduces the perturbation in the equilibrium value. Conversely, a negative deviation (*$r < 1$*) increases the bias in cell fate, but also yields a larger perturbation in the equilibrium value. Simulations were done by numerically solving Eqs.* (26)-(30) *for the nominal values indicated in Table 1. The adaptive metric was varied within a* 20% *range of its nominal value* $r = 1$*, by either increasing the value of the production rate* k *(see Eq.* (29)*), thus yielding* $r < 1$*, or by decreasing it, thus yielding* $r > 1$*.*

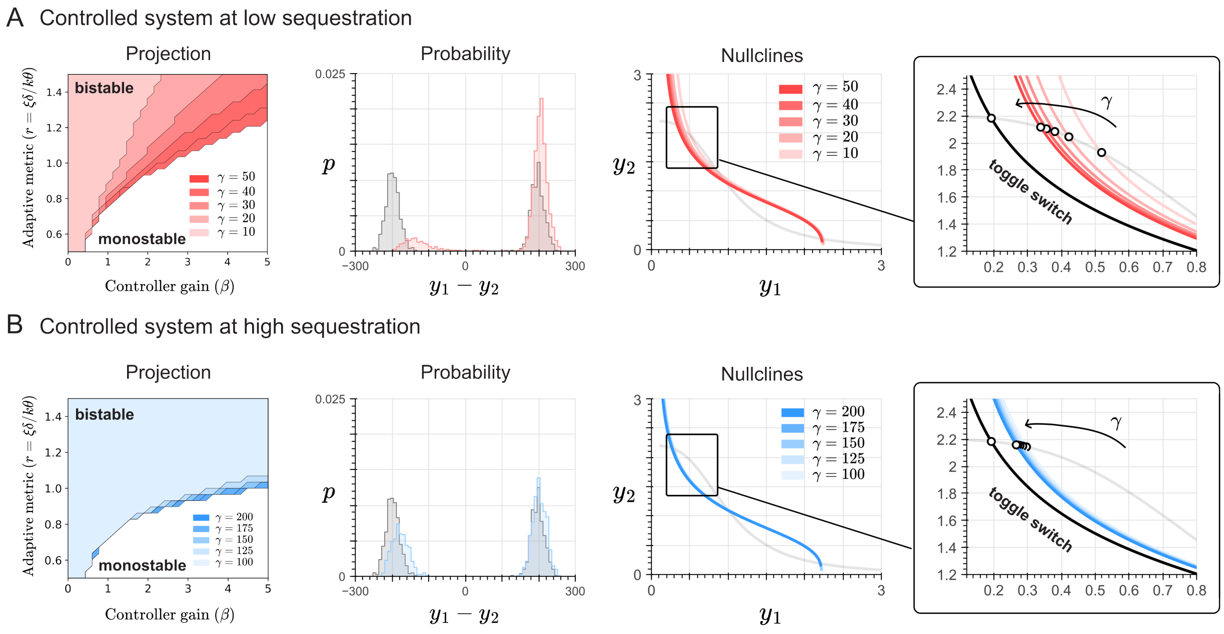

Figure S6: The dynamic properties of the adaptive controller rely on the high sequestration regime. *(A) The contour plot shows the combinations of controller gain (β) and adaptive metric (r) that result in bistability in the controlled toggle switch, for increasing values of the sequestration rate (γ). The red color gradient represents the "slow" sequestration regime, where the darkest red corresponds to $\gamma = 50$, which is half the nominal value ($\gamma = 100$). The white region indicates monostability. The probability distribution for $\gamma = 30$ is shown in red, displaying a skewed distribution that reflects the biased cell fate, compared to the symmetric, bimodal distribution of the isolated toggle switch shown in gray. The corresponding nullclines are shown to the right: the nullcline for the steady state of y_1 is plotted in gray, while that of y_2 is shown in a red gradient. To the right of the nullclines, we show the deviation of the asymptotically stable equilibrium with high expression of the Y_2 species and low expression of the Y_1 species, computed for the controlled system, relative to the corresponding equilibrium of the isolated toggle switch (shown in black).(B) Same analysis as in (A), now for the "fast" sequestration regime. The blue color gradient corresponds to increasing γ, where the darkest blue represents $\gamma = 200$, which is twice the nominal value. The probability distribution shown was computed for $\gamma = 150$. For both simulations, all probability distributions were obtained from 1000 stochastically perturbed trajectories initialized at the origin.*

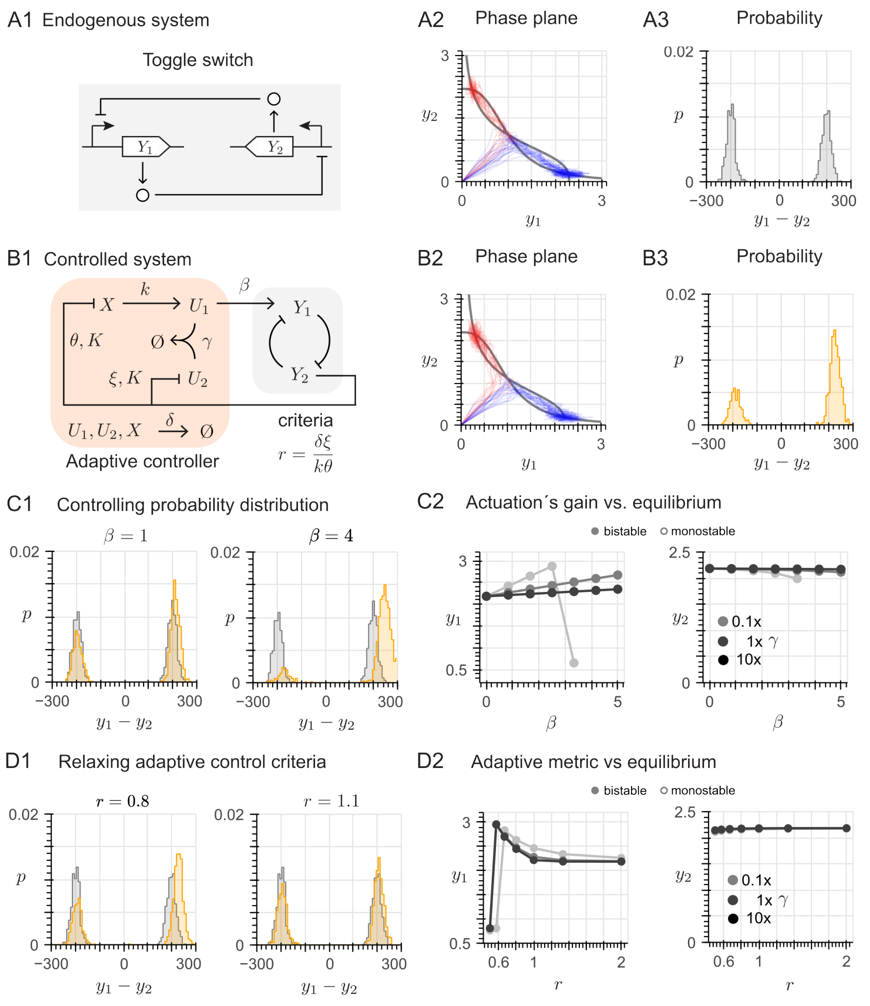

Figure S7: ***The adaptive controller with a positive feedback architecture enables a biased cell fate.*** *From the main text, we include panels A1-A3 for comparison: (A1) Architecture of the toggle switch, formed by two mutually inhibiting transcription factors* Y_1 *and* Y_2*. (A2) For the toggle switch, in the phase plane, the nullclines (gray lines) are shown along with* 1000 *stochastically perturbed trajectories starting from the origin; each trajectory converges either to the asymptotically stable equilibrium with high expression of* Y_1 *and low expression of* Y_2 *(blue trajectories) or to the asymptotically stable equilibrium with high expression of* Y_2 *and low expression of* Y_1 *(red trajectories). (A3) The trajectories in (A2) converge with comparable probability to either of the two asymptotically stable equilibria, resulting in an unbiased bimodal probability distribution. (B1) Architecture of the controlled system: a feedback loop of the toggle switch and the proposed adaptive controller,* ***with a positive feedback architecture****. (B2) For the controlled system, in the phase plane, the nullclines (gray lines) are shown along with* 1000 *stochastically perturbed trajectories starting from the origin; more trajectories converge to the asymptotically stable equilibrium with high expression of* Y_1 *(blue) and less to the asymptotically stable equilibrium with high expression of* Y_2 *(red). (B3) The controller yields a biased cell fate: the trajectories in B2 converge with higher probability to the equilibrium where the production of* Y_1 *is favored over that of* Y_2*. (C1) Increasing the control gain* β *increases the bias in the cell fate, leading to a larger imbalance in the probability distribution. (C2) Equilibrium values (of* Y_1 *for the equilibrium associated with high expression of* Y_1*, and of* Y_2 *for the equilibrium associated with high expression of* Y_2*) for increasing control gain* β*, for different values of the sequestration rate* γ *(*0.1*,* 1 *and* 10 *times its nominal value). (D1) Effect of a* 10% *increase and a* 20% *decrease of the adaptive metric* $r = \frac{\xi\delta}{k\theta}$ *from its nominal value* 1*: a biased cell fate is still generated at the price of a small alteration of the equilibrium values. (D2) Equilibrium values for varying adaptive metric* $r = \frac{\xi\delta}{k\theta}$*, for different values of the sequestration rate* γ *(*0.1*,* 1 *and* 10 *times its nominal value).*

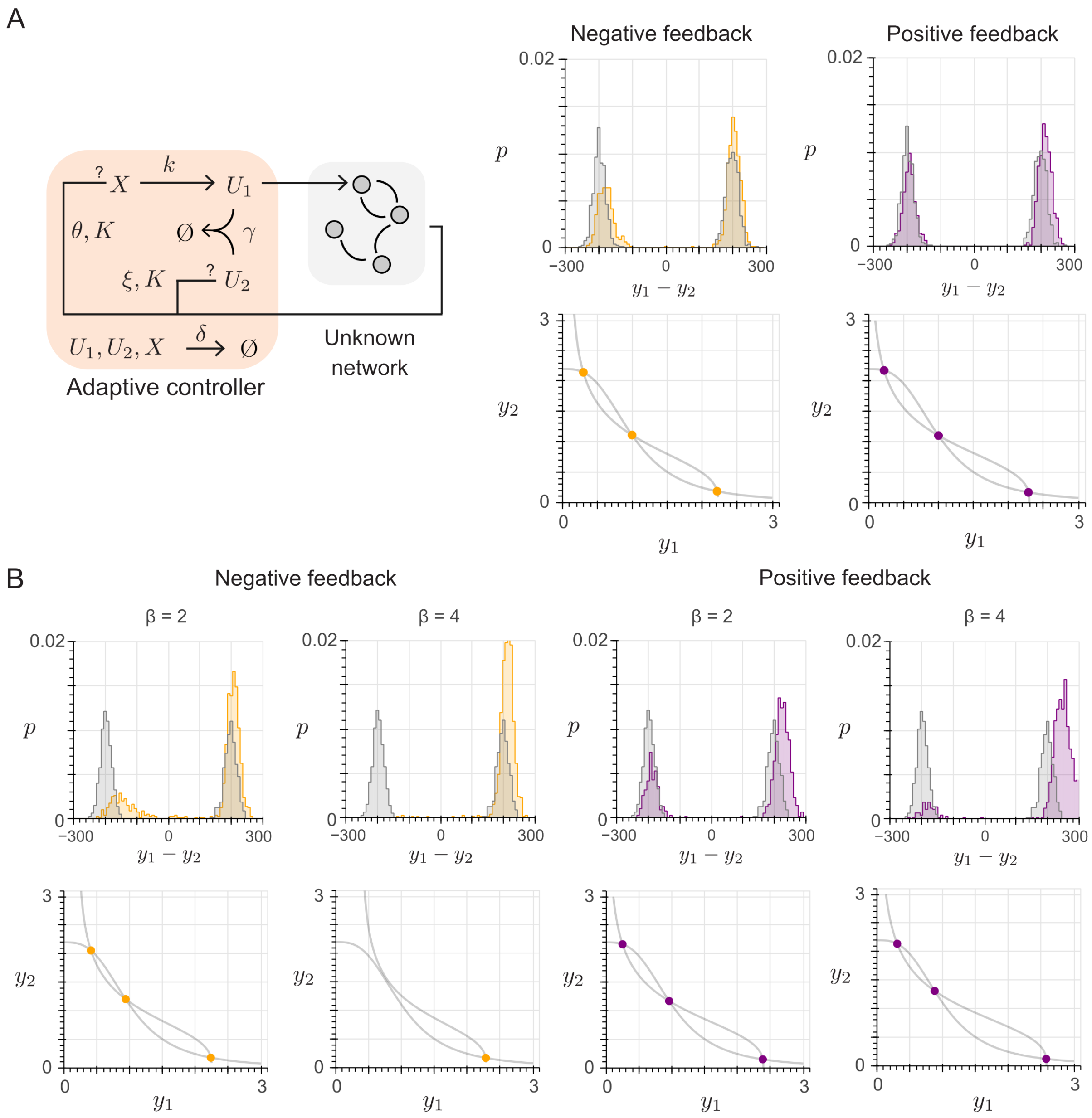

Figure S8: The adaptive controller is effective both with a negative and with a positive feedback architecture. *(A) When both the high sequestration rate and the ideal adaptive metric requirements are satisfied, our numerical simulations show that the effectiveness of the proposed adaptive controller is independent of the type of feedback provided by the endogenous gene regulatory network. Both architectures (negative and positive feedback) of the adaptive controller with low control gain values ($\beta \leq 1$, for the toggle switch example) induce the desired behavior (i.e, biased cell fate and small equilibrium alteration). (B) With higher control gain values ($\beta > 1$ for the toggle switch example), the negative feedback architecture enables a higher probability for the production of the target species Y_1, at the price of a higher perturbation to the equilibrium. (C) With higher control gain values ($\beta > 1$ for the toggle switch example), the positive feedback architecture reduces the perturbation to the equilibrium, but requires a higher β value to achieve the same probability distribution as the negative feedback architecture.*

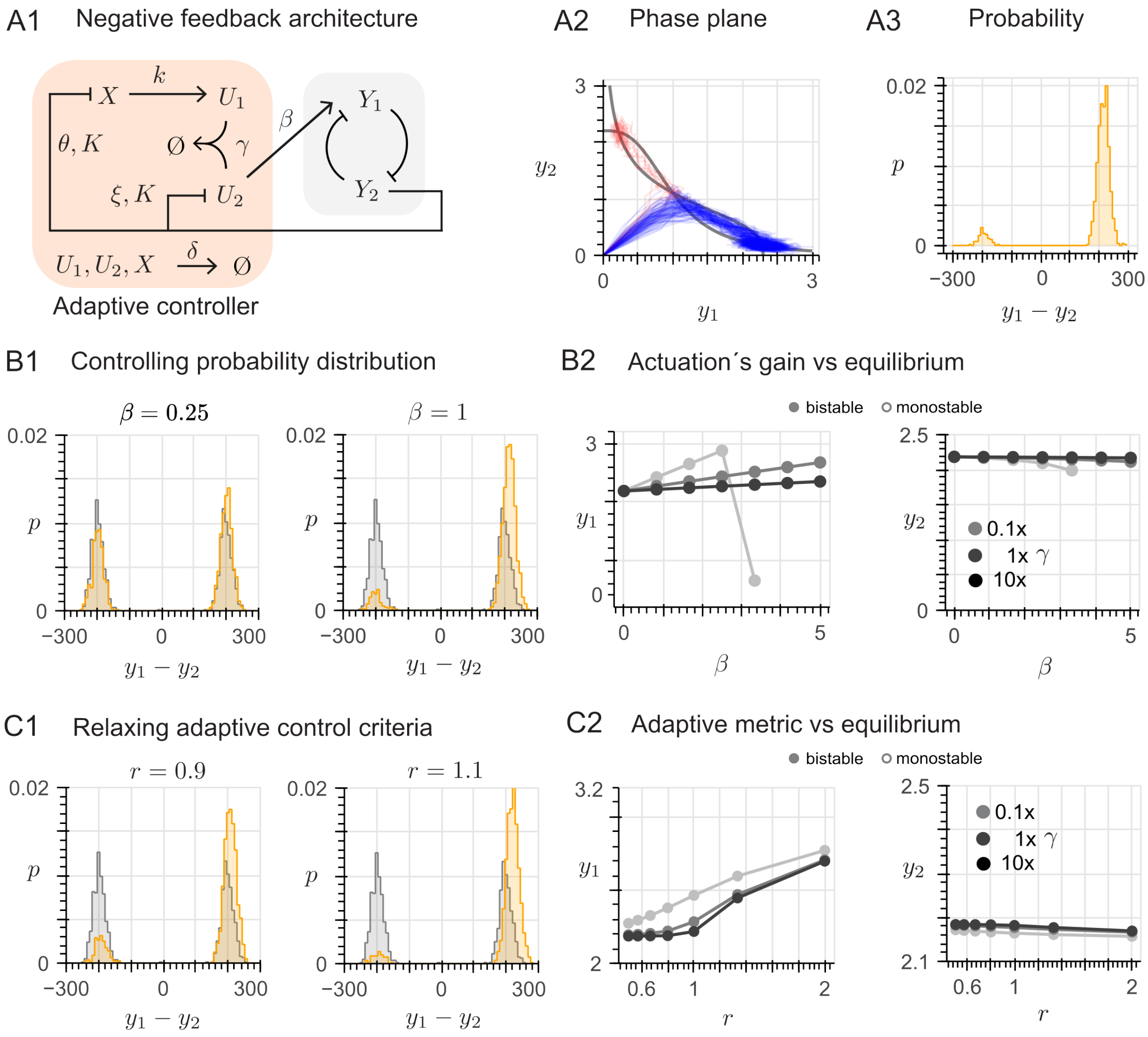

Figure S9: The adaptive controller is symmetric with respect to its actuation: another negative feedback architecture. *(A1) Architecture of the toggle switch with the adaptive controller, where Y_2 is the feedback species, and the controller's actuation is enforced via species U_2; overall, this corresponds to a negative feedback architecture. (A2) For the controlled system, in the phase plane, the nullclines (gray lines) are shown along with 1000 stochastically perturbed trajectories starting from the origin; more trajectories converge to the asymptotically stable equilibrium with high expression of Y_1 (blue) and less to the asymptotically stable equilibrium with high expression of Y_2 (red). (A3) The controller yields a biased cell fate: the trajectories in A2 converge with higher probability to the equilibrium where the production of Y_1 is favored over that of Y_2. (B1) Increasing the control gain β increases the bias in the cell fate, leading to a larger imbalance in the probability distribution. (B2) Equilibrium values (of Y_1 for the equilibrium associated with high expression of Y_1, and of Y_2 for the equilibrium associated with high expression of Y_2) for increasing control gain β, for different values of the sequestration rate γ (0.1, 1 and 10 times its nominal value). (C1) Effect of a 10% deviation of the adaptive metric $r = \frac{\xi\delta}{k\theta}$ from its nominal value 1: a biased cell fate is still generated at the price of a small alteration of the equilibrium values. (C2) Equilibrium values for varying adaptive metric $r = \frac{\xi\delta}{k\theta}$, for different values of the sequestration rate γ (0.1, 1 and 10 times its nominal value).*

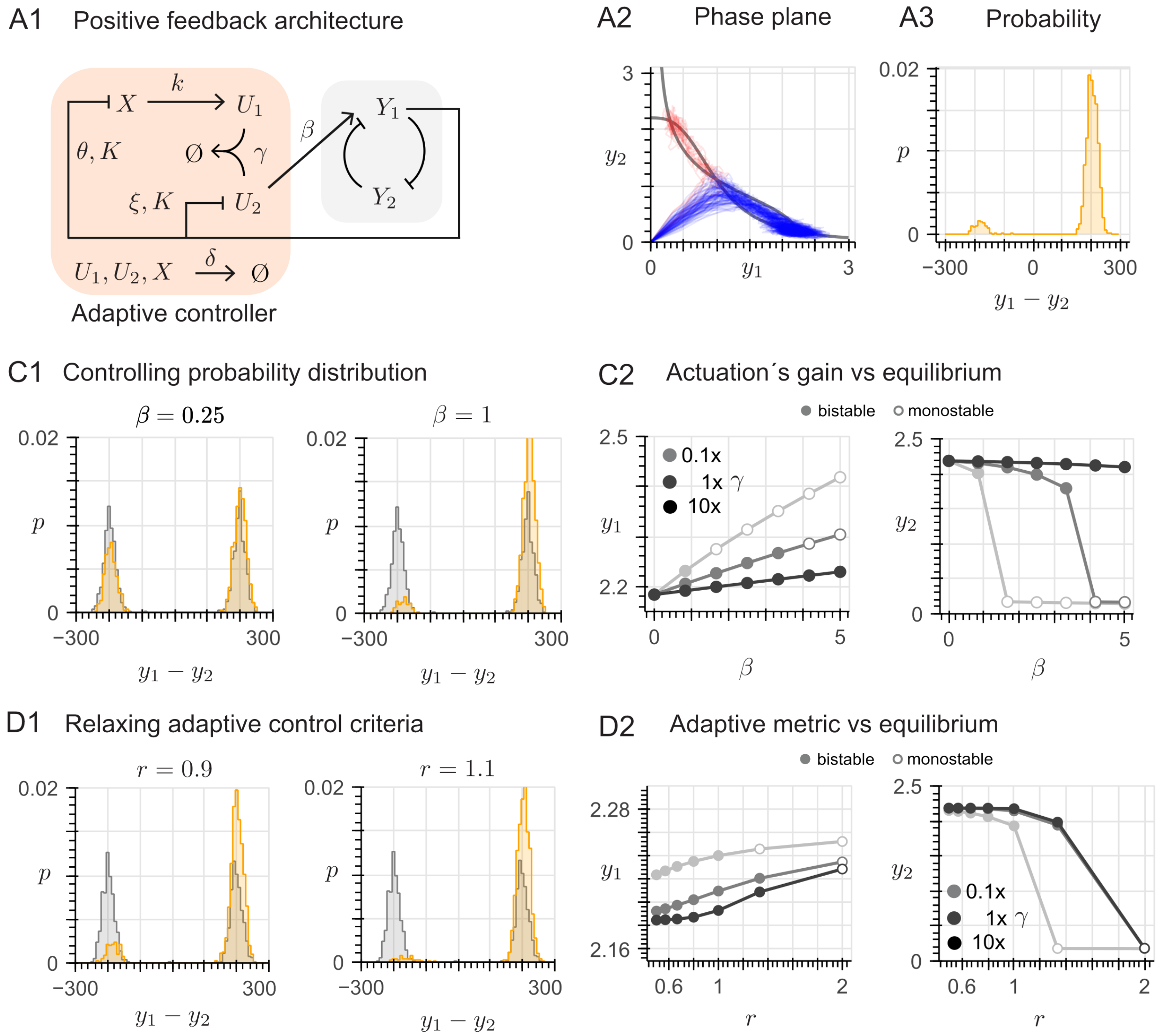

Figure S10: The adaptive controller is symmetric with respect to its actuation: another positive feedback architecture. *(A1) Architecture of the toggle switch with the adaptive controller, where Y_1 is the feedback species, and the controller's actuation is enforced via species U_2; overall, this corresponds to a positive feedback architecture. (A2) For the controlled system, in the phase plane, the nullclines (gray lines) are shown along with 1000 stochastically perturbed trajectories starting from the origin; more trajectories converge to the asymptotically stable equilibrium with high expression of Y_1 (blue) and less to the asymptotically stable equilibrium with high expression of Y_2 (red). (A3) The controller yields a biased cell fate: the trajectories in A2 converge with higher probability to the equilibrium where the production of Y_1 is favored over that of Y_2. (B1) Increasing the control gain β increases the bias in the cell fate, leading to a larger imbalance in the probability distribution. (B2) Equilibrium values (of Y_1 for the equilibrium associated with high expression of Y_1, and of Y_2 for the equilibrium associated with high expression of Y_2) for increasing control gain β, for different values of the sequestration rate γ (0.1, 1 and 10 times its nominal value). (C1) Effect of a 10% deviation of the adaptive metric $r = \frac{\xi\delta}{k\theta}$ from its nominal value 1: a biased cell fate is still generated at the price of a small alteration of the equilibrium values. (C2) Equilibrium values for varying adaptive metric $r = \frac{\xi\delta}{k\theta}$, for different values of the sequestration rate γ (0.1, 1 and 10 times its nominal value).*

4 Robustness analysis under kinetic mutations

We report here a deeper analysis of the robustness properties of the closed-loop system with respect to variation in the kinetic parameters. We refer to *ratio* as the proportion between α_2 and α_1, given the following ODEs that describe the isolated toggle switch,

$$\begin{aligned}
\dot{y}_1 &= \alpha_1 \frac{K^m}{K^m + y_2^m} - \delta y_1 \\
\dot{y}_2 &= \alpha_2 \frac{K^m}{K^m + y_1^m} - \delta y_2
\end{aligned}$$

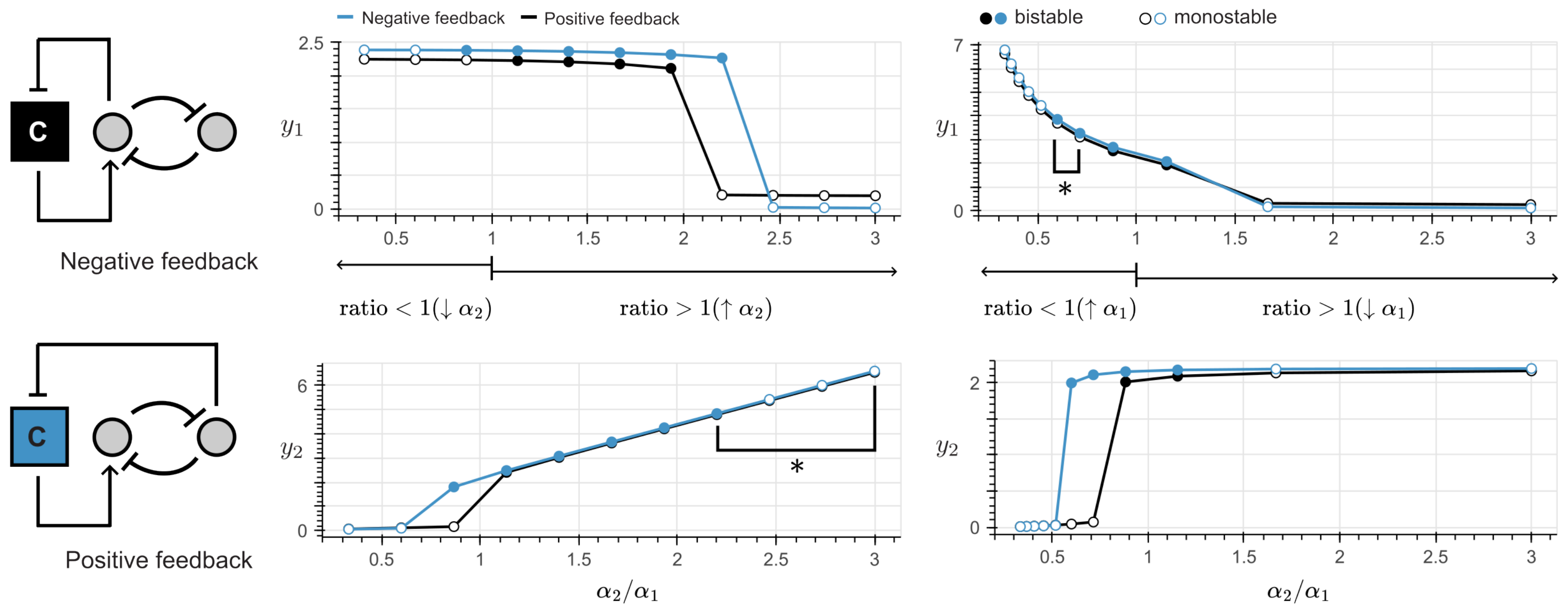

Figure S11: The positive feedback architecture (blue) is more robust than the negative feedback architecture (black). *For small values of the control gain, $\beta \leq 1$, both architectures exhibit similar dynamic properties (Fig. S8-A), and their robustness to parameter variations is similar (not shown here). For larger values of the control gain (for this example, $\beta = 2$), the positive feedback architecture exhibits a wider range of α_2/α_1 values for which the bistable behavior is maintained. In the top right and bottom left panels, the points highlighted with symbol $*$ correspond to monostability of the black line, overlapped by the blue line.*

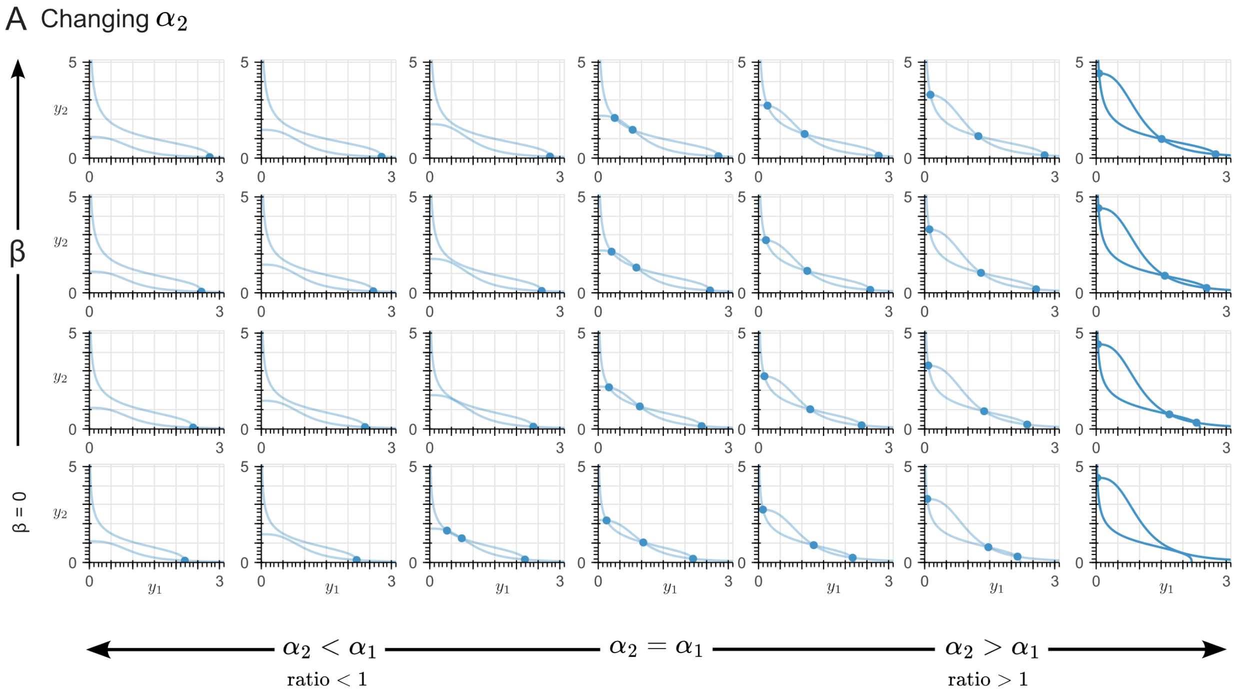

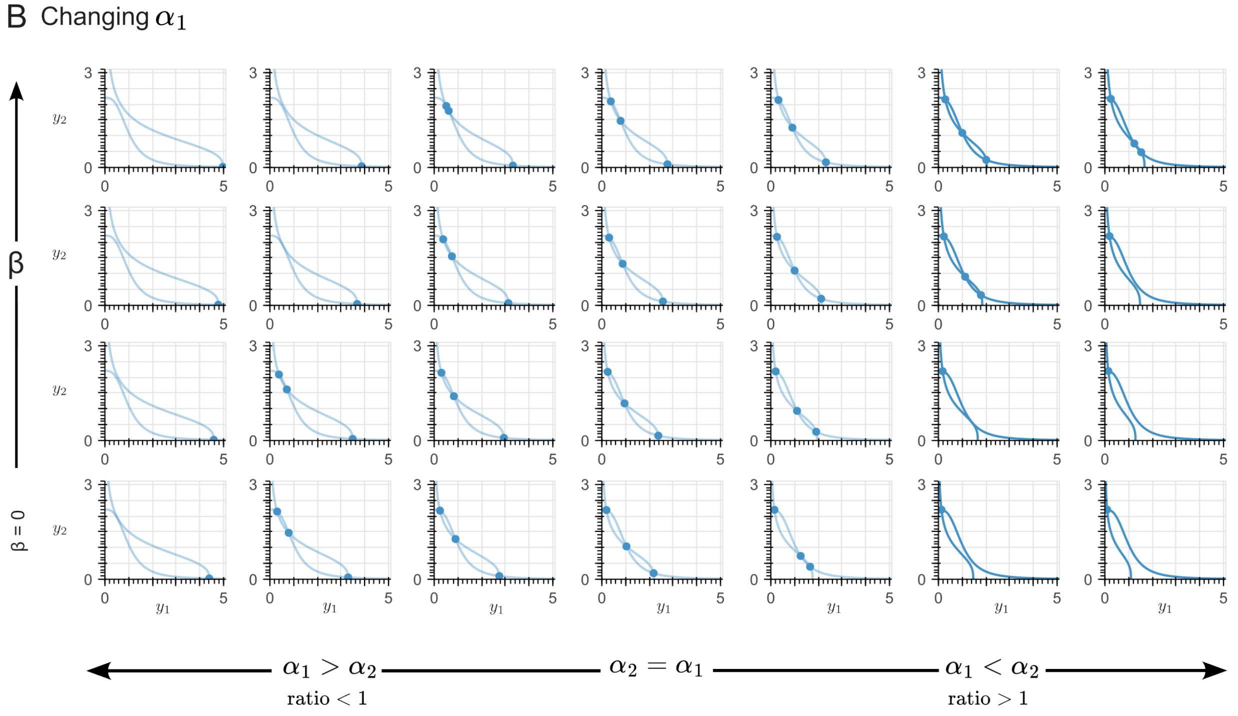

Figure S12: Phase plane of the controlled system with the positive feedback architecture for increasing values of the control gain β. *The highlighted cases (the last column in Panel A and the last two columns in Panel B) illustrate how increasing β results in an increased range of α_2/α_1 values for which bistability is maintained. From bottom to top, the control gain values are 1, 2 and 3 times the nominal value $\beta = 1$.*

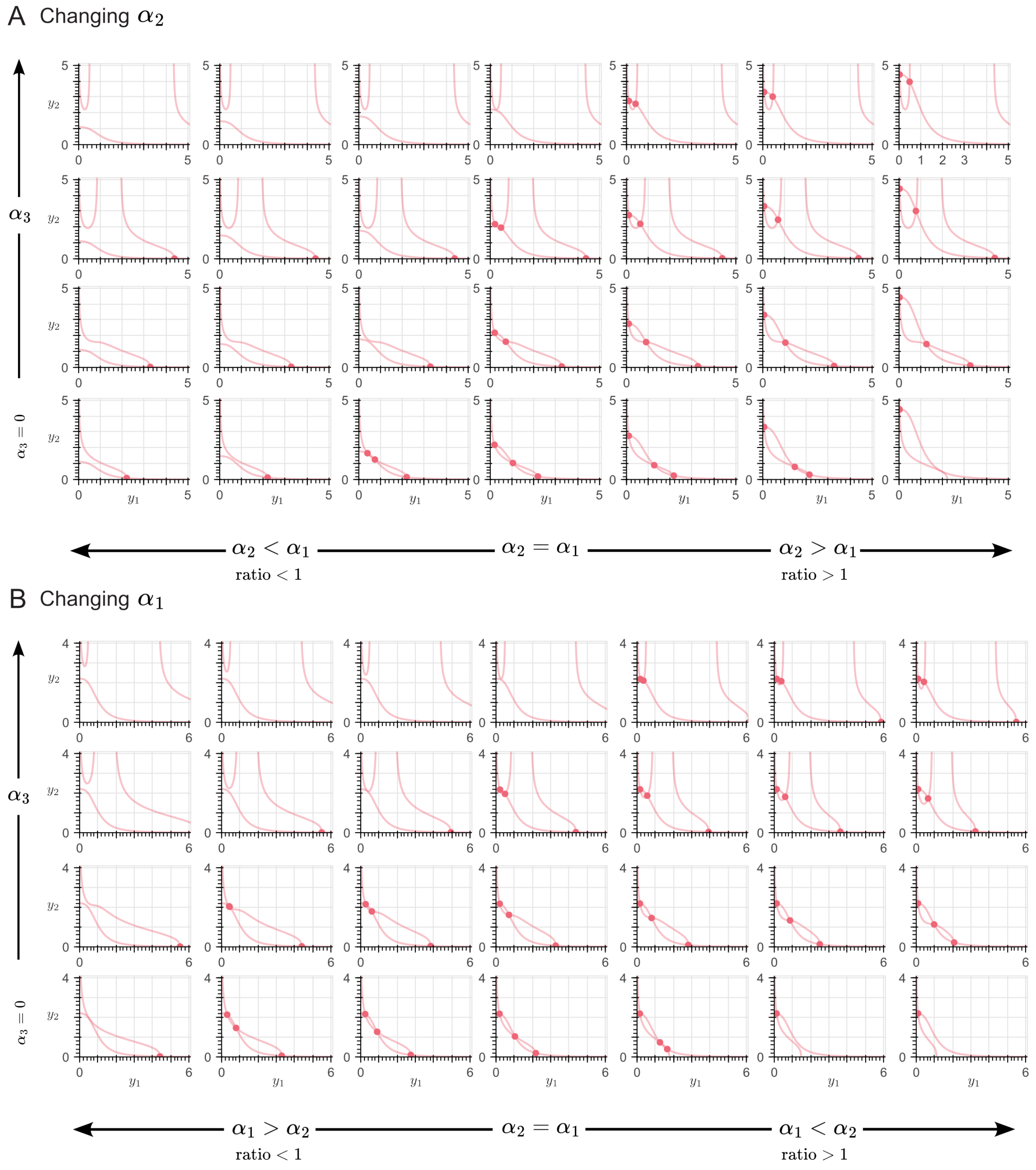

Figure S13: Phase plane of the toggle switch with a self-activation motif in the Y_1 species for increasing values of the activation strength α_3. *The increased robustness for mutations where ratio > 1 correlates with the modification of the nullcline shape, observed for high values of α_3. From top to bottom, the activation strength values are 1, 2 and 3 times the nominal value $\alpha_3 = 2.2$.*

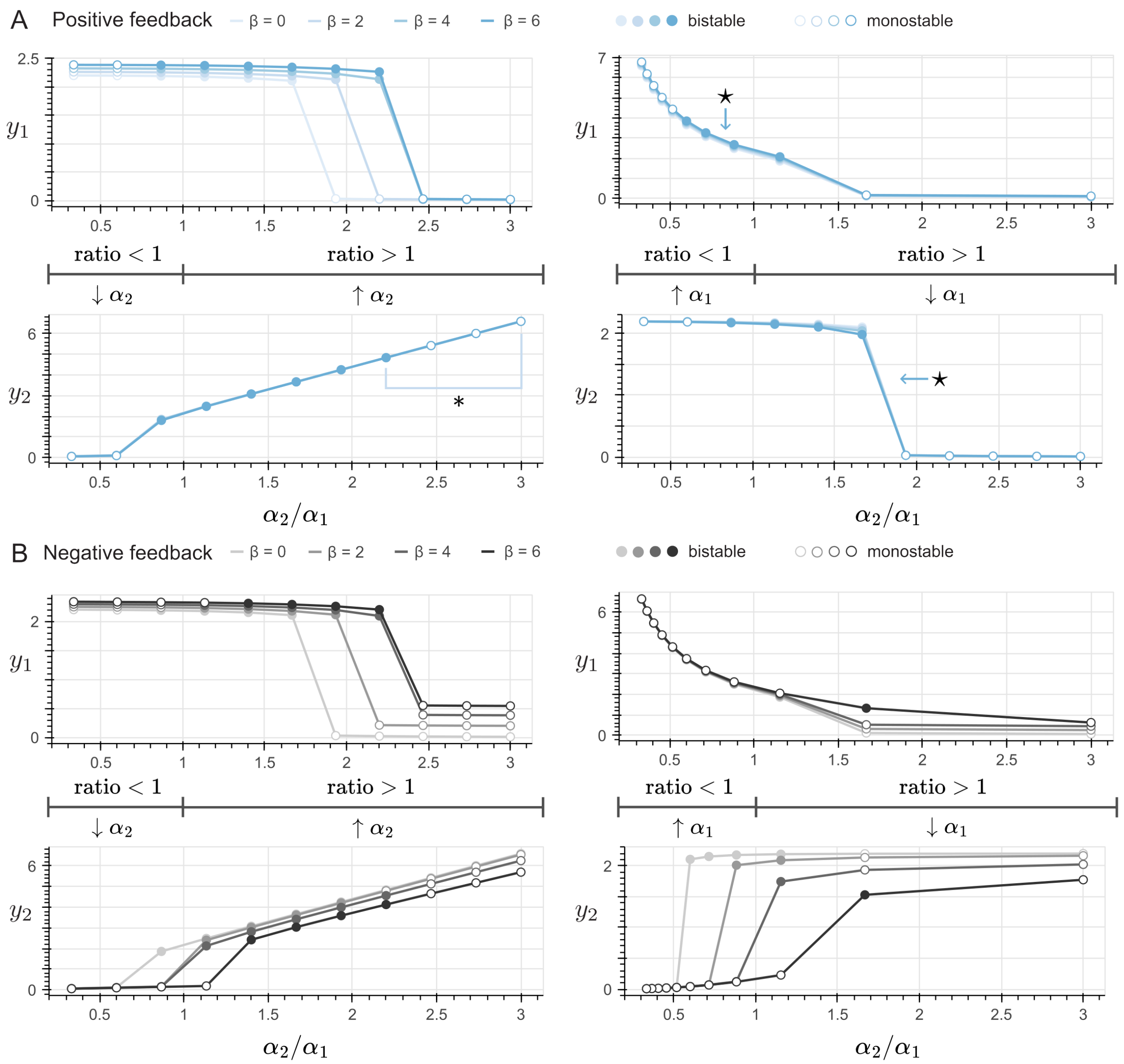

Figure S14: Increasing the robustness of the negative and positive feedback through the control gain *β. (A) For the controlled system with the positive feedback architecture, for mutations where ratio > 1 due to changes in α_2 and where ratio < 1 due to changes in α_1, increasing the gain β yields a wider range of α_2/α_1 values for which the bistable behavior is maintained. In the bottom left panel, the points highlighted with symbol $*$ correspond to monostability of the light blue lines corresponding to $\beta = 0$ and $\beta = 2$, overlapped by the darker blue lines. Likewise, in the right panels, all lines highlighted with symbol $\star$ are overlapping and exhibit the same stability properties for mutations where α_1 changes. (B) For the negative feedback architecture, for mutations where ratio > 1 due to changes in α_2, the strategy of increasing β is valid. However, for mutations where ratio < 1 due to changes in α_1, increasing β is counterproductive. In particular, for $\beta \geq 4$ there is a single value of α_2/α_1 where the system exhibits bistability.*

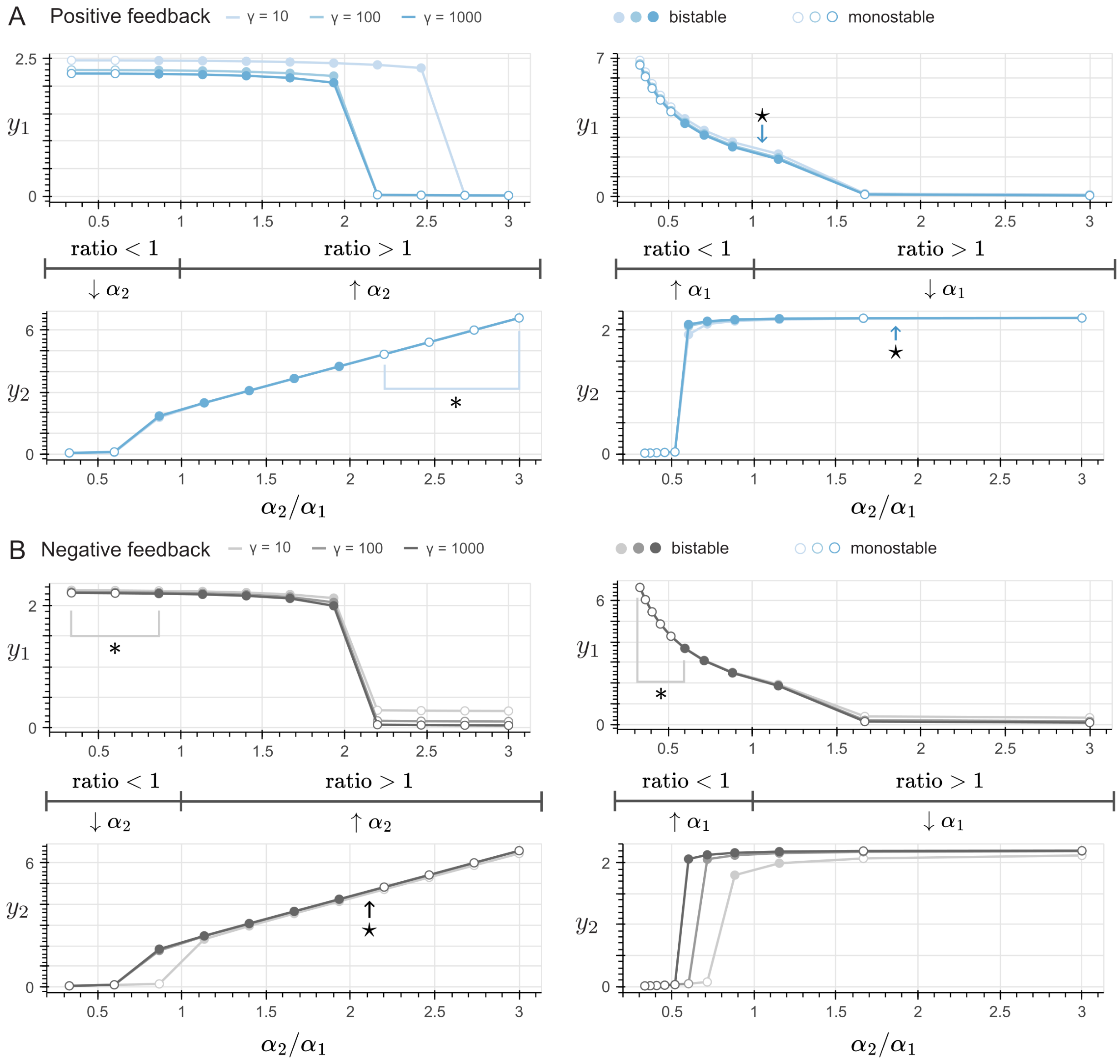

Figure S15: Increasing the robustness of the negative and positive feedback through the sequestration rate **γ.** *(A) For the controlled system with the positive feedback architecture, for mutations where ratio > 1 due to changes in* α_1 *and ratio < 1 due to changes in* α_2*, the range of* α_2/α_1 *values for which the system exhibits bistability can be extended by increasing the sequestration rate* γ *(ten-fold for a noticeable change). In the bottom left panel, the points highlighted with symbol* $*$ *correspond to monostability of the light blue lines, overlapped by the darker blue lines. Likewise, in the right panels, all lines highlighted with symbol* $\star$ *symbol are overlapping and exhibit the same stability properties for mutations where* α_1 *changes. (B) For the negative feedback architecture, in a very high sequestration regime (large* γ*), the range of* α_2/α_1 *values for which the system exhibits bistability is similar to the positive feedback architecture. In the top left and right panels, the points highlighted with symbol* $*$ *correspond to monostability of the light gray lines corresponding to* $\gamma = 10$ *(top left) and to* $\gamma = 10$ *and* $\gamma = 100$ *(top right panel), respectively, overlapped by the darker grey lines. Likewise, in the bottom left panel, all lines highlighted with symbol* $\star$ *are overlapping and exhibit the same stability properties for mutations where* α_2 *changes.*

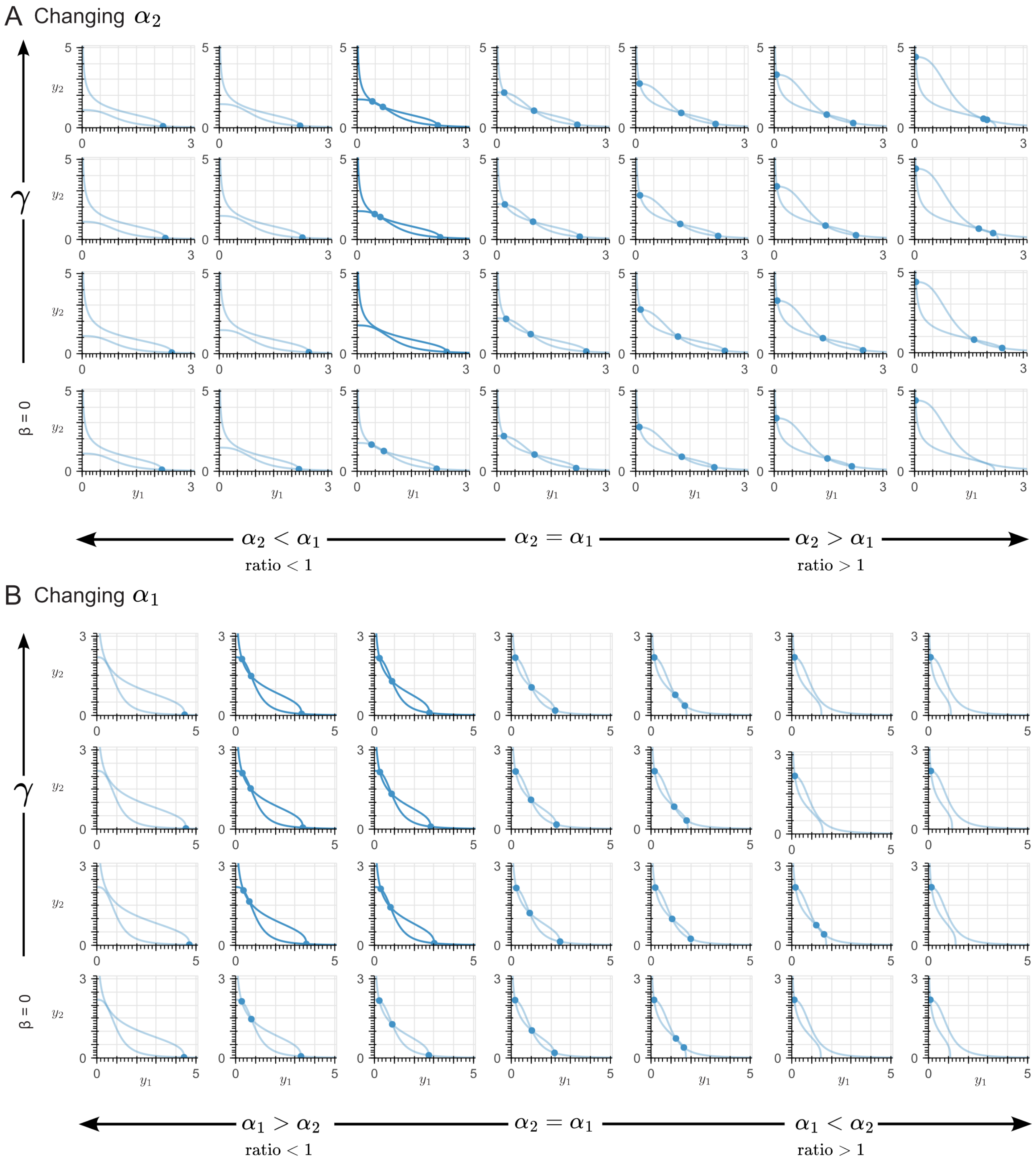

Figure S16: Phase plane of the controlled system with the positive feedback architecture for increasing values of the sequestration rate γ***.*** *The highlighted cases (the third column in Panel A, and the second and third columns in Panel B) illustrate how a significantly larger sequestration rate* γ *results in an increased range of* α_2/α_1 *values for which bistability is maintained. From top to bottom, the sequestration rate values are* 0.1, 1 *and* 10 *times the nominal value* $\gamma = 100$.

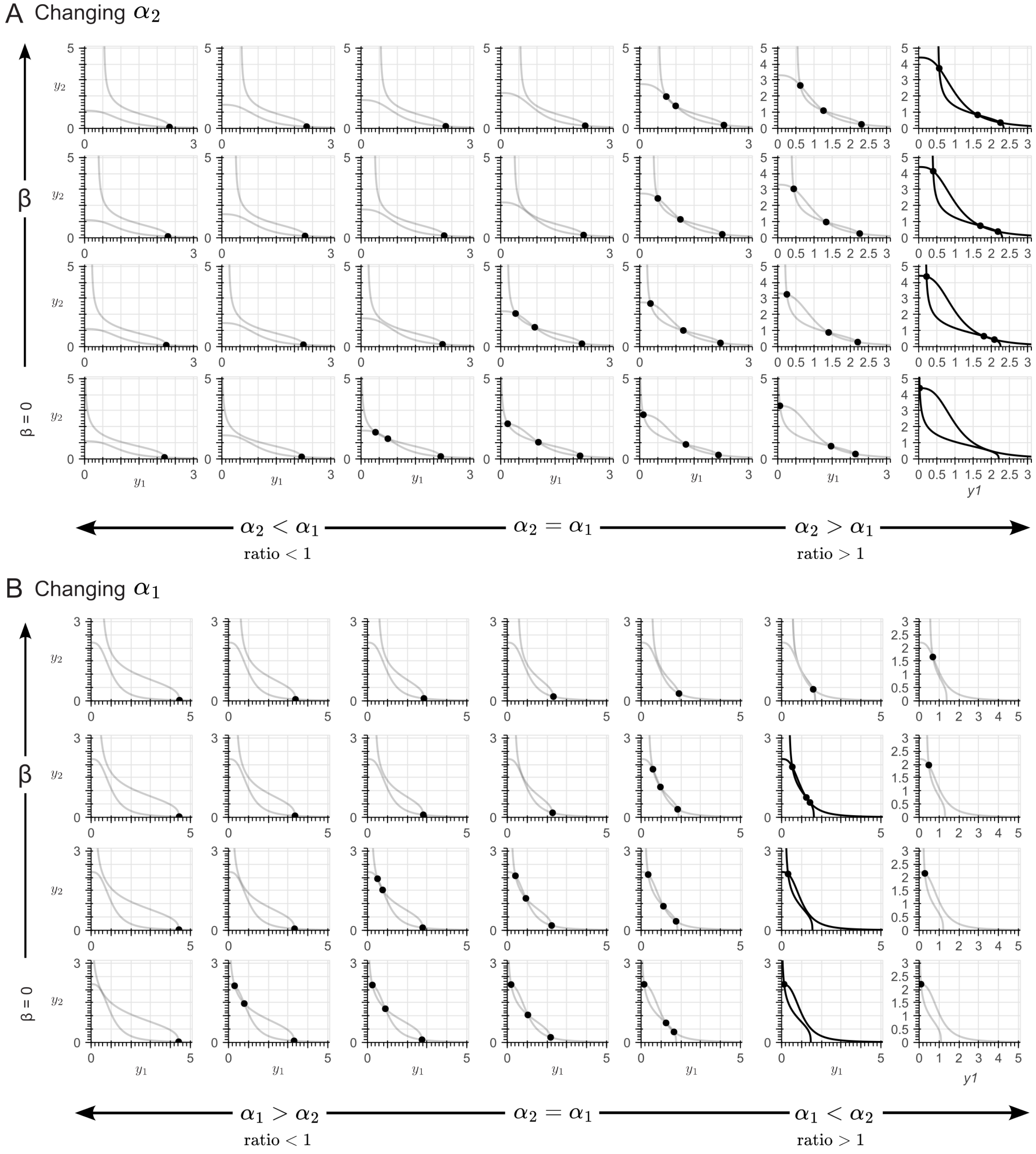

Figure S17: Phase plane of the controlled system with the negative feedback architecture for increasing values of the control gain β. *The highlighted cases (the last column in Panel A, and the penultimate column in Panel B) illustrate how increasing β results in an increased range of α_2/α_1 values for which bistability is preserved. From bottom to top, the control gain values are 1, 2 and 3 times the nominal value $\beta = 1$.*

A Changing α_2

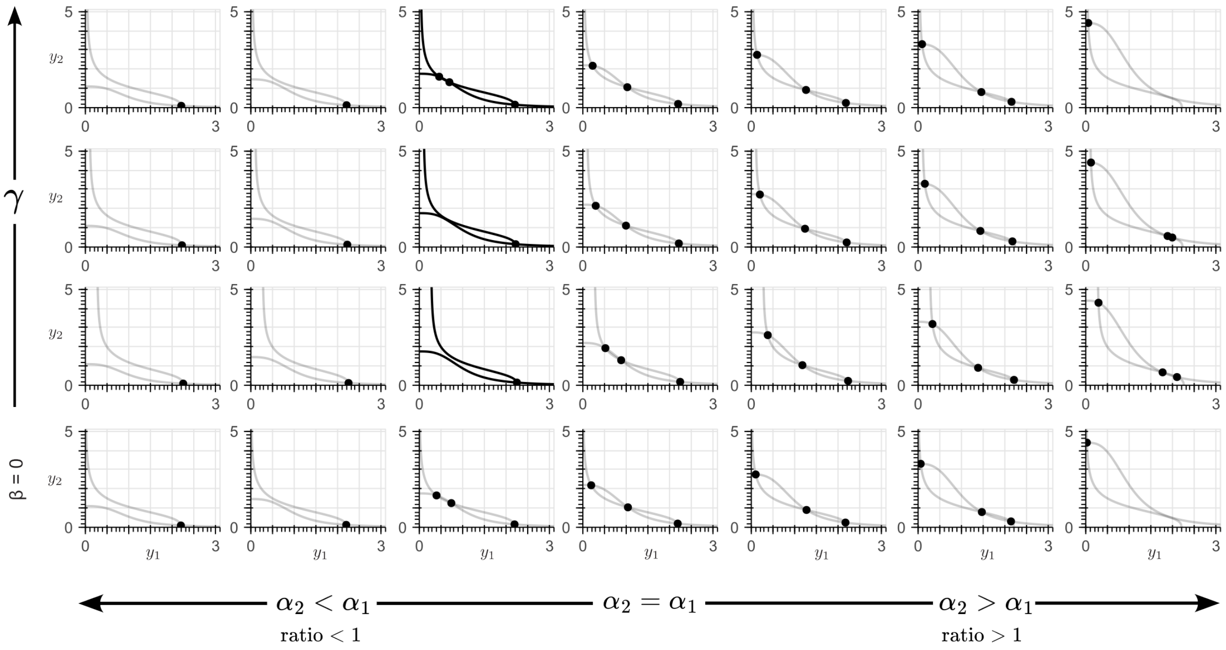

B Changing α_1

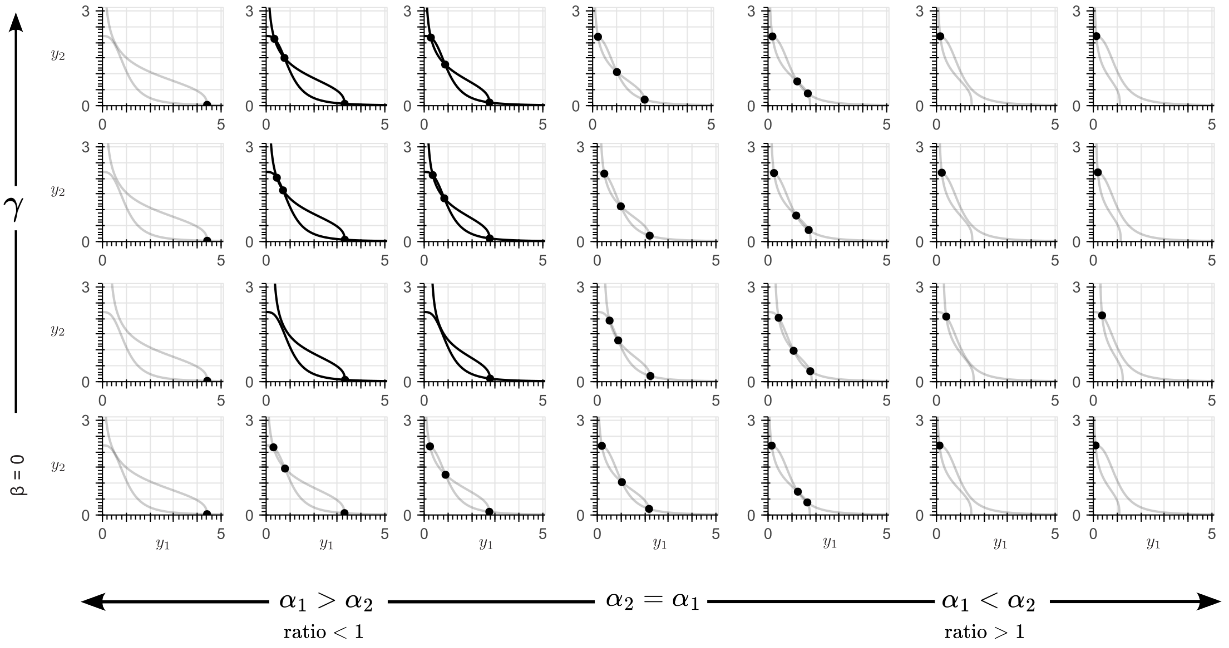

Figure S18: Phase plane of the controlled system with the negative feedback architecture for increasing values of the sequestration rate γ***.*** *The highlighted cases (the third column in Panel A, and the second and third columns in Panel B) illustrate how a significantly larger sequestration rate* γ *results in an increased range of* α_2/α_1 *values for which bistability is maintained. From top to bottom, the sequestration rate values are* 0.1, 1 *and* 10 *times the nominal value* $\gamma = 100$.

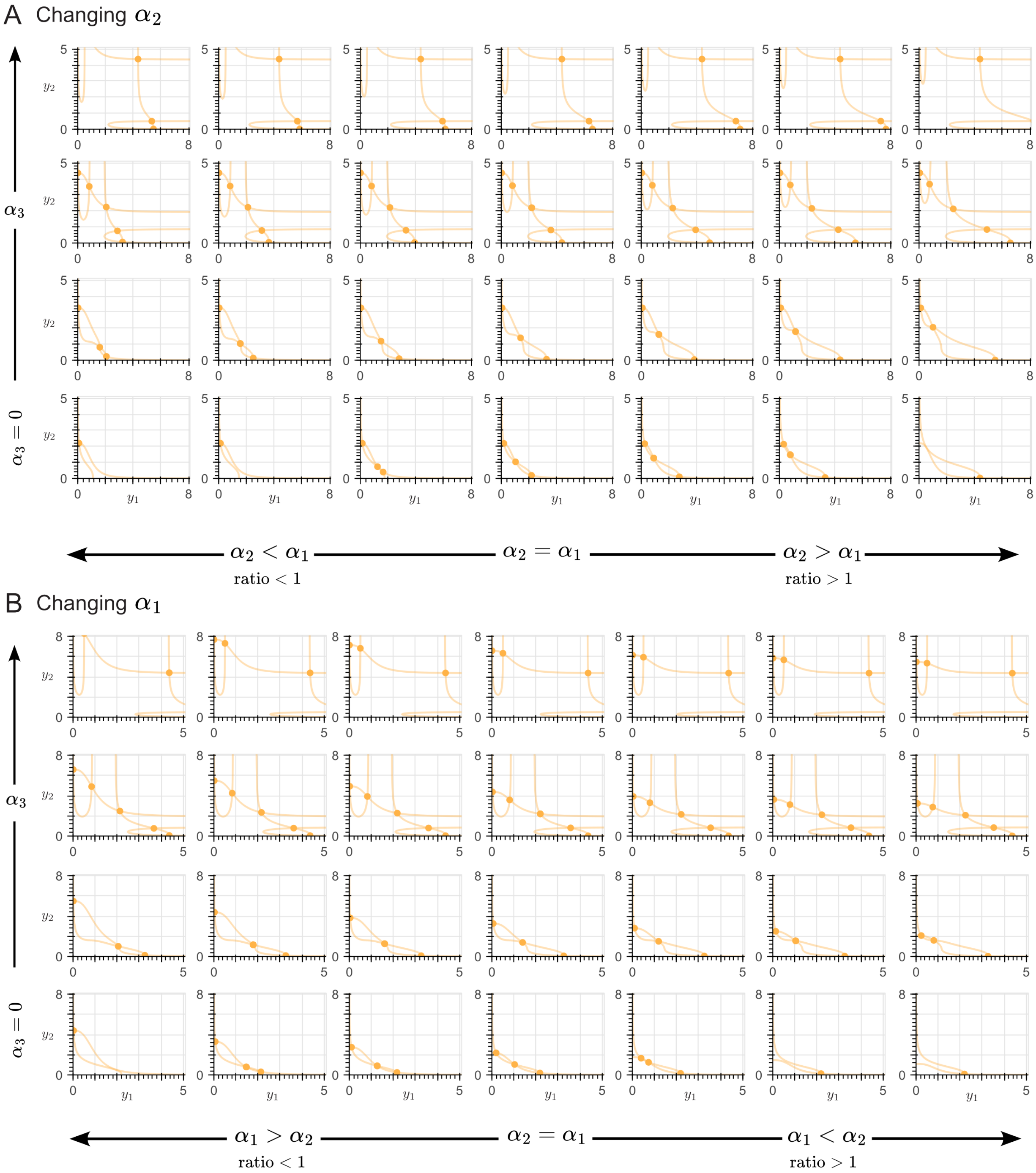

Figure S19: Phase plane of the toggle switch with a self-activation motif for each species Y_1 and Y_2, showing the effect of increasing values of the activation strength α_3. *The dynamics of the network can display two different regimes: bistability, observed for low α_3 values ($\alpha_3 = 1.1$, in this example) and tristability, associated with the appearance of a third (intermediate) equilibrium (for $\alpha_3 > 1.1$, in this example). From top to bottom, the activation strength values are 1, 2 and 3 times the nominal value $\alpha_3 = 2.2$.*

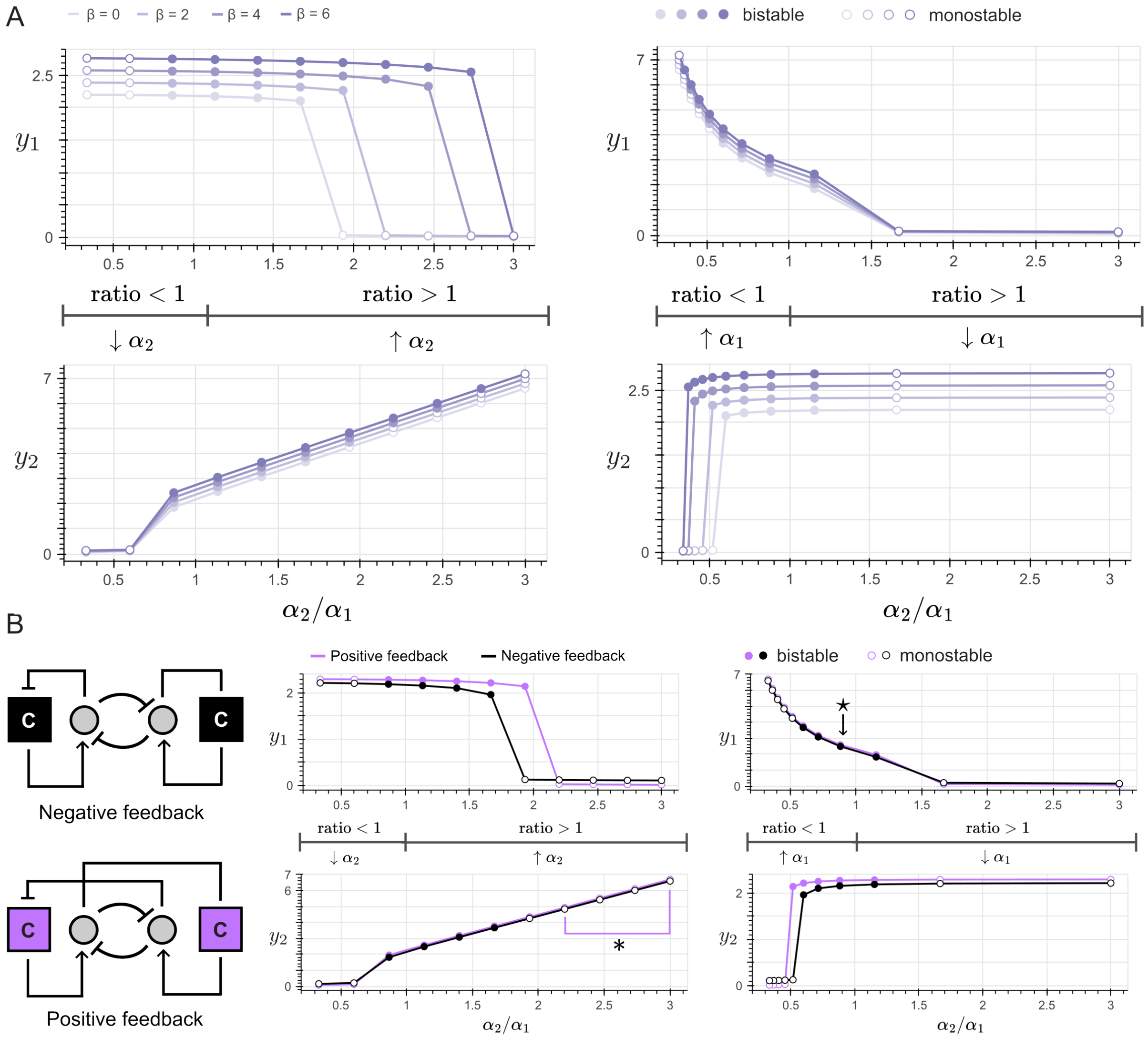

Figure S20: Increasing robustness through the addition of another adaptive controller. *(A) The controlled system with adaptive controllers enforced on the two species* Y_1 *and* Y_2*, with the positive feedback architecture and with a high control gain (*$\beta = \beta' = 6$ *for this example) exhibit robustness to virtually any type of mutation. (B) However, the same strategy is not suited for the negative feedback architecture, as the addition of another adaptive controller reduces the range of* α_2/α_1 *values for which bistability is maintained, compared to the application of a single adaptive controller. In the bottom left panel, the points highlighted with symbol* $*$ *correspond to monostability of the purple line, overlapped by the black line. Likewise, in the top right panel, lines highlighted with symbol* $\star$ *are overlapping and exhibit the same stability properties for mutations where* α_1 *changes.*

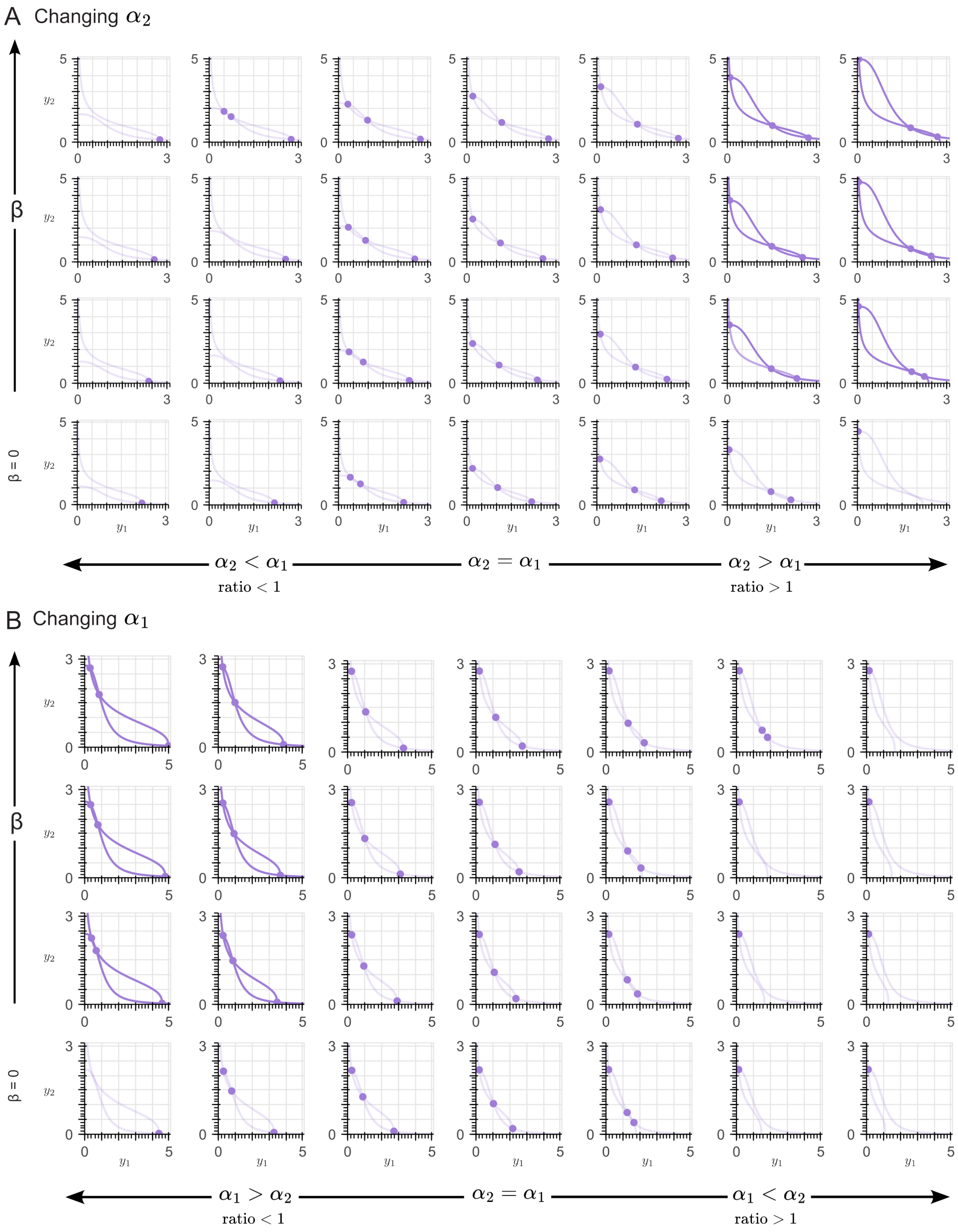

Figure S21: Phase plane of the controlled system with two adaptive controllers, each with the positive feedback architecture, showing the effect of increasing values of control gain (assumed $\beta = \beta'$ for simplicity). *The highlighted cases (the last two columns in Panel A, and the first three columns in Panel B) illustrate an overall increase in the system robustness for both mutations where ratio < 1 for increasing values in α_1, and where ratio > 1 for increasing values of α_2. From top to bottom, the control gain values are 1, 2 and 3 times the nominal value $\beta = \beta' = 1$.*

5 Extending the applications of the adaptive controller

Here, we report additional simulations of various application of the adaptive controller.

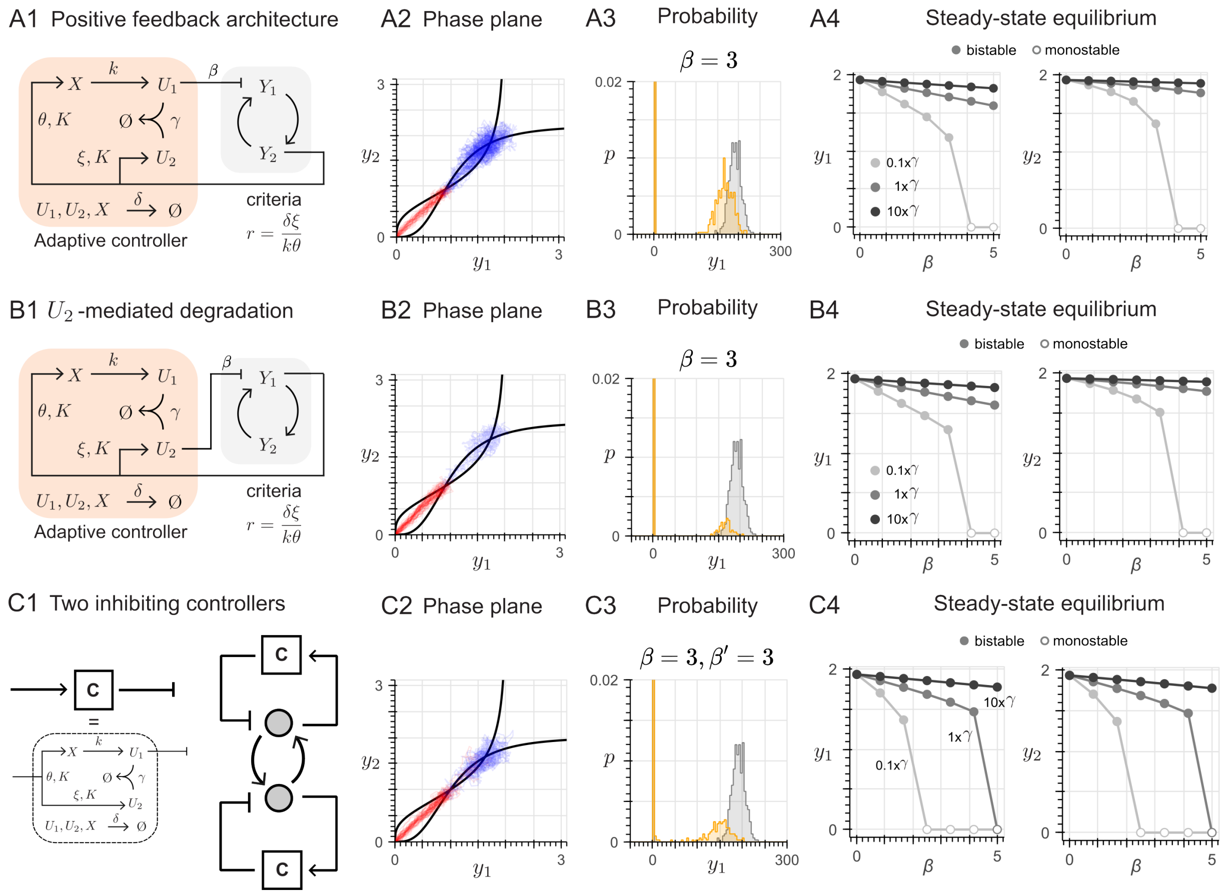

Figure S22: Alternative control strategies for the mutual activation network. *(A1) Architecture of the adaptive controller with the positive feedback architecture. (A2) For the controlled system, nullclines (black lines) and trajectories in the phase plane. (A3) Generation of a biased cell fate, which favors the equilibrium with no expression: the probability of converging to the equilibrium with high expression significantly decreases for both species when the control is present (orange distribution) with respect to the open-loop case (gray distribution). (A4) Characterization of the effect on the equilibrium value of increasing inhibition strength* β*, for different choices of the sequestration rate* γ*. (B1) Architecture of the adaptive controller with an inhibiting effect through species* U_2*. (B2)-(B4) Same analysis as in (A2)-(A4) applied to the system in B1. (C1) Architecture of two adaptive controllers with the negative feedback architecture, simultaneously exerting an inhibting effect through species* U_1 *and* U_1' *to both species* Y_1 *and* Y_2*, respectively. (C2)-(C4) Same analysis as in (A2)-(A4) applied to the system in C1.*

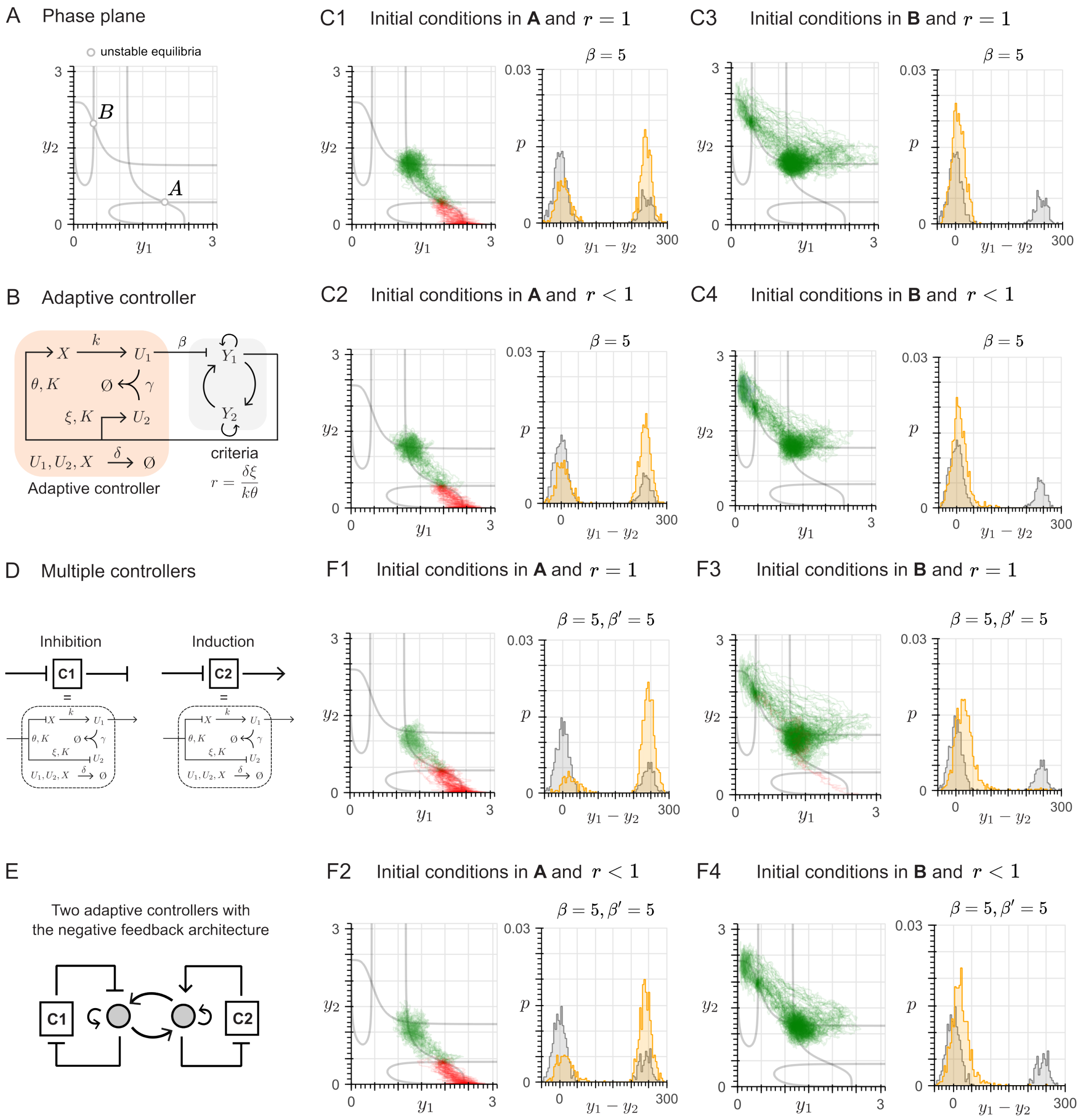

Figure S23: Design challenge: how can we favor the high production state of either Y_1 or Y_2? *(A) For the toggle switch with self-activation, the unstable equilibria, named A and B, are found at the intersection of the nullclines (light gray) and are shown as white circles. (B) Architecture of the adaptive controller with a negative feedback architecture. (C1) Generation of a biased cell fate for $\beta = 5$ and $r = 1$. Left: 1,000 stochastically perturbed trajectories starting from A are shown; more trajectories converge to the asymptotically stable equilibrium with high expression of Y_1 (red) and fewer to the stable equilibrium with intermediate, equal expression of both Y_1 and Y_2 (green). Right: The probability of converging to the equilibrium with high expression of Y_1 (orange distribution) increases compared to the open-loop case (gray distribution). (C2-C4) Same analysis as in (C1), for: (C2) trajectories starting in A and $r < 1$, (C3) trajectories starting from B and $r = 1$, and (C4) trajectories starting from B and $r < 1$. (D) The "inhibition" controller (C_1) implies that the effect of the U_1 species over the process is repressive, whereas the "induction" controller (C_2) implies that U_1 is activating. (E) An illustration of the toggle switch with self-activation under the proposed control strategy. Each controller exhibits a negative feedback architecture. The control gains (β for C_1, and β' for C_2) are assumed to be equal ($\beta = \beta' = 5$). (F1-F4) Same analysis as in (C1), for: (F1) trajectories starting in A and $r = 1$, (F2) trajectories starting in A and $r < 1$, (F3) trajectories starting in B and $r = 1$, and (F4) trajectories starting in B and $r < 1$.*

6 Models

6.1 Toggle switch and adaptive controller with negative feedback and U_1 actuation

For the closed-loop network introduced in Section 2.1.2 (Toggle switch: model description) in the main text, we report here chemical reactions and ordinary differential equations for ease of perusal.

Chemical reactions

$\emptyset \xrightarrow{\alpha p_1} Y_1,$	$\emptyset \xrightarrow{\alpha p_2} Y_2,$	Production
$Y_1 \xrightarrow{\delta} \emptyset,$	$Y_2 \xrightarrow{\delta} \emptyset,$	Decay
$\emptyset \xrightarrow{\theta p_2} X,$	$\emptyset \xrightarrow{\xi p_2} U_2,$	Production
$U_1 \xrightarrow{\beta} U_1 + Y_1$	$U_1 + U_2 \xrightarrow{\gamma} \emptyset$	Production/Sequestration
$U_1, U_2 \xrightarrow{\delta} \emptyset$	$X \xrightarrow{\delta} \emptyset$	Decay

where $p_1 = \frac{K^m}{y_2^m + K^m}$ and $p_2 = \frac{K^m}{y_1^m + K^m}$.

Ordinary Differential Equations

$$\dot{y}_1 = \alpha \frac{K^m}{y_2^m + K^m} - \delta y_1 + \beta u_1 \tag{26}$$

$$\dot{y}_2 = \alpha \frac{K^m}{y_1^m + K^m} - \delta y_2 \tag{27}$$

$$\dot{u}_1 = kx - \delta u_1 - \gamma u_1 u_2 \tag{28}$$

$$\dot{u}_2 = \xi \frac{K^m}{K^m + y_1^m} - \delta u_2 - \gamma u_1 u_2 \tag{29}$$

$$\dot{x} = \theta \frac{K^m}{K^m + y_1^m} - \delta x \tag{30}$$

Steady-state analysis

We can compute the system nullclines in the y_1-y_2 phase plane by equating $\dot{y}_1 = 0$ and $\dot{y}_2 = 0$, which yields the following expressions:

$$\bar{y}_1 = \sqrt[m]{\frac{\alpha K^m}{\delta \bar{y}_2} - K^m}$$

$$\bar{y}_2 = \sqrt[m]{\frac{\alpha K^m}{\delta \bar{y}_1 - \beta \bar{u}_1} - K^m}$$

We can further find $\bar{u}_1$ as a function of $\bar{y}_1$. First, since at the equilibrium $\dot{x} = 0$, it is $\bar{x} = \frac{\theta}{\delta} \frac{K^m}{K^m + \bar{y}_1^m}$. Then, by considering both $\dot{u}_1 = 0$ and $\dot{u}_2 = 0$, we have

$$\bar{u}_2 = \frac{f_1 - \delta \bar{u}_1}{\gamma \bar{u}_1} = \frac{f_2}{\gamma \bar{u}_1 + \delta},$$

where $f_1 = k\bar{x}$ and $f_2 = \xi \frac{K^m}{K^m + \bar{y}_1^m}$. This leads to a second order polynomial, $P(\bar{u}_1) = \bar{u}_1^2 + A\bar{u}_1 + B = 0$, which admits a single positive real solution

$$\bar{u}_1 = \frac{-A + \sqrt{A^2 - 4B}}{2},$$

where $A = \frac{f_2 - f_1}{\delta} + \frac{\delta}{\gamma}$ and $B = -\frac{f_1}{\gamma}$.

6.2 Toggle switch and adaptive controller with positive feedback and U_1 actuation

We consider here a modification of the previous network regarding the feedback species, as detailed in the main text and characterized in Fig. S7.

Chemical reactions

$\emptyset \xrightarrow{\alpha p_1} Y_1,$	$\emptyset \xrightarrow{\alpha p_2} Y_2,$	Production
$Y_1 \xrightarrow{\delta} \emptyset,$	$Y_2 \xrightarrow{\delta} \emptyset,$	Decay
$\emptyset \xrightarrow{\theta p_1} X$	$\emptyset \xrightarrow{\xi p_1} U_2$	Production
$U_1 \xrightarrow{\beta} U_1 + Y_1$	$U_1 + U_2 \xrightarrow{\gamma} \emptyset$	Production/Sequestration
$U_1, U_2 \xrightarrow{\delta} \emptyset$	$X \xrightarrow{\delta} \emptyset$	Decay

where $p_1 = \frac{K^m}{y_2^m + K^m}$ and $p_2 = \frac{K^m}{y_1^m + K^m}$.

Ordinary Differential Equations

$$\dot{y}_1 = \alpha \frac{K^m}{y_2^m + K^m} - \delta y_1 + \beta u_1 \quad (31)$$

$$\dot{y}_2 = \alpha \frac{K^m}{y_1^m + K^m} - \delta y_2 \quad (32)$$

$$\dot{u}_1 = kx - \delta u_1 - \gamma u_1 u_2 \quad (33)$$

$$\dot{u}_2 = \xi \frac{K^m}{K^m + y_2^m} - \delta u_2 - \gamma u_1 u_2 \quad (34)$$

$$\dot{x} = \theta \frac{K^m}{K^m + y_2^m} - \delta x \quad (35)$$

Steady-state analysis

We can compute the system nullclines in the y_1-y_2 phase plane by equating $\dot{y}_1 = 0$ and $\dot{y}_2 = 0$, which yields the following expressions:

$$\bar{y}_2 = \frac{\alpha}{\delta}\left(\frac{K^m}{K^m + \bar{y}_1^m}\right)$$

$$\bar{y}_1 = \frac{\alpha}{\delta}\frac{K^m}{K^m + \bar{y}_2^m} + \frac{\beta}{\delta}u_1$$

We can further find $\bar{u}_1$ as a function of $\bar{y}_2$. First, since at the equilibrium $\dot{x}=0$, it is $\bar{x}=\frac{\theta}{\delta}\frac{K^m}{K^m+\bar{y}_2^m}$. Then, by considering both $\dot{u}_1=0$ and $\dot{u}_2=0$, we have

$$\bar{u}_2=\frac{f_1-\delta\bar{u}_1}{\gamma\bar{u}_1}=\frac{f_2}{\gamma\bar{u}_1+\delta},$$

where $f_1=k\bar{x}$ and $f_2=\xi\frac{K^m}{K^m+\bar{y}_2^m}$. This leads to a second order polynomial, $P(\bar{u}_1)=\bar{u}_1^2+A\bar{u}_1+B=0$, which admits a single positive real solution

$$\bar{u}_1=\frac{-A+\sqrt{A^2-4B}}{2},$$

where $A=\frac{f_2-f_1}{\delta}+\frac{\delta}{\gamma}$ and $B=-\frac{f_1}{\gamma}$.

6.3 Toggle switch and adaptive controller with positive feedback and U_2 actuation

We consider a modification of the previously proposed positive feedback architecture, where now the actuation is due to species U_2 (instead of U_1), as detailed in the main text and characterized in Fig. S9.

Chemical reactions

$\emptyset \xrightarrow{\alpha p_1} Y_1,$	$\emptyset \xrightarrow{\alpha p_2} Y_2,$	Production
$Y_1 \xrightarrow{\delta} \emptyset,$	$Y_2 \xrightarrow{\delta} \emptyset,$	Decay
$\emptyset \xrightarrow{\theta p_1} X$	$\emptyset \xrightarrow{\xi p_1} U_2$	Production
$U_2 \xrightarrow{\beta} U_2+Y_1$	$U_1+U_2 \xrightarrow{\gamma} \emptyset$	Production/Sequestration
$U_1, U_2 \xrightarrow{\delta} \emptyset$	$X \xrightarrow{\delta} \emptyset$	Decay

where $p_1=\frac{K^m}{y_2^m+K^m}$ and $p_2=\frac{K^m}{y_1^m+K^m}$.

Ordinary Differential Equations

$$\dot{y}_1 = \alpha\frac{K^m}{y_2^m+K^m}-\delta y_1+\beta u_2 \quad (36)$$

$$\dot{y}_2 = \alpha\frac{K^m}{y_1^m+K^m}-\delta y_2 \quad (37)$$

$$\dot{u}_1 = kx-\delta u_1-\gamma u_1 u_2 \quad (38)$$

$$\dot{u}_2 = \xi\frac{K^m}{K^m+y_2^m}-\delta u_2-\gamma u_1 u_2 \quad (39)$$

$$\dot{x} = \theta\frac{K^m}{K^m+y_2^m}-\delta x \quad (40)$$

Steady-state analysis

We can compute the system nullclines in the y_1-y_2 phase plane by equating $\dot{y}_1 = 0$ and $\dot{y}_2 = 0$, which yields the following expressions:

$$\begin{aligned}\bar{y}_2 &= \frac{\alpha}{\delta}\left(\frac{K^m}{K^m + \bar{y}_1^m}\right)\\ \bar{y}_1 &= \frac{\alpha}{\delta}\frac{K^m}{K^m + \bar{y}_2^m} + \frac{\beta}{\delta}u_2\end{aligned}$$

We can further find $\bar{u}_2$ as a function of $\bar{y}_2$. First, since at the equilibrium $\dot{x} = 0$, it is $\bar{x} = \frac{\theta}{\delta}\frac{K^m}{K^m+\bar{y}_2^m}$. Then, by considering both $\dot{u}_1 = 0$ and $\dot{u}_2 = 0$, we have

$$\bar{u}_1 = \frac{f_2 - \delta\bar{u}_2}{\gamma\bar{u}_2} = \frac{f_1}{\gamma\bar{u}_2 + \delta},$$

where $f_1 = k\bar{x}$ and $f_2 = \xi\frac{K^m}{K^m+\bar{y}_2^m}$. This leads to a second order polynomial, $P(\bar{u}_2) = \bar{u}_2^2 + A\bar{u}_2 + B = 0$, which admits a single positive real solution

$$\bar{u}_2 = \frac{-A + \sqrt{A^2 - 4B}}{2},$$

where $A = \frac{f_1 - f_2}{\delta} + \frac{\delta}{\gamma}$ and $B = -\frac{f_2}{\gamma}$.

6.4 Toggle switch and adaptive controller with negative feedback and U_2 actuation

We consider a modification of the previously proposed negative feedback architecture, where now the actuation is due to species U_2 (instead of U_1), as detailed in the main text and characterized in Fig. S10.

Chemical reactions

$\emptyset \xrightarrow{\alpha p_1} Y_1,$	$\emptyset \xrightarrow{\alpha p_2} Y_2,$	Production
$Y_1 \xrightarrow{\delta} \emptyset,$	$Y_2 \xrightarrow{\delta} \emptyset,$	Decay
$\emptyset \xrightarrow{\theta p_2} X$	$\emptyset \xrightarrow{\xi p_2} U_2$	Production
$U_2 \xrightarrow{\beta} U_2 + Y_1$	$U_1 + U_2 \xrightarrow{\gamma} \emptyset$	Production/Sequestration
$U_1, U_2 \xrightarrow{\delta} \emptyset$	$X \xrightarrow{\delta} \emptyset$	Decay

where $p_1 = \frac{K^m}{y_2^m + K^m}$ and $p_2 = \frac{K^m}{y_1^m + K^m}$.

Ordinary Differential Equations

$$\dot{y}_1 = \alpha\frac{K^m}{y_2^m + K^m} - \delta y_1 + \beta u_2 \tag{41}$$

$$\dot{y}_2 = \alpha\frac{K^m}{y_1^m + K^m} - \delta y_2 \tag{42}$$

$$\dot{u}_1 = kx - \delta u_1 - \gamma u_1 u_2 \tag{43}$$

$$\dot{u}_2 = \xi\frac{K^m}{K^m + y_1^m} - \delta u_2 - \gamma u_1 u_2 \tag{44}$$

$$\dot{x} = \theta\frac{K^m}{K^m + y_1^m} - \delta x \tag{45}$$

Steady-state analysis

We can compute the system nullclines in the y_1-y_2 phase plane by equating $\dot{y}_1 = 0$ and $\dot{y}_2 = 0$, which yields the following expressions:

$$\bar{y}_1 = \sqrt[m]{\frac{\alpha K^m}{\delta \bar{y}_2} - K^m}$$

$$\bar{y}_2 = \sqrt[m]{\frac{\alpha K^m}{\delta \bar{y}_1 - \beta \bar{u}_2} - K^m}$$

We can further find $\bar{u}_2$ as a function of $\bar{y}_1$. First, since at the equilibrium $\dot{x} = 0$, it is $\bar{x} = \frac{\theta}{\delta}\frac{K^m}{K^m+\bar{y}_1^m}$. Then, by considering both $\dot{u}_1 = 0$ and $\dot{u}_2 = 0$, we have

$$\bar{u}_1 = \frac{f_2 - \delta\bar{u}_2}{\gamma\bar{u}_2} = \frac{f_1}{\gamma\bar{u}_2 + \delta},$$

where $f_1 = k\bar{x}$ and $f_2 = \xi\frac{K^m}{K^m+\bar{y}_1^m}$. This leads to a second order polynomial, $P(\bar{u}_2) = \bar{u}_2^2 + A\bar{u}_2 + B = 0$, which admits a single positive real solution

$$\bar{u}_2 = \frac{-A + \sqrt{A^2 - 4B}}{2}$$

where $A = \frac{f_1 - f_2}{\delta} + \frac{\delta}{\gamma}$ and $B = -\frac{f_2}{\gamma}$.

6.5 Mutual activation system and adaptive controller

We report here chemical reactions, differential equations and steady-state analysis for the networks considered in Section 2.3.1 (Applying the adaptive control principle to a different system) in the main text, and characterized in Fig. S22.

6.5.1 Single inhibiting controller in a negative feedback with actuation through U_1 species

Chemical reactions

$\emptyset \xrightarrow{\alpha p_4} Y_1,$	$\emptyset \xrightarrow{\alpha p_3} Y_2,$	Production
$Y_1 \xrightarrow{\delta} \emptyset,$	$Y_2 \xrightarrow{\delta} \emptyset,$	Decay
$\emptyset \xrightarrow{\theta p_3} X,$	$\emptyset \xrightarrow{\xi p_3} U_2,$	Production
$Y_1 \xrightarrow{\beta p_5} \emptyset$	$U_1 + U_2 \xrightarrow{\gamma} \emptyset,$	Inhibition/Sequestration
$U_1, U_2 \xrightarrow{\delta} \emptyset$	$X \xrightarrow{\delta} \emptyset$	Decay

where $p_3 = \frac{y_1^m}{y_1^m+K^m}$, $p_4 = \frac{y_2^m}{y_2^m+K^m}$ and $p_5 = \frac{u_1}{y_1+K}$.

Ordinary Differential Equations

$$\dot{y}_1 = \alpha\frac{y_2^m}{y_2^m+K^m} - \delta y_1 - \beta u_1 \frac{y_1}{y_1+K} \quad (46)$$

$$\dot{y}_2 = \alpha\frac{y_1^m}{y_1^m+K^m} - \delta y_2 \quad (47)$$

$$\dot{u}_1 = kx - \delta u_1 - \gamma u_1 u_2 \quad (48)$$

$$\dot{u}_2 = \xi\frac{y_1^m}{K^m+y_1^m} - \delta u_2 - \gamma u_1 u_2 \quad (49)$$

$$\dot{x} = \theta\frac{y_1^m}{K^m+y_1^m} - \delta x \quad (50)$$

Steady-state analysis

We can compute the system nullclines in the y_1-y_2 phase plane by equating $\dot{y}_1 = 0$ and $\dot{y}_2 = 0$, which yields the following expressions:

$$\bar{y}_1 = \sqrt[m]{\frac{\delta \bar{y}_2 K^m}{\alpha - \delta \bar{y}_2}}$$

$$\bar{y}_2 = \sqrt[m]{\frac{hK^m}{\alpha - h}}$$

where $h = \delta\bar{y}_1 + \beta\bar{u}_1\frac{\bar{y}_1}{\bar{y}_1+K}$. We can further find $\bar{u}_1$ as a function of $\bar{y}_1$. First, since at the equilibrium $\dot{x} = 0$, it is $\bar{x} = \frac{\theta}{\delta}\frac{y_1^m}{K^m+\bar{y}_1^m}$. Then, by considering both $\dot{u}_1 = 0$ and $\dot{u}_2 = 0$, we have

$$\bar{u}_2 = \frac{f_1 - \delta\bar{u}_1}{\gamma\bar{u}_1} = \frac{f_2}{\gamma\bar{u}_1+\delta},$$

where $f_1 = k\bar{x}$ and $f_2 = \xi\frac{y_1^m}{K^m+\bar{y}_1^m}$. This leads to a second order polynomial, $P(\bar{u}_1) = \bar{u}_1^2 + A\bar{u}_1 + B = 0$, which admits a single positive real solution

$$\bar{u}_1 = \frac{-A+\sqrt{A^2-4B}}{2},$$

where $A = \frac{f_2-f_1}{\delta} + \frac{\delta}{\gamma}$ and $B = -\frac{f_1}{\gamma}$.

6.5.2 Single inhibiting controller in a positive feedback with actuation through U_1 species

Chemical reactions

$$
\begin{array}{lll}
\emptyset \xrightarrow{\alpha p_4} Y_1, & \emptyset \xrightarrow{\alpha p_3} Y_2, & \text{Production} \\
Y_1 \xrightarrow{\delta} \emptyset, & Y_2 \xrightarrow{\delta} \emptyset, & \text{Decay} \\
\emptyset \xrightarrow{\theta p_4} X, & \emptyset \xrightarrow{\xi p_4} U_2, & \text{Production} \\
Y_1 \xrightarrow{\beta p_5} \emptyset & U_1 + U_2 \xrightarrow{\gamma} \emptyset, & \text{Inhibition/Sequestration} \\
U_1, U_2 \xrightarrow{\delta} \emptyset & X \xrightarrow{\delta} \emptyset & \text{Decay}
\end{array}
$$

where $p_3 = \frac{y_1^m}{y_1^m + K^m}$, $p_4 = \frac{y_2^m}{y_2^m + K^m}$ and $p_5 = \frac{u_1}{y_1 + K}$.

Ordinary Differential Equations

$$
\begin{aligned}
\dot{y}_1 &= \alpha \frac{y_2^m}{y_2^m + K^m} - \delta y_1 - \beta u_1 \frac{y_1}{y_1 + K} \\
\dot{y}_2 &= \alpha \frac{y_1^m}{y_1^m + K^m} - \delta y_2 \\
\dot{u}_1 &= kx - \delta u_1 - \gamma u_1 u_2 \\
\dot{u}_2 &= \xi \frac{y_2^m}{K^m + y_2^m} - \delta u_2 - \gamma u_1 u_2 \\
\dot{x} &= \theta \frac{y_2^m}{K^m + y_2^m} - \delta x
\end{aligned}
$$

Steady-state analysis

We can compute the system nullclines in the y_1-y_2 phase plane by equating $\dot{y}_1 = 0$ and $\dot{y}_2 = 0$, which yields the following expressions:

$$
\begin{aligned}
\dot{y}_1 &= \alpha \frac{\bar{y}_2^m}{\bar{y}_2^m + K^m} - \delta \bar{y}_1 - \beta \bar{u}_1 \frac{\bar{y}_1}{\bar{y}_1 + K} = 0 \\
\bar{y}_2 &= \frac{\alpha}{\delta} \frac{\bar{y}_1^m}{\bar{y}_1^m + K^m}
\end{aligned}
$$

The solution of $\dot{y}_1 = 0$ in terms of $\bar{y}_1$ has a complex closed-form analytical expression, so we applied numerical methods. Since the adaptive controller is essentially an exogenous circuit, we can still find $\bar{u}_1$ as a function of $\bar{y}_2$. First, since at the equilibrium $\dot{x} = 0$, it is $\bar{x} = \frac{\theta}{\delta} \frac{y_2^m}{K^m + \bar{y}_2^m}$. Then, by considering both $\dot{u}_1 = 0$ and $\dot{u}_2 = 0$, we have

$$
\bar{u}_2 = \frac{f_1 - \delta \bar{u}_1}{\gamma \bar{u}_1} = \frac{f_2}{\gamma \bar{u}_1 + \delta},
$$

where $f_1 = k\bar{x}$ and $f_2 = \xi \frac{y_2^m}{K^m + \bar{y}_2^m}$. This leads to a second order polynomial, $P(\bar{u}_1) = \bar{u}_1^2 + A\bar{u}_1 + B = 0$, which admits a single positive real solution

$$\bar{u}_1 = \frac{-A + \sqrt{A^2 - 4B}}{2}$$

where $A = \frac{f_2 - f_1}{\delta} + \frac{\delta}{\gamma}$ and $B = -\frac{f_1}{\gamma}$.

6.5.3 Single inhibiting controller in a negative feedback with actuation through U_2 species

Chemical reactions

$\emptyset \xrightarrow{\alpha p_4} Y_1,$	$\emptyset \xrightarrow{\alpha p_3} Y_2,$	Production
$Y_1 \xrightarrow{\delta} \emptyset,$	$Y_2 \xrightarrow{\delta} \emptyset,$	Decay
$\emptyset \xrightarrow{\theta p_3} X,$	$\emptyset \xrightarrow{\xi p_3} U_2,$	Production
$Y_1 \xrightarrow{\beta p_5} \emptyset$	$U_1 + U_2 \xrightarrow{\gamma} \emptyset,$	Inhibition/Sequestration
$U_1, U_2 \xrightarrow{\delta} \emptyset$	$X \xrightarrow{\delta} \emptyset$	Decay

where $p_3 = \frac{y_1^m}{y_1^m + K^m}$, $p_4 = \frac{y_2^m}{y_2^m + K^m}$ and $p_5 = \frac{u_2}{y_1 + K}$.

Ordinary Differential Equations

$$\dot{y}_1 = \alpha \frac{y_2^m}{y_2^m + K^m} - \delta y_1 - \beta u_2 \frac{y_1}{y_1 + K} \tag{51}$$

$$\dot{y}_2 = \alpha \frac{y_1^m}{y_1^m + K^m} - \delta y_2 \tag{52}$$

$$\dot{u}_1 = kx - \delta u_1 - \gamma u_1 u_2 \tag{53}$$

$$\dot{u}_2 = \xi \frac{y_1^m}{K^m + y_1^m} - \delta u_2 - \gamma u_1 u_2 \tag{54}$$

$$\dot{x} = \theta \frac{y_1^m}{K^m + y_1^m} - \delta x \tag{55}$$

Steady-state analysis

We can compute the system nullclines in the y_1-y_2 phase plane by equating $\dot{y}_1 = 0$ and $\dot{y}_2 = 0$, which yields the following expressions:

$$\bar{y}_1 = \sqrt[m]{\frac{\delta \bar{y}_2 K^m}{\alpha - \delta \bar{y}_2}}$$

$$\bar{y}_2 = \sqrt[m]{\frac{h K^m}{\alpha - h}}$$

where $h = \delta \bar{y}_1 + \beta \bar{u}_2 \frac{\bar{y}_1}{\bar{y}_1 + K}$. We can further find $\bar{u}_2$ as a function of $\bar{y}_1$. First, since at the equilibrium $\dot{x} = 0$, it is $\bar{x} = \frac{\theta}{\delta} \frac{y_1^m}{K^m + \bar{y}_1^m}$. Then, by considering both $\dot{u}_1 = 0$ and $\dot{u}_2 = 0$, we have

$$\bar{u}_1 = \frac{f_2 - \delta \bar{u}_2}{\gamma \bar{u}_2} = \frac{f_1}{\gamma \bar{u}_2 + \delta},$$

where $f_1 = k\bar{x}$ and $f_2 = \xi\frac{y_1^m}{K^m+\bar{y}_1^m}$. This leads to a second order polynomial, $P(\bar{u}_2) = \bar{u}_2^2 + A\bar{u}_2 + B = 0$, which admits a single positive real solution

$$\bar{u}_2 = \frac{-A + \sqrt{A^2 - 4B}}{2},$$

where $A = \frac{f_1 - f_2}{\delta} + \frac{\delta}{\gamma}$ and $B = -\frac{f_2}{\gamma}$.

6.5.4 Double inhibiting controller with actuation through U_1 and U_1' species

The species forming the controller that actuates over Y_1 are U_1, U_2 and X, while the species forming the controller that actuates over Y_2 are U_1', U_2' and X'. We assumed the same kinetic constants (δ, k, ξ, θ and γ) for both controllers, except for the actuation gain, which is β and β', respectively.

Chemical reactions

$\emptyset \xrightarrow{\alpha p_4} Y_1,$	$\emptyset \xrightarrow{\alpha p_3} Y_2,$	Production
$Y_1 \xrightarrow{\delta} \emptyset,$	$Y_2 \xrightarrow{\delta} \emptyset,$	Decay
$\emptyset \xrightarrow{\theta p_3} X$	$\emptyset \xrightarrow{\xi p_3} U_2$	Production
$Y_1 \xrightarrow{\beta p_5} \emptyset$	$U_1 + U_2 \xrightarrow{\gamma} \emptyset$	Inhibition/Sequestration
$U_1, U_2 \xrightarrow{\delta} \emptyset$	$X \xrightarrow{\delta} \emptyset$	Decay
$\emptyset \xrightarrow{\theta p_4} X'$	$\emptyset \xrightarrow{\xi p_4} U_2'$	Production
$Y_2 \xrightarrow{\beta' p_6} \emptyset$	$U_1' + U_2' \xrightarrow{\gamma} \emptyset$	Inhibition/Sequestration
$U_1', U_2' \xrightarrow{\delta} \emptyset$	$X' \xrightarrow{\delta} \emptyset$	Decay

where $p_3 = \frac{y_1^m}{y_1^m+K^m}$, $p_4 = \frac{y_2^m}{y_2^m+K^m}$, $p_5 = \frac{u_1}{y_1+K}$ and $p_6 = \frac{u_1'}{y_2+K}$.

Ordinary Differential Equations

$$\dot{y}_1 = \alpha\frac{y_2^m}{y_2^m + K^m} - \delta y_1 - \beta u_1 \frac{y_1}{y_1 + K} \quad (56)$$

$$\dot{y}_2 = \alpha\frac{y_1^m}{y_1^m + K^m} - \delta y_2 - \beta u_1' \frac{y_2}{y_2 + K} \quad (57)$$

$$\dot{u}_1 = kx - \delta u_1 - \gamma u_1 u_2 \quad (58)$$

$$\dot{u}_2 = \xi\frac{y_1^m}{K^m + y_1^m} - \delta u_2 - \gamma u_1 u_2 \quad (59)$$

$$\dot{x} = \theta\frac{y_1^m}{K^m + y_1^m} - \delta x \quad (60)$$

$$\dot{u}_1' = kx' - \delta u_1' - \gamma u_1' u_2' \quad (61)$$

$$\dot{u}_2' = \xi\frac{y_2^m}{K^m + y_2^m} - \delta u_2' - \gamma u_1' u_2' \quad (62)$$

$$\dot{x}' = \theta\frac{y_2^m}{K^m + y_2^m} - \delta x' \quad (63)$$

Steady-state analysis

We can compute the system nullclines in the y_1-y_2 phase plane by equating $\dot{y}_1 = 0$ and $\dot{y}_2 = 0$, which yields the following expressions:

$$\begin{aligned} \bar{y}_1 &= \sqrt[m]{\frac{h_1 K^m}{\alpha - h_1}} \\ \bar{y}_2 &= \sqrt[m]{\frac{h_2 K^m}{\alpha - h_2}} \end{aligned}$$

where $h_1 = \delta \bar{y}_2 + \beta \bar{u}_1' \frac{\bar{y}_2}{\bar{y}_2 + K^m}$ and $h_2 = \delta \bar{y}_1 + \beta \bar{u}_1 \frac{\bar{y}_1}{\bar{y}_1 + K^m}$. As detailed before, $\bar{u}_1$ and $\bar{u}_1'$ can be expressed as functions of $\bar{y}_1$ and $\bar{y}_2$, respectively.

6.6 Toggle switch with self-activation and adaptive controller with negative feedback

We report here chemical reactions, differential equations and steady-state analysis for the networks considered in Section 2.3.2 (Applying the adaptive control principle to multistable systems) in the main text, and characterized in Fig. S23.

6.6.1 Double inhibiting controller with actuation through U_1 and U_1' species

Chemical reactions

$\emptyset \xrightarrow{\alpha p_1} Y_1,\ \emptyset \xrightarrow{\alpha p_3} Y_1,$	$\emptyset \xrightarrow{\alpha p_2} Y_2,\ \emptyset \xrightarrow{\alpha p_4} Y_2,$	Production
$Y_1 \xrightarrow{\delta} \emptyset,$	$Y_2 \xrightarrow{\delta} \emptyset,$	Decay
$\emptyset \xrightarrow{\theta p_2} X$	$\emptyset \xrightarrow{\xi p_2} U_2$	Production
$Y_1 \xrightarrow{\beta p_5} \emptyset$	$U_1 + U_2 \xrightarrow{\gamma} \emptyset$	Inhibition/Sequestration
$U_1, U_2 \xrightarrow{\delta} \emptyset$	$X \xrightarrow{\delta} \emptyset$	Decay
$\emptyset \xrightarrow{\theta p_1} X'$	$\emptyset \xrightarrow{\xi p_1} U_2'$	Production
$Y_2 \xrightarrow{\beta' p_6} \emptyset$	$U_1' + U_2' \xrightarrow{\gamma} \emptyset$	Inhibition/Sequestration
$U_1', U_2' \xrightarrow{\delta} \emptyset$	$X' \xrightarrow{\delta} \emptyset$	Decay

where $p_1 = \frac{K^m}{y_2^m + K^m}$, $p_2 = \frac{K^m}{y_1^m + K^m}$, $p_3 = \frac{y_1^m}{y_1^m + K^m}$, $p_4 = \frac{y_2^m}{y_2^m + K^m}$, $p_5 = \frac{u_1}{y_1 + K}$ and $p_6 = \frac{u_1'}{y_2 + K}$.

Ordinary Differential Equations

$$\dot{y}_1 = \alpha\frac{K^m}{y_2^m + K^m} + \alpha\frac{y_1^m}{y_1^m + K^m} - \delta y_1 - \beta u_1\frac{y_1}{y_1 + K} \quad (64)$$

$$\dot{y}_2 = \alpha\frac{K^m}{y_1^m + K^m} + \alpha\frac{y_2^m}{y_2^m + K^m} - \delta y_2 - \beta u_1'\frac{y_2}{y_2 + K} \quad (65)$$

$$\dot{u}_1 = kx - \delta u_1 - \gamma u_1 u_2 \quad (66)$$

$$\dot{u}_2 = \xi\frac{K^m}{K^m + y_1^m} - \delta u_2 - \gamma u_1 u_2 \quad (67)$$

$$\dot{x} = \theta\frac{K^m}{K^m + y_1^m} - \delta x \quad (68)$$

$$\dot{u}_1' = kx' - \delta u_1' - \gamma u_1' u_2' \quad (69)$$

$$\dot{u}_2' = \xi\frac{K^m}{K^m + y_2^m} - \delta u_2' - \gamma u_1' u_2' \quad (70)$$

$$\dot{x}' = \theta\frac{K^m}{K^m + y_2^m} - \delta x' \quad (71)$$

Steady-state analysis

We can compute the system nullclines in the y_1-y_2 phase plane by equating $\dot{y}_1 = 0$ and $\dot{y}_2 = 0$, which yields the following expressions:

$$\bar{y}_1 = \sqrt[m]{\frac{\alpha K^m}{h_1} - K^m}$$

$$\bar{y}_2 = \sqrt[m]{\frac{\alpha K^m}{h_2} - K^m}$$

where $h_1 = \delta\bar{y}_2 + \frac{\bar{y}_2^m}{\bar{y}_2^m + K^m}(\beta\bar{u}_1' - \alpha)$ and $h_2 = \delta\bar{y}_1 + \frac{\bar{y}_1^m}{\bar{y}_1^m + K^m}(\beta\bar{u}_1 - \alpha)$. As detailed before, $\bar{u}_1$ and $\bar{u}_1'$ can be expressed as functions of $\bar{y}_1$ and $\bar{y}_2$, respectively.

6.6.2 Inhibition-activation pair

Chemical reactions

$\emptyset \xrightarrow{\alpha p_1} Y_1,\ \emptyset \xrightarrow{\alpha p_3} Y_1,$	$\emptyset \xrightarrow{\alpha p_2} Y_2,\ \emptyset \xrightarrow{\alpha p_4} Y_2,$	Production
$Y_1 \xrightarrow{\delta} \emptyset,$	$Y_2 \xrightarrow{\delta} \emptyset,$	Decay
$\emptyset \xrightarrow{\theta p_2} X$	$\emptyset \xrightarrow{\xi p_2} U_2$	Production
$U_1 \xrightarrow{\beta} U_1 + Y_1$	$U_1 + U_2 \xrightarrow{\gamma} \emptyset$	Production/Sequestration
$U_1, U_2 \xrightarrow{\delta} \emptyset$	$X \xrightarrow{\delta} \emptyset$	Decay
$\emptyset \xrightarrow{\theta p_1} X'$	$\emptyset \xrightarrow{\xi p_1} U_2'$	Production
$Y_2 \xrightarrow{\beta' p_6} \emptyset$	$U_1' + U_2' \xrightarrow{\gamma} \emptyset$	Inhibition/Sequestration
$U_1', U_2' \xrightarrow{\delta} \emptyset$	$X' \xrightarrow{\delta} \emptyset$	Decay

where $p_1 = \frac{K^m}{y_2^m + K^m}$, $p_2 = \frac{K^m}{y_1^m + K^m}$, $p_3 = \frac{y_1^m}{y_1^m + K^m}$, $p_4 = \frac{y_2^m}{y_2^m + K^m}$ and $p_6 = \frac{u_1'}{y_2 + K}$.

Ordinary Differential Equations

$$\begin{aligned}
\dot{y}_1 &= \alpha\frac{K^m}{y_2^m + K^m} + \alpha\frac{y_1^m}{y_1^m + K^m} - \delta y_1 + \beta u_1 && (72)\\
\dot{y}_2 &= \alpha\frac{K^m}{y_1^m + K^m} + \alpha\frac{y_2^m}{y_2^m + K^m} - \delta y_2 - \beta u_1'\frac{y_2}{y_2 + K} && (73)\\
\dot{u}_1 &= kx - \delta u_1 - \gamma u_1 u_2 && (74)\\
\dot{u}_2 &= \xi\frac{K^m}{K^m + y_1^m} - \delta u_2 - \gamma u_1 u_2 && (75)\\
\dot{x} &= \theta\frac{K^m}{K^m + y_1^m} - \delta x && (76)\\
\dot{u}_1' &= kx' - \delta u_1' - \gamma u_1' u_2' && (77)\\
\dot{u}_2' &= \xi\frac{K^m}{K^m + y_2^m} - \delta u_2' - \gamma u_1' u_2' && (78)\\
\dot{x}' &= \theta\frac{K^m}{K^m + y_2^m} - \delta x' && (79)
\end{aligned}$$

Steady-state analysis

We can compute the system nullclines in the y_1-y_2 phase plane by equating $\dot{y}_1 = 0$ and $\dot{y}_2 = 0$, which yields the following expressions:

$$\begin{aligned}
\bar{y}_1 &= \sqrt[m]{\frac{h_1 K^m}{\alpha - h_1}}\\
\bar{y}_2 &= \sqrt[m]{\frac{h_2 K^m}{\alpha - h_2}}
\end{aligned}$$

where $h_1 = \delta\bar{y}_2 + \beta\bar{u}_1'\frac{\bar{y}_2}{\bar{y}_2+K} - \alpha\frac{\bar{y}_2^m}{\bar{y}_2^m+K^m}$ and $h_2 = \delta y_1 - \beta\bar{u}_1 - \alpha\frac{\bar{y}_1^m}{\bar{y}_1^m+K^m}$. As detailed before, $\bar{u}_1$ and $\bar{u}_1'$ can be expressed as functions of $\bar{y}_1$ and $\bar{y}_2$, respectively.

References

[1] Sagar, Grün D. Deciphering Cell Fate Decision by Integrated Single-Cell Sequencing Analysis. Annual Review of Biomedical Data Science. 2020 Jul;3(1):1-22. Available from: https://doi.org/10.1146/annurev-biodatasci-111419-091750.

[2] Semrau S, van Oudenaarden A. Studying Lineage Decision-Making In Vitro: Emerging Concepts and Novel Tools. Annual Review of Cell and Developmental Biology. 2015 Nov;31(1):317–345. Available from: http://dx.doi.org/10.1146/annurev-cellbio-100814-125300.

[3] Shakiba N, Jones RD, Weiss R, Del Vecchio D. Context-aware synthetic biology by controller design: Engineering the mammalian cell. Cell Systems. 2021 Jun;12(6):561–592. Available from: http://dx.doi.org/10.1016/j.cels.2021.05.011.

[4] Kamimoto K, Stringa B, Hoffmann CM, Jindal K, Solnica-Krezel L, Morris SA. Dissecting cell identity via network inference and in silico gene perturbation. Nature. 2023 Feb;614(7949):742–751. Available from: http://dx.doi.org/10.1038/s41586-022-05688-9.

[5] Qiu X, Zhang Y, Martin-Rufino JD, Weng C, Hosseinzadeh S, Yang D, et al. Mapping transcriptomic vector fields of single cells. Cell. 2022 Feb;185(4):690-711.e45. Available from: http://dx.doi.org/10.1016/j.cell.2021.12.045.

[6] Huang S, Eichler G, Bar-Yam Y, Ingber DE. Cell Fates as High-Dimensional Attractor States of a Complex Gene Regulatory Network. Physical Review Letters. 2005 Apr;94(12). Available from: http://dx.doi.org/10.1103/PhysRevLett.94.128701.

[7] Hinman VF, Nguyen AT, Cameron RA, Davidson EH. Developmental gene regulatory network architecture across 500 million years of echinoderm evolution. Proceedings of the National Academy of Sciences. 2003 Oct;100(23):13356-61. Available from: https://doi.org/10.1073/pnas.2235868100.

[8] Laslo P, Spooner CJ, Warmflash A, Lancki DW, Lee HJ, Sciammas R, et al. Multilineage Transcriptional Priming and Determination of Alternate Hematopoietic Cell Fates. Cell. 2006 Aug;126(4):755-66. Available from: https://doi.org/10.1016/j.cell.2006.06.052.

[9] MacArthur BD, Ma'ayan A, Lemischka IR. Systems biology of stem cell fate and cellular reprogramming. Nature Reviews Molecular Cell Biology. 2009 Sep;10(10):672-81. Available from: https://doi.org/10.1038/nrm2766.

[10] Trunnell NB, Poon AC, Kim SY, Ferrell JE. Ultrasensitivity in the Regulation of Cdc25C by Cdk1. Molecular Cell. 2011 Feb;41(3):263-74. Available from: https://doi.org/10.1016/j.molcel.2011.01.012.

[11] Yao G, Tan C, West M, Nevins JR, You L. Origin of bistability underlying mammalian cell cycle entry. Molecular Systems Biology. 2011 Jan;7(1). Available from: http://dx.doi.org/10.1038/msb.2011.19.

[12] Wang N, Liang H, Zen K. Molecular Mechanisms That Influence the Macrophage M1â€“M2 Polarization Balance. Frontiers in Immunology. 2014 Nov;5. Available from: http://dx.doi.org/10.3389/fimmu.2014.00614.

[13] Lee TI, Rinaldi NJ, Robert F, Odom DT, Bar-Joseph Z, Gerber GK, et al. Transcriptional Regulatory Networks in Saccharomyces cerevisiae. Science. 2002 Oct;298(5594):799-804. Available from: https://doi.org/10.1126/science.1075090.

[14] Elowitz MB, Levine AJ, Siggia ED, Swain PS. Stochastic Gene Expression in a Single Cell. Science. 2002 Aug;297(5584):1183–1186. Available from: http://dx.doi.org/10.1126/science.1070919.

[15] Soldatov R, Kaucka M, Kastriti ME, Petersen J, Chontorotzea T, Englmaier L, et al. Spatiotemporal structure of cell fate decisions in murine neural crest. Science. 2019 Jun;364(6444). Available from: https://doi.org/10.1126/science.aas9536.

[16] Kramer BA, Sarabia del Castillo J, Pelkmans L. Multimodal perception links cellular state to decision-making in single cells. Science. 2022 Aug;377(6606):642–648. Available from: http://dx.doi.org/10.1126/science.abf4062.

[17] Tran M, Askary A, Elowitz MB. Lineage motifs as developmental modules for control of cell type proportions. Developmental Cell. 2024 Mar;59(6):812-26.e3. Available from: http://dx.doi.org/10.1016/j.devcel.2024.01.017.

[18] Zhang Y, Cuba Samaniego C, Carleton K, Qian Y, Giordano G, Franco E. Building molecular band-pass filters via molecular sequestration. In: 2022 IEEE 61st Conference on Decision and Control (CDC). IEEE; 2022. Available from: https://doi.org/10.1109%2Fcdc51059.2022.9993401.

[19] Cuba Samaniego C, Qian Y, Carleton K, Franco E. Building Subtraction Operators and Controllers Via Molecular Sequestration. IEEE Control Systems Letters. 2023:1–1. Available from: http://dx.doi.org/10.1109/LCSYS.2023.3294690.

[20] Guilberteau J, Pouchol C, Pouradier Duteil N. Monostability and bistability of biological switches. Journal of Mathematical Biology. 2021 Nov;83(6–7). Available from: http://dx.doi.org/10.1007/s00285-021-01687-y.
